# Exploratory screening for micro-RNA biomarkers in canine multicentric lymphoma

**DOI:** 10.3389/fvets.2024.1379146

**Published:** 2024-05-17

**Authors:** Sabine E. Hammer, Julia Sprung, Ondřej Škor, Stefanie Burger, Martin Hofer, Ilse Schwendenwein, Barbara C. Rütgen

**Affiliations:** ^1^Immunology, Department of Biological Sciences and Pathobiology, University of Veterinary Medicine Vienna, Vienna, Austria; ^2^Laboklin GMBH & CO.KG, Bad Kissingen, Germany; ^3^VetBioBank, VetCore, University of Veterinary Medicine Vienna, Vienna, Austria; ^4^Genomics Core Facility, VetCore, University of Veterinary Medicine Vienna, Vienna, Austria; ^5^Clinical Pathology, Department of Biological Sciences and Pathobiology, University of Veterinary Medicine Vienna, Vienna, Austria

**Keywords:** *Canis lupus familiaris*, diffuse large B-cell lymphoma (DLBCL), peripheral T-cell lymphoma (PTCL), microRNA expression analysis, potential biomarker candidates, animal model, biomedical research and development

## Abstract

Lymphoma is one of the most frequent hematopoietic tumors in dogs and shares similar features with human counterparts. MicroRNAs (miRNA, small non-coding RNAs) are pivotal in gene regulation fine tuning and cancer hallmarks are influenced by their aberrant expression. Consequently, miRNA biomarkers may assist predicting therapeutic response and clinical outcome by providing less-invasive novel diagnostics tools. The aim of this study was to detect dysregulated miRNAs in lymphomatous lymph node tissues in comparison to lymph node material or PBMCs from healthy control dogs. Potential significant differences in miRNA expression profiles between four lymphoma entities were evaluated. A customized PCR array was utilized to profile 89 canine target miRNAs. Quantification was performed using qPCR, relative expression was determined by the delta–delta Ct method, and *p*-values were calculated with student’s *t*-test. In the 14 diffuse large B-cell lymphoma (DLBCL) patients, 28 and 24 different miRNAs were significantly dysregulated compared to lymph node material or PBMCs. Sixteen miRNAs occurred in both control groups, with 12 miRNAs being down- and four miRNAs being upregulated. The six peripheral T-cell lymphoma (PTCL) samples showed 24 and 25 dysregulated miRNAs when compared to the healthy controls. A combined analysis of DLBCL and PTCL samples revealed seven shared and 19 differently expressed miRNAs. Potential biomarkers in T- and B-cell lymphoma could be the miRNA-17/92 cluster and miRNA-181-family together with miRNA-34a and miRNA-150. Diagnostic utility of potential biomarkers must be validated in larger, prospective cohorts of canine lymphoma cases and in higher numbers of physiological patient material.

## 1 Introduction

Lymphoma is one of the most common occurring tumors in dogs (1–4). The disease shows a prevalence of around 100 cases per 100.000 dogs and 83–85% of all canine hematopoietic neoplasias are represented by lymphoma (2, 4). This tumor initiates from lymphoid cells which are B- and T-cells (4–7). The proportions of lymphomas resulting from those cells are approximately 70% of B-cell and 30% of T-cell lymphoma (8, 9). Since all pure and mixed breeds are affected by lymphoma, this proportion can vary. However, some breeds are prone to develop a certain type of canine lymphoma, for instance, Boxers tend to develop T-cell lymphoma, whereas Golden Retrievers have an equal likelihood of developing B-cell as well as T-cell lymphoma (8–10). Canine lymphoma is a heterogeneous disease which primarily occurs in lymphatic organs, such as lymph nodes or spleen (6). The most prominent clinical presentation of lymphoma is multicentric lymphoma, which is characterized by generalized lymphadenopathy (2, 3, 9–11). The most dominant multicentric lymphoma which is also the most common subtype found in dogs is the diffuse large B-cell lymphoma (DLBCL) which will be characterized in more detail below (7, 8). It represents about 50–60% of all canine entities (8, 12). Beside the multicentric form, also extranodal anatomic forms exist. Most common are the alimentary, mediastinal, and cutaneous lymphoma. All three types originate either exclusively or typically from T-cells (9, 10).

In this retrospective study, four types of canine lymphoma will be analyzed. The DLBCL is as mentioned before the most common lymphoma entity in dogs also being the case for the studied cohort (13–16). The second subtype of interest represents the entity marginal zone lymphoma (MZL). This indolent B-cell lymphoma mainly occurs in the spleen and shares CD79a and CD20 expression with DLBCL in immunohistochemistry (IHC) but lacks CD3 expression (13, 14, 17, 18). In contrast, T-cell lymphoma (PTCL) shows expression of CD3 by lacking CD79a and CD20, characterizing them as T-cell type in IHC (13, 14, 19, 20). Finally, the subtype T-zone lymphoma (TZL), is an indolent low-grade T-cell lymphoma, in which frequent CD25 and CD3 expression is accompanied by missing CD45, CD79a and CD20. In flow cytometry this entity shows a unique phenotype being CD45^−^, CD5^+^ and CD21^−^ (13, 14, 18, 20, 21).

Canine lymphoma shares many similarities with the human non-Hodgkin lymphoma (NHL) in terms of their immunophenotypic composition, diagnosis, clinical presentation, treatment methods and molecular biology (3–5, 8, 9, 22). Furthermore, humans and dogs are both exposed to the same environmental and therefore the same risk factors that promote the development of cancers (3, 9, 22). Those environmental exposure include, for instance, household chemicals and polluted sites (9). Due to those similarities between the dog and their human counterpart, canine lymphoma is seen as a potential animal model for humans. Thus, new findings would be beneficial for both veterinary and human medicine especially concerning new diagnostic methods (1, 4, 5, 11).

MicroRNAs (miRNA, miR) are physiological occurring small non-coding RNA molecules which amount to approximately 18–25 nucleotides per miRNA. MicroRNAs are transcribed from individual genes which are located between introns and exons. They are widely expressed in all tissues, organs, and cells of a multicellular organism (1–4, 11, 22–25). However, some few miRNAs are restricted to certain cell types (22). Although miRNAs are unable to code for proteins, they regulate gene expressions in the post-transcription process through binding in 3’UTR of target mRNA (4, 22, 24). Each miRNA has the ability to control hundreds of targets for almost all pathways (1, 3, 11, 24). They are part of modification processes including cell differentiation, stress resistance, metabolism, cell cycle progression and apoptosis in normal cells (1, 7, 22). However, in neoplastic cells miRNAs are dysregulated and therefore, they have an effect on the functional roles in cancer cells, such as the initiation, progression and metastasis (2, 3, 25). MicroRNAs in cancer cells are either upregulated or downregulated in comparison to healthy cells, depending on if those miRNAs act as an oncogene or tumor suppressor (1, 3, 4, 7, 22). As a matter of fact, some even have a dual-function depending on the cell type (3). Those dysregulated miRNAs have the potential to be used as biomarkers for detection, classification or even prediction of tumors (1).

The diagnosis of canine lymphoma is challenging to achieve rapid and accurate results (7). The standard procedures nowadays focus on a morphological evaluation of biopsies as well as a cytologic examination of clinically suspect lymph nodes or other mostly lymphoid organs (3, 4, 7). To differentiate between the lymphoma entities, immunophenotyping, grading and clinical staging are necessary for tailored treatment decisions and prognostic information (3). For some common entities, as the large B-cell lymphoma (LBCL), peripheral T-cell lymphoma (PTCL) or peripheral T-zone lymphoma (PTZL), this currently can be provided by diagnostic flow cytometry (4, 7, 26–28). However, histopathological evaluation including World Health Organization (WHO) classification of a biopsy sample is still the diagnostic gold standard (13). As a matter of fact, the gold standard methods for diagnosis of canine lymphoma are rather time-consuming (7). The general presence, high stability and the minimally invasive material extraction of miRNAs might offer promising biomarkers as a new diagnostic approach for canine lymphomas (1–4, 7, 29). Furthermore, this reliable characterized material harboring miRNAs has the potential for getting deeper insight into genetic background, origin and cause for neoplastic development and differences in all different entities (3).

Compared to healthy dogs, the miRNA profile of the canine lymphoma-affected dogs is not thoroughly studied. To discover differentially expressed miRNAs in canine lymphoma, we utilized 24 archival cryopreserved lymph nodes, representing four canine lymphoma entities and material from eight normal healthy dogs. The identification and quantification of dysregulated miRNAs were assessed by applying the “qPCR-based miRCURY custom assay/panel system” (Qiagen) targeting a custom-made panel which consisted of 89 miRNAs that were selected from various literature sources. This retrospective study should deepen the understanding of entity-specific miRNAs expression profiles, which could be further developed into valuable diagnostic tools in dogs. By means of this study we could also improve our understanding of disease mechanisms, molecular pathways, biomarkers discovery, dysregulated miRNAs, and personalized medicine.

## 2 Materials and methods

### 2.1 Lymphoma patients and non-neoplastic control material

In this retrospective pilot study, all samples used here have already been analyzed regarding clinics, cytology, histopathology, immunophenotyping using flow cytometry and PCR for clonality testing (16). The examination material concerning the lymphoma patients consisted of 24 archival cryopreserved lymph nodes, representing four canine lymphoma entities: 14 samples of diffuse large B-cell lymphoma (DLBCL), six samples of Peripheral T-cell lymphoma (PTCL) including one enteric T-cell lymphoma (ent. TCL), as well as two samples each of Marginal zone lymphoma (MZL) and T-zone lymphoma (TZL) (16). The samples were provided by the archive of the VetBiobank (Vetmeduni), which have been already diagnosed as already mentioned by flow cytometry, clonality testing, histopathology including WHO classification and immunohistochemistry (16, 30). Lymph node sample material was available in sample duplicates. The solid lymph node pieces were on average approximately 2.5 × 2.5 × 2.5 mm in size and were stored in RNA-Later (Qiagen GmbH, Hilden, Germany). One sample per duplicate was processed and the second was stored as a backup in case of need for repetition. The detailed list of patients can be found in Supplementary Table 1. The non-neoplastic and non-inflammatory control material consisted of eight samples: four samples not-lymphoma bearing canine lymph node material and four samples canine peripheral blood mononuclear cells (PBMCs; Supplementary Table 2). The material consisted of archive material being characterized and immunophenotyped in course of a previous study (31). The lymph node material was provided in single cell suspension vials of about 1×10^7^ cells in freezing medium 50% RPMI 1640 medium (PAA, Pasching, Austria), 40% FCS (PAA, Pasching, Austria), 10% DMSO (Sigma-Aldrich, Austria). The PBMC were stored in freezing medium with a cell count between 5×10^6^–1×10^7^ cells. Since the pilot project was a retrospective study and all samples and controls have already been used in previous studies, no additional ethics approval was necessary (16, 31).

### 2.2 MicroRNA expression analysis

Prior to miRNA extraction and cDNA synthesis, control spike-ins were pre-prepared by using the “RNA Spike-in Kit for RT” (Qiagen) following the manufacturer’s instructions (32). It was used as a control to ensure a qualitative RNA isolation, cDNA synthesis as well as qPCR amplification. Next, miRNA was extracted from all examination materials by using the “miRNeasy Tissue/cells Advanced Micro Kit” (Qiagen) according to the manual supplied with the kit (33). After extraction, miRNA concentrations (ng/μL) and purity of miRNA (ratios: 260/280 and 260/230) were measured by using the Nanodrop 2000c (Thermo Fisher Scientific, Waltham, MA, United States). Afterwards, 20 ng of the extracted miRNAs were transformed into cDNA by using the “miCURY LNA RT Kit” (Qiagen) according to protocol (34). The identification and quantification of dysregulated miRNAs were assessed by applying the “qPCR-based miRCURY custom assay/panel system” (Qiagen) targeting a custom-made panel which consisted of 89 miRNAs that were selected from various literature sources (Supplementary Table 3) (1–3, 5, 7, 11, 22, 24, 25). Alongside with the patient samples and control groups, validated primer sets were used for normalization of miRNA expression levels in qPCR analyses (Qiagen). The detection was performed on “miRCURY LNA miRNA Custom PCR Panels” (Qiagen) consisting of 384-well plates. Each plate had the capacity for the assessment of four samples. The probes were analyzed in a 384er Cycler ViiA^™^ 7 qPCR System (Thermo Fisher Scientific).

### 2.3 Evaluation of data

The web-based “miRCURY LNA miRNA Expression Analysis” platform (Qiagen) was used for the data analysis of the raw data from this project. The Ct-values were transferred to an appropriate input file, excluding four miRNAs based on their percentage of which their Ct value was greater than 45 in the studied samples: miR-122 (69.44%), miR-127 (36.11%), miR-206 (41.67%), and miR-8908a-3p (80.56%). The first step of data analysis included grouping the samples according to their entities while the PBMCs and lymph nodes were selected as “control group” for comparing pathological samples to PBMCs and lymph nodes samples separately. Prior to statistical data evaluation, normalization of miRNAs was conducted by using the geNorm method (Supplementary Table 4). GeNorm is a normalization method based on a same expression ratio of the predefined reference miRNAs by using standard deviation of log-transformed reference miRNA ratios which should be identical in all samples. The stability factor which should be below 1.5 shows an average pairwise variation between one miRNA compared to all other reference miRNAs (35, 36). MiR-16, miR-21, miR-22, miR-146a, and miR-350 were the most stable expressed miRNAs across all samples and were used as endogenous controls to normalize differences in the patient samples (35). After normalization, the parameter Fold change, Fold regulation and *p*-value were calculated based on the ΔΔCt method (Fold change: 2^(-ΔΔCt)^) (36). Fold regulation and Fold change are identical in terms of their information. However, data is presented differently in terms of a downregulation of the miRNA. While in Fold changes a decimal number between 0 and 1 is shown, it is presented as a negative inverse fraction in Fold regulation (37). *p*-values were calculated by using the student’s t-test to the linearized normalized miRNA expression levels for each miRNA in each group (control group as well as test group) by assuming an equal variance. As for the significance of the *p*-value, the threshold was set to 0.05 (37). For data visualization, bar charts were used to show dysregulations of the lymphoma entities in comparison to each control group. Furthermore, scatter plots were used to compare the normalized expression levels of each miRNA between two defined groups (37).

## 3 Results

### 3.1 DNA concentration and quality of studied samples

After miRNA extraction of the samples, their concentration and quality were assessed with the NanoDrop 2000c spectrophotometer (Thermo Fisher Scientific). The results of miRNA concentration ranged from 6.95 to 4251.15 ng/μl, with the average value of 1220.45 ng/μl. The 260/280 ratios of the examined animals varied between 1.61 and 3.60, with the average value of 2.08. The 260/230 ratios ranged from 0.04 to 2.21 with a mean value of 1.33 (Supplementary Table 5).

### 3.2 MicroRNA expression profiles of canine lymphoma entities compared to control group ‘PBMC’

#### 3.2.1 DLBCL and PTCL (incl. Enteric TCL)

**Figure 1:**
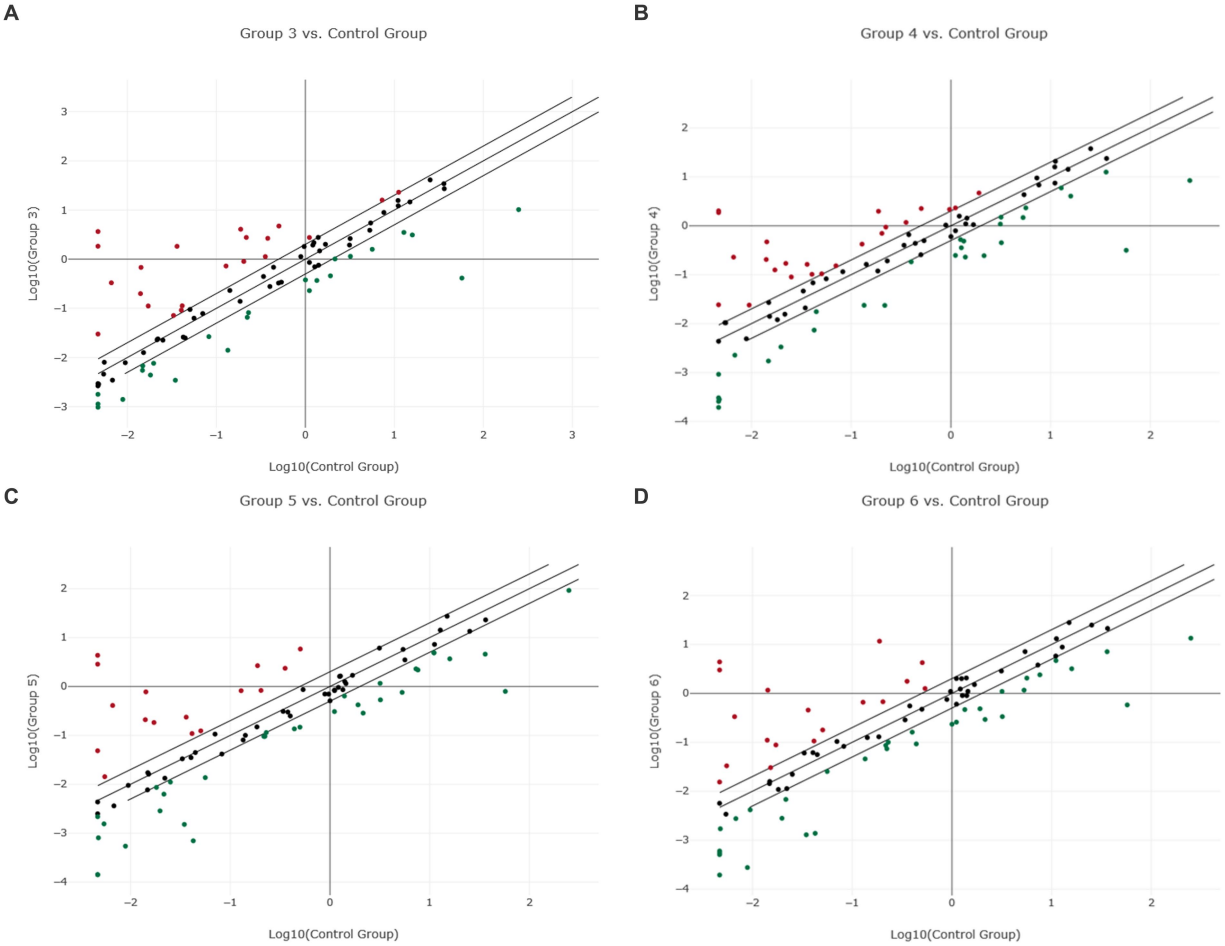
Scatter plot of normalized miRNA expression in four canine lymphoma entities compared to PBMC as control group. The center diagonal line indicates unchanged miRNA expression, while the outer diagonal lines indicate the Fold regulation threshold >2.0. MicroRNAs with data points beyond the outer lines in the upper left and lower right corners are up-regulated (red dots) or down-regulated (green dots), by more than the Fold regulation threshold in the y-axis Group relative to the x-axis Group. **(A)** Group 3 = DLBCL vs. Control Group = PBMC; **(B)** Group 4 = PTCL (incl. Enteric TCL) vs. Control Group = PBMC; **(C)** Group 5 = TZL vs. Control Group = PBMC; **(D)** Group 6 = MZL vs. Control Group = PBMC.

The predefined miRNAs (miR-16, miR-21, miR-22, miR-146a, and miR-350) had a stability factor below 1.5 and were used for normalization (Supplementary material “miRNA Expression Analysis Report 1,” page 10). In the 14 DLBCL patients, numerous miRNAs were dysregulated but only 24 miRNAs were significantly differentially expressed (Supplementary Figure 1; Supplementary Table 6). Out of these, 17 miRNAs were downregulated while seven miRNAs were upregulated (Figure 1A; Supplementary material “miRNA Expression Analysis Report 1” - Group 3). Five miRNAs showed Fold regulation (Fr) thresholds greater than ±20. The highest overexpression was found in miR-143 (Fr: 393.10, *p*-value: 0.012269), followed by miR-34a (Fr: 50.61, p-value: 0.006348), and miR-30a (Fr: 47.83, *p*-value: 0.001155). In contrast, downregulation was highest only in miR-223 (Fr: −138.82, *p-*value: 0.00) and miR-150 (Fr: −24.41, *p*-value: 0.000012; Table 1). The Fold regulation of miRNAs in PTCL and enteric TCL resulted in 25 significantly dysregulated miRNAs. Sixteen of those miRNAs were downregulated, while nine miRNAs were upregulated (Figure 1B; Supplementary material “miRNA Expression Analysis Report 1” - Group 4). Six of those miRNAs showed a higher Fold regulation threshold than ±20. Upregulations greater than 20 were found in four miRNAs which were the following: miR-143 (Fr: 432.19, *p*-value: 0.004948), miR-145 (Fr: 398.57, *p*-value: 0.01012), miR-214 (Fr: 34.58, *p*-value: 0.008617), and miR-30a (Fr: 32.97, *p*-value: 0.018914). Downregulations greater than −20 were found in two miRNAs which were miR-223 (Fr: −181.72, *p*-value: 0.000133) and miR-150 (Fr: −29.51, *p*-value: 0.000045; Table 1).

**Table 1:** Fold regulation of significantly dysregulated miRNAs in four canine lymphoma entities by using PBMC as control group.

miRNA ID	DLBCL	PTCL	TZL*	MZL*	miRNA ID	DLBCL	PTCL	TZL*	MZL*
cfa-let-7c	n.s.	n.s.	2.64	2.57	cfa-miR-136	n.s.	n.s.	n.s.	−2.27
cfa-let-7f	−2.80	n.s.	−2.01	−2.29	cfa-miR-143	393.10	432.19	612.46	643.49
cfa-miR-10a	n.s.	n.s.	2.60	6.01	cfa-miR-145	n.s.	398.57	928.49	940.67
cfa-miR-10b	n.s.	n.s.	10.38	3.31	cfa-miR-148a	n.s.	n.s.	n.s.	2.34
cfa-miR-15b	−4.83	n.s.	−3.61	−4.29	cfa-miR-149	n.s.	n.s.	n.s.	−2.47
cfa-miR-18a	2.49	2.09	n.s.	n.s.	cfa-miR-150	−24.41	−29.51	−2.70	−18.53
cfa-miR-20a	n.s.	n.s.	−2.24	n.s.	cfa-miR-151	−2.20	n.s.	n.s.	n.s.
cfa-miR-23a	−9.55	−5.68	n.s.	−2.94	cfa-miR-155	n.s.	n.s.	−6.90	−7.04
cfa-miR-24	−3.54	−2.43	n.s.	−2.75	cfa-miR-181a	−4.17	2.45	−4.53	−3.93
cfa-miR-25	n.s.	−2.12	−2.74	−2.90	cfa-miR-181b	n.s.	n.s.	−2.33	−3.01
cfa-miR-26b	−5.11	n.s.	−4.32	−4.99	cfa-miR-181c	−3.09	n.s.	n.s.	n.s.
cfa-miR-27a	−3.64	−2.15	n.s.	n.s.	cfa-miR-181d	n.s.	n.s.	−2.10	n.s.
cfa-miR-29a	n.s.	−3.58	n.s.	n.s.	cfa-miR-182	n.s.	n.s.	−2.25	n.s.
cfa-miR-29b	n.s.	−2.88	n.s.	n.s.	cfa-miR-183	n.s.	n.s.	−3.49	n.s.
cfa-miR-29c	n.s.	−2.38	n.s.	n.s.	cfa-miR-197	−2.80	−7.12	−6.01	−9.55
cfa-miR-30a	47.83	32.97	54.55	81.80	cfa-miR-199	n.s.	7.30	10.70	5.15
cfa-miR-30d	n.s.	−3.59	−6.98	−4.55	cfa-miR-200b	n.s.	n.s.	−5.90	−2.78
cfa-miR-31	n.s.	−9.21	−2.27	−2.51	cfa-miR-210	n.s.	n.s.	−3.39	n.s.
cfa-miR-34a	50.61	4.50	6.53	12.61	cfa-miR-214	n.s.	34.58	62.01	50.56
cfa-miR-92a	n.s.	−2.87	−7.86	−5.06	cfa-miR-218	n.s.	n.s.	n.s.	2.00
cfa-miR-93	n.s.	n.s.	−3.50	−3.18	cfa-miR-221	−2.64	n.s.	n.s.	−4.27
cfa-miR-99a	5.66	n.s.	6.39	5.14	cfa-miR-222	−3.65	−2.76	n.s.	−2.88
bta-miR-99b	n.s.	n.s.	14.90	7.87	cfa-miR-223	−138.82	−181.72	−72.21	−98.71
cfa-miR-101	n.s.	n.s.	2.45	3.54	cfa-miR-363	7.03	n.s.	n.s.	n.s.
cfa-miR-103	n.s.	n.s.	−2.27	−2.36	cfa-miR-378	n.s.	n.s.	−3.23	−4.73
cfa-miR-106a	2.19	n.s.	−3.17	n.s.	cfa-miR-423a	−2.12	−8.77	−7.52	−7.33
cfa-miR-107	n.s.	−2.18	n.s.	−2.47	cfa-miR-450a	−10.01	n.s.	−22.87	−26.89
cfa-miR-125a	n.s.	3.45	4.09	3.32	cfa-miR-450b	−6.30	n.s.	−16.36	−32.19
cfa-miR-125b	n.s.	4.47	11.58	8.43	cfa-miR-451	n.s.	n.s.	14.19	62.15
cfa-miR-126	n.s.	n.s.	6.66	4.98	cfa-miR-486	n.s.	n.s.	−2.19	n.s.
cfa-miR-128	n.s.	n.s.	−3.43	−3.17	cfa-miR-8865	n.s.	n.s.	−60.75	−30.77
cfa-miR-130b	n.s.	n.s.	−4.09	−2.22					

Fold regulation threshold > 2.0; *p*-value threshold < 0.05; *no calculated *p*-value; ID, identifier; DLBCL (Group 3), Diffuse Large B-cell lymphoma; PTCL (incl. Enteric T-cell lymphoma) (Group 4), Peripheral T-cell lymphoma; TZL (Group 5), T-zone lymphoma; MZL (Group 6), Marginal zone lymphoma; PBMC (Control Group), Peripheral blood mononuclear cells; cfa, Canis lupus familiaris; bta, Bos taurus; miR, microRNA; n.s., not significant.

**Figure 2:**
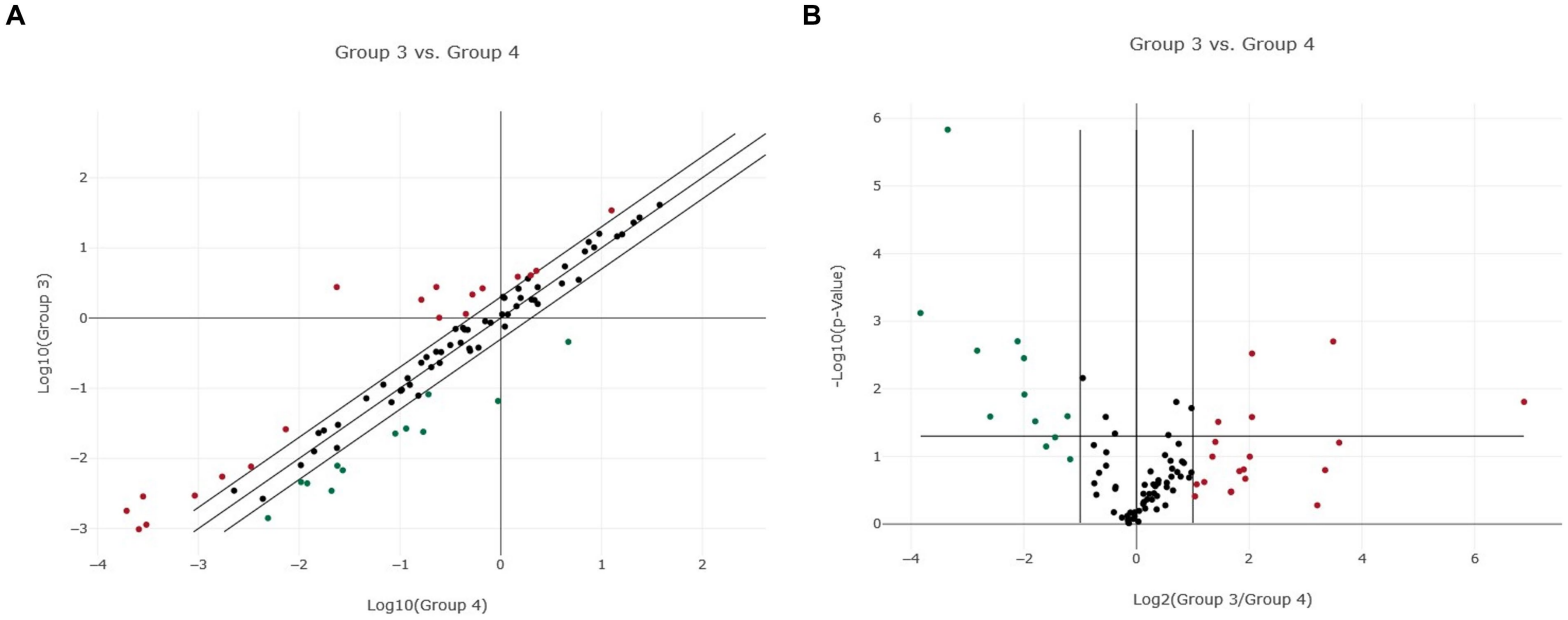
Normalized miRNA expression in two canine lymphoma entities. **(A)** Scatter plot of normalized miRNA expression comparing Group 3 (DLBCL) with Group 4 (PTCL, incl. Enteric TCL). The center diagonal line indicates unchanged miRNA expression, while the outer diagonal lines indicate the Fold regulation threshold >2.0. MicroRNAs with data points beyond the outer lines in the upper left and lower right corners are up-regulated (red dots) or down-regulated (green dots), by more than the Fold regulation threshold in the y-axis Group relative to the x-axis Group. **(B)** Volcano Plot illustrating significant miRNA expression changes when comparing Group 3 (DLBCL) with Group 4 (PTCL, incl. Enteric TCL). The center vertical line indicates unchanged miRNA expression, while the two outer vertical lines indicate the selected Fold regulation threshold. The horizontal line indicates the selected *p*-value threshold <0.05. MicroRNAs with data points in the far upper left and far upper right sections are down-regulated (green dots) or up-regulated (red dots) and meet the selected Fold regulation and p-value thresholds.

When comparing DLBCL and PTCL (incl. Enteric TCL), 24 out of 37 miRNAs were excluded from the analyzation due to lack of significance because of the analysis program (Figures 2A,B). Therefore, 13 miRNAs were excluded in DLBCL and 11 in PTCL. The remaining 13 miRNAs were significantly dysregulated in both entities (Table 1). As a matter of fact, one miRNA (miR-181a) was upregulated in PTCL and downregulation in DLBCL. Upregulations in both lymphoma entities were found in four miRNAs which were the following: miR-18a, miR-30a, miR-34a, and miR-143 (Table 1). Downregulations in both entities were detected in eight miRNAs (miR-23a, miR-24, miR-27a, miR-150, miR-197, miR-222, miR-223, and miR-423a; Table 1). In comparison, DLBCL and PTCL (incl. Enteric TCL) share 13 differentially expression miRNAs including four miRNAs that show potential specific expression profiles in these two entities (Fold regulations are given in parenthesis): miR-34a (DLBCL: 4.50 vs. PTCL: 50.61), miR-197 (DLBCL: −7.12 vs. PTCL: −2.80), miR-223 (DLBCL: −181.72 vs. PTCL: −138.82), and miR-423a (DLBCL: −8.77 vs. PTCL: −2.12; Table 1).

#### 3.2.2 TZL and MZL

As already mentioned, TZL and MZL samples only included two samples each and therefore no *p*-value calculation was possible. As precise statements are not justified due to the small sample size, the results must be taken into consideration cautiously. For TZL, data evaluation via the Qiagen Analysis platform revealed a total of 46 of the analyzed 89 miRNAs exhibiting a Fold regulation threshold >2.0 (Figure 1C; Supplementary material “miRNA Expression Analysis Report 1” - Group 5). Seven out of those 46 miRNAs exhibited Fold regulations greater than ±20, with four miRNAs being upregulated (miR-145, Fr: 928.49; miR-143, Fr: 612.46; miR-214, Fr: 62.01; miR-30a, Fr: 54.55). In contrast, three miRNAs were downregulated, including miR-223 (Fr: −72.21), miR-8865 (Fr: −60-75), and miR-450a (Fr: −22.87; Table 1). Analysis of the two MZL patients showed that in total 48 of the assessed miRNAs had a higher Fold regulation threshold than 2.0 (Figure 1D; Supplementary material “miRNA Expression Analysis Report 1” - Group 6). Eighteen miRNAs out of those 48 were upregulated, whereas 30 were downregulated. Nine miRNAs showed a Fold regulation threshold greater/lower than ±20. While miR-145 (Fr: 940.67), miR-143 (Fr: 643.49), miR-30a (Fr: 81.80), miR-451 (Fr: 62.15), and miR-214 (Fr: 50.56) were upregulated. In contrast, downregulations were found in miR-223 (Fr: −98.71), miR-450b (Fr: −32.19), miR-8865 (Fr: −30.77) and miR-450a (Fr: −26.89; Table 1). In comparison, TZL and MZL share 38 differentially expression miRNAs including nine miRNAs that show potential characteristic expression profiles in these two entities: miR-10b (TZL: 10.38 vs. MZL: 3.31), miR-30a (TZL: 54.55 vs. MZL: 81.8), miR-34a (TZL: 6.53 vs. MZL: 12.61), miR-99b (TZL: 14.90 vs. MZL: 7.87), miR-150 (TZL: −2.70 vs. MZL: −18.53), miR-199 (TZL: 10.70 vs. MZL: 5.15), miR-450b (TZL: −16.36 vs. MZL: −32.19), miR-451 (TZL: 14.19 vs. MZL: 62.15), and miR-8865 (TZL: −60.75 vs. MZL: −30.77; Table 1).

### 3.3 MicroRNA expression profiles of canine lymphoma entities compared to control group ‘lymph node’

#### 3.3.1 DLBCL and PTCL (incl. Enteric TCL)

**Figure 3:**
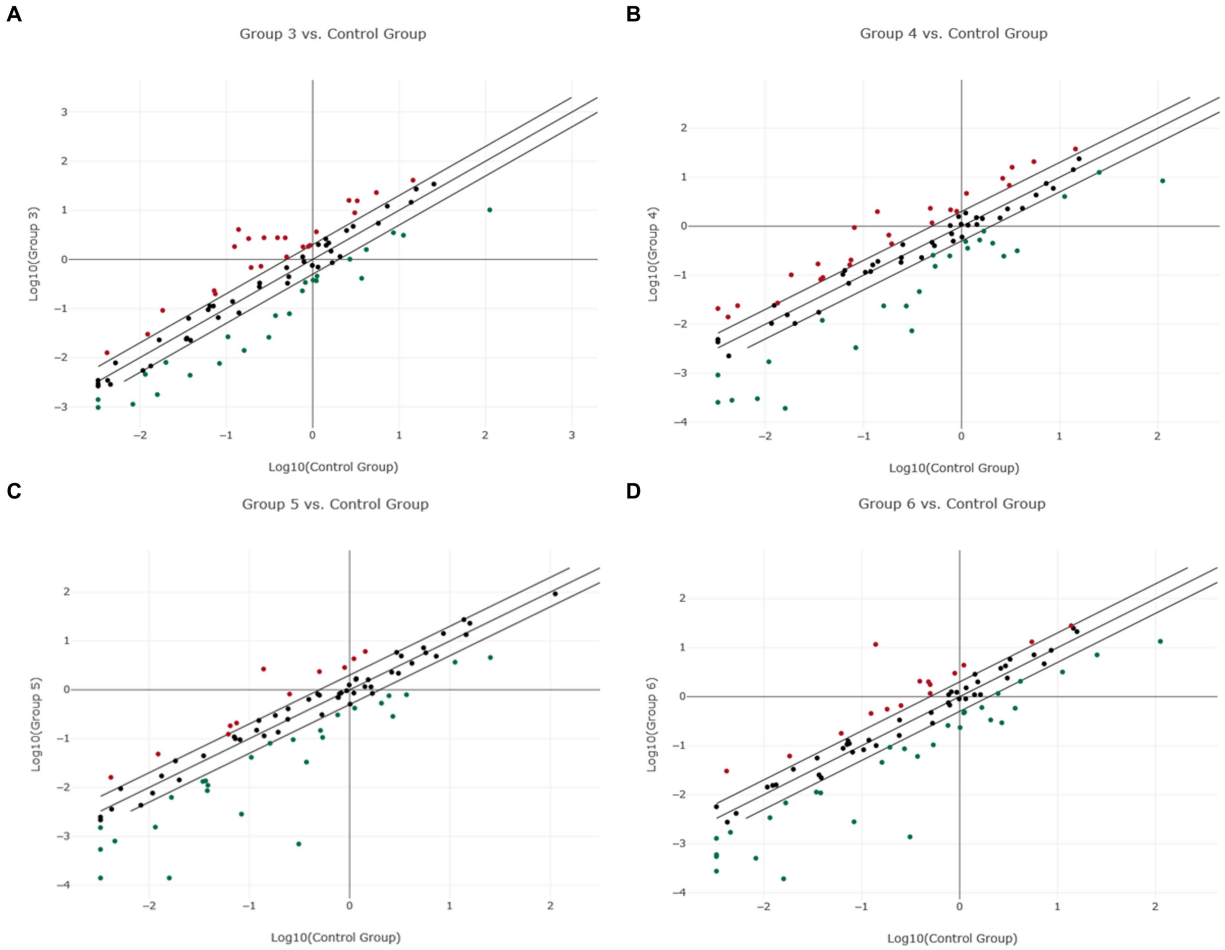
Scatter plot of normalized miRNA expression in four canine lymphoma entities compared to Lymph node (LN) as control group. The center diagonal line indicates unchanged miRNA expression, while the outer diagonal lines indicate the Fold regulation threshold >2.0. MicroRNAs with data points beyond the outer lines in the upper left and lower right corners are up-regulated (red dots) or down-regulated (green dots), by more than the Fold regulation threshold in the y-axis Group relative to the x-axis Group. **(A)** Group 3 = DLBCL vs. Control Group = LN; **(B)** Group 4 = PTCL (incl. Enteric TCL) vs. Control Group = LN; **(C)** Group 5 = TZL vs. Control Group = LN; **(D)** Group 6 = MZL vs. Control Group = LN.

Fold regulation of miRNAs in DLBCL showed numerous dysregulations, but only 28 miRNAs showed significant differences (Supplementary Figure 2; Table 7). Nineteen of those miRNAs were downregulated while 9 miRNAs were upregulated (Figure 3A; Supplementary material “miRNA Expression Analysis Report 2” - Group 3). Six miRNAs showed a Fold regulation threshold greater than ±10. The highest overexpression in fold regulations were found in miR-34a (Fr: 14.68, *p*-value: 0.008108) and miR-363 (Fr: 14.63, *p*-value: 0.008807). Downregulations greater than −10 were found in four miRNAs which were miR-8865 (Fr: −11.94, *p*-value: 0.003054), miR-23a (fold regulation: −11.43, *p*-value: 0.000000), miR-150 (Fr: −10.98, *p*-value: 0.018023) and miR-155 (Fr: −10.92, *p*-value: 0.000028; Table 2). Fold regulation of miRNAs in PTCL and enteric TCL resulted in 24 significant miRNA dysregulations. Fifteen of those miRNAs were downregulated while 9 miRNAs were overexpressed (Figure 3B; Supplementary material “miRNA Expression Analysis Report 2” - Group 4). Six of those miRNAs showed a Fold regulation below −10 (Table 2). Those downregulations were found in the following miRNAs: miR-8865 (Fr: −42.31, *p*-value: 0.048379), miR-155 (Fr: −25.11, *p*-value: 0.000169), miR-200b (Fr: −16.27, *p*-value: 0.031405), miR-150 (Fr: −13.28, *p*-value: 0.000105), miRNA-31 (Fr: −11.61, *p*-value: 0.00227), and miR-423a (Fr: - 11.00, *p*-value: 0.000563; Table 2).

**Table 2:** Fold regulation of significantly dysregulated miRNAs in four canine lymphoma entities by using Lymph node as control group.

miRNA ID	DLBCL	PTCL	TZL*	MZL*	miRNA ID	DLBCL	PTCL	TZL*	MZL*
cfa-miR-10a	−2.50	n.s.	n.s.	n.s.	cfa-miR-132	n.s.	n.s.	−2.58	−3.04
cfa-miR-10b	n.s.	n.s.	3.93	n.s.	cfa-miR-143	n.s.	n.s.	3.21	3.37
cfa-miR-15b	−3.31	n.s.	−2.47	−2.94	cfa-miR-145	n.s.	n.s.	3.93	3.99
cfa-miR-17	n.s.	n.s.	n.s.	3.35	cfa-miR-146a	−6.85	−3.53	−5.07	−5.18
cfa-miR-18a	5.74	4.83	n.s.	4.18	cfa-miR-148a	−2.41	n.s.	n.s.	n.s.
cfa-miR-19a	4.21	3.81	n.s.	2.41	cfa-miR-150	−10.98	−13.28	n.s.	−8.34
cfa-miR-19b	2.83	2.60	n.s.	n.s.	cfa-miR-155	−10.92	−25.11	−29.22	−29.79
cfa-miR-20a	4.77	4.89	n.s.	n.s.	cfa-miR-181a	n.s.	4.17	−2.66	−2.31
cfa-miR-21	n.s.	n.s.	n.s.	2.03	cfa-miR-181c	−3.93	n.s.	−2.52	n.s.
cfa-miR-23a	−11.43	−6.80	−2.00	−3.52	cfa-miR-181d	−8.66	n.s.	−4.41	−3.50
cfa-miR-24	−2.62	n.s.	n.s.	−2.03	cfa-miR-182	n.s.	n.s.	−3.47	n.s.
cfa-miR-26b	−3.61	−2.78	−3.05	−3.52	cfa-miR-183	n.s.	n.s.	−7.46	−3.41
cfa-miR-27a	−2.45	n.s.	n.s.	n.s.	cfa-miR-186	n.s.	−2.13	n.s.	−2.80
cfa-miR-29a	n.s.	−3.24	n.s.	n.s.	cfa-miR-197	n.s.	−4.61	−3.89	−6.19
cfa-miR-29b	n.s.	n.s.	4.25	n.s.	cfa-miR-199	n.s.	n.s.	2.84	n.s.
cfa-miR-29c	n.s.	−2.92	n.s.	n.s.	cfa-miR-200b	n.s.	−16.27	−5.70	−2.67
cfa-miR-30a	n.s.	n.s.	n.s.	2.33	cfa-miR-210	n.s.	n.s.	−3.47	n.s.
cfa-miR-30b	n.s.	n.s.	n.s.	n.s.	cfa-miR-218	n.s.	n.s.	3.87	7.28
cfa-miR-30d	n.s.	n.s.	−3.26	−2.12	cfa-miR-221	−2.66	n.s.	n.s.	−4.31
cfa-miR-31	n.s.	−11.61	−2.86	−3.16	cfa-miR-222	−2.98	−2.26	n.s.	−2.36
cfa-miR-34a	14.68	n.s.	n.s.	3.66	cfa-miR-223	−8.93	n.s.	−4.64	−6.35
cfa-miR-92a	n.s.	−2.01	−5.51	−3.55	cfa-miR-350	3.19	2.25	n.s.	n.s.
cfa-miR-93	2.91	2.23	n.s.	n.s.	cfa-miR-363	14.63	n.s.	n.s.	3.06
cfa-miR-99a	n.s.	n.s.	3.25	2.62	cfa-miR-378	n.s.	n.s.	n.s.	−2.09
bta-miR-99b	n.s.	n.s.	2.80	n.s.	cfa-miR-423a	−2.66	−11.00	−9.42	−9.18
cfa-miR-101	n.s.	n.s.	2.00	2.90	cfa-miR-450a	n.s.	n.s.	−2.17	−2.55
cfa-miR-106a	6.07	3.61	n.s.	n.s.	cfa-miR-450b	−2.34	n.s.	−6.05	−11.91
cfa-miR-106b	n.s.	2.80	n.s.	n.s.	cfa-miR-451	n.s.	n.s.	19.20	84.07
cfa-miR-126	n.s.	n.s.	4.73	3.53	cfa-miR-486	n.s.	n.s.	n.s.	5.27
cfa-miR-128	n.s.	n.s.	−2.66	−2.46	cfa-miR-671	−5.17	n.s.	−11.18	−6.14
cfa-miR-130b	n.s.	n.s.	−2.68	n.s.	cfa-miR-8865	−11.94	−42.31	−444.91	−225.39

Fold regulation threshold > 2.0; *p*-value threshold < 0.05; *no calculated *p*-value; ID = identifier; DLBCL (Group 3) = Diffuse Large B-cell lymphoma; PTCL (incl. Enteric T-cell lymphoma) (Group 4) = Peripheral T-cell lymphoma; TZL (Group 5) = T-zone lymphoma; MZL (Group 6) = Marginal zone lymphoma; PBMC (Control Group), Peripheral blood mononuclear cells; cfa, Canis lupus familiaris; bta, Bos taurus; miR, microRNA; n.s., not significant.

When comparing DLBCL and PTCL (incl. Enteric TCL), 22 out of 37 miRNAs were excluded from the analyzation due to lack of significance because of the analysis program (Figures 2A,B). Consequently, nine miRNAs were excluded in DLBCL and 13 in PTCL. However, these entities share significantly dysregulated miRNAs which were either up-or downregulated in both groups (Table 2). Upregulations in both lymphoma entities were found in seven miRNAs: miR-18a, miR-19a, miR-19b, miR-20a, miR-93, miR-106a, and miR-350. Downregulations in both entities were shown in eight miRNAs including miR-23a, miR-26b, miR-146a, miR-150, miR-155, miR-222, miR-423a, and miR-8865 (Table 2). In comparison, DLBCL and PTCL (incl. Enteric TCL) share 15 differentially expression miRNAs including four miRNAs that show potential specific expression profiles in these two entities (Fold regulations are given in parenthesis): miR-23a (DLBCL: −11.43 vs. PTCL: 6.80), miR-155 (DLBCL: −10.92 vs. PTCL: −25.11), miR-423a (DLBCL: −2.66 vs. PTCL: −11.00), and miR-8865 (DLBCL: −11.94 vs. PTCL: −42.31; Table 2).

#### 3.3.2 TZL and MZL

As already mentioned, TZL and MZL samples included only two samples, therefore, no *p*-value calculation was possible. As precise statements are not justified due to the small sample size, the presented results must be taken into consideration cautiously. For TZL, data evaluation via the Qiagen Analysis platform revealed a total of 36 of the analyzed miRNAs exceeding the Fold regulation threshold of 2.0 including 11 upregulated and 25 downregulated miRNAs (Figure 3C; Supplementary material “miRNA Expression Analysis Report 2” - Group 5). Among these 36, four miRNAs showed Fold regulation values greater/lower than ±10; whereas upregulation was found in one miRNA, namely miR-451 (Fr: 19.20) On the other hand, three miRNAs were downregulated, including miR-155 (Fr: −29.22), miR-671 (Fr: −11.19), and miR-8865 (Fr: −444.91; Table 2). Analysis of the two MZL patients showed that in total 42 of the assessed miRNAs had a higher Fold regulation threshold than 2.0 (Figure 3D; Supplementary material “miRNA Expression Analysis Report 2” - Group 6). Fifteen miRNAs out of those 42 were upregulated, whereas 27 were downregulated. Four miRNAs showed a Fold regulation threshold greater/lower than ±10. While miR-451 (Fr: 84.07) was upregulated, downregulations were found in miR-155 (Fr: −29.79), miR-450b (Fr: −11.91) and miR-8865 (Fr: −225.39; Table 2).

### 3.4 Influence of selected control group on microRNA expression data analysis

In this section, the lymphoma entities DLBCL and PTCL (incl. Enteric TCL) will be compared to the two control groups to evaluate data consistency and to describe potential differences (Table 3; Supplementary Table 8). In DLBCL, 16 miRNAs showed Fold regulations >2.0 when compared to both control groups, albeit 12 and 8 not significantly dysregulated miRNAs had to be excluded in comparison to PBMC or lymph node (LN), respectively (Table 3). Out of these 16 dysregulated miRNAs, miR-18a, miR-34a, miR-106a, and miR-363 were upregulated in both groups (PBMC and LN; Supplementary Figure 3). Downregulations were observed in 12 miRNAs: miR-15b, miR-23a, miR-24, miR-26b, miR-27a, miR-150, miR-181c, miR-221, miR-222, miR-223, miR-423a, and miR-450b (Table 3). For most miRNAs, Fold regulations were higher in PBMCs than in lymph node. Interestingly, all seven significantly dysregulated miRNAs exhibited higher Fold regulations in physiological lymph nodes compared to PBMCs: miR-18a, miR-23a, miR-106a, miR-181c, miR-221, miR-363, and miR-423a. MiRNA-18a, miRNA-106a, and miRNA-363 were upregulated, whereas miRNA-23a, miRNA-181c, miRNA-221, and miRNA-423a were downregulated (Table 3). In PTCL, for 11 miRNAs Fold regulations >2.0 were found when compared to both control groups, although 13 and 15 not significantly dysregulated miRNAs had to be excluded in comparison to PBMC or LN, respectively (Table 3). Out of these 11 dysregulated miRNAs, miR-18a and miR-181a were upregulated in both groups (PBMC and LN; Supplementary Figure 4). Downregulations were found for nine miRNAs: miR-23a, miR-29a, miR-29c, miR-31, miR-92a, miR-150, miR-197, miR-222, and miR-423a. As in DLBCL, six out of these miRNAs revealed higher Fold regulations with LN than in comparison to PBMC, including miR-18a, miR-181a, miR-23a, miR-29c, miR-31, and miR-423a (Table 3). Finally, three significantly dysregulated miRNAs were shared between by both entities in comparisons with PBMC and LN as control groups (Fold regulations are given in parenthesis): miR-18a (DLBCL/PBMC: 2.49; DLBCL/LN: 5.74; PTCL/PBMC: 2.09; PTCL/LN: 4.83), miR-23a (DLBCL/PBMC: -9.55; DLBCL/LN: -11.43; PTCL/PBMC: -5.68; PTCL/LN: −6.80), and miR-423a (DLBCL/PBMC: -2.12; DLBCL/LN: -2.66; PTCL/PBMC: -8.77; PTCL/LN: −11.00; Table 3).

**Table 3:** DLBCL and PTCL (incl. Enteric TCL) miRNA fold regulations compared between the two control groups.

	DLBCL		PTCL	
miRNA ID	PBMC	LN	PBMC	LN
cfa-miR-15b	−4.83	−3.31	n.m.	n.m.
**cfa-miR-18a**	**2.49**	**5.74**	**2.09**	**4.83**
**cfa-miR-23a**	**−9.55**	**−11.43**	**−5.68**	**−6.80**
cfa-miR-24	−3.54	−2.62	n.m.	n.m.
cfa-miR-26b	−5.11	−3.61	n.m.	n.m.
cfa-miR-27a	−3.64	−2.45	n.m.	n.m.
cfa-miR-29a	n.m.	n.m.	−3.58	−3.24
**cfa-miR-29c**	n.m.	n.m.	**−2.38**	**−2.92**
**cfa-miR-31**	n.m.	n.m.	**−9.21**	**−11.61**
cfa-miR-34a	50.61	14.68	n.m.	n.m.
cfa-miR-92a	n.m.	n.m.	−2.87	−2.01
**cfa-miR-106a**	**2.19**	**6.07**	n.m.	n.m.
cfa-miR-150	−24.41	−10.98	−29.51	−13.28
**cfa-miR-181a**	n.m.	n.m.	**2.45**	**4.17**
**cfa-miR-181c**	**−3.09**	**−3.93**	n.m.	n.m.
cfa-miR-197	n.m.	n.m.	−7.12	−4.61
**cfa-miR-221**	**−2.64**	**−2.66**	n.m.	n.m.
cfa-miR-222	−3.65	−2.98	−2.76	−2.26
cfa-miR-223	−138.82	−8.93	n.m.	n.m.
**cfa-miR-363**	**7.03**	**14.63**	n.m.	n.m.
**cfa-miR-423a**	**−2.12**	**−2.66**	**−8.77**	**−11.00**
cfa-miR-450b	−6.30	−2.34	n.m.	n.m.

MicroRNAs marked in bold exhibited higher fold regulations when using ‘control group’ LN for data analysis compared to ‘control group’ PBMC. Fold regulation threshold > 2.0; *p*-value threshold < 0.05; ID, identifier; DLBCL, Diffuse Large B-cell lymphoma; PTCL (incl. Enteric T-cell lymphoma), Peripheral T-cell lymphoma; PBMC, Peripheral blood mononuclear cells; LN, lymph node; cfa, Canis lupus familiaris; miR, microRNA; n.m., no match.

## 4 Discussion

### 4.1 Differentially expressed microRNAs in canine lymphoma entities

In this exploratory study, canine lymphoma sample material was compared to two different control groups, PBMC and physiological lymph node material. When comparing the different lymphoma entities to the control groups, there were bigger differences in dysregulations using PBMC as control group than to physiological lymph nodes. Not many miRNAs showed significant differences between PBMC and DLBCL. In contrast, in the six PTCL (incl. Enteric TCL) patient samples, a higher number of miRNAs were significantly differentially expressed when using the same control group. In a comparable study, malignant samples were also compared to the two different control groups PBMC and lymph nodes, which also showed that the use of more than one control group is important for the analysis of dysregulated miRNAs in canine lymphomas (1).

MicroRNA-350 showed upregulation in DLBCL in comparison to lymph node material, being consistent with findings in previous studies (5). By downregulating Phosphoinositide-3-Kinase Regulatory Subunit 3 (PIK3R3), miRNA-350 promotes apoptosis. However, upregulations could be explained through its properties of inactivation of immune cells around tumors (5). In agreement with current canine literature, miRNA-155 was downregulated in both lymphoma entities when using physiological lymph nodes as control group. Since miRNA-155 activates the AKT serine/threonine kinase signaling pathway, downregulation leads to a higher proliferation as well as a higher risk for developing a more aggressive tumor (4, 7, 38). Downregulation of miRNA-23a and miRNA-26b in DLBCL and PTCL is supported by previous canine lymphoma studies (3, 39, 40). MicroRNA-23a was downregulated in both lymphoma entities when comparing them to both control groups, while for miRNA-26b this was only the case by using physiological lymph nodes as control group. To current knowledge, miRNA-23a targets two and miRNAs-26b five tumor suppressor genes, respectively. Here, the detected downregulation of these miRNAs promotes cell proliferation (3, 39, 40). Likewise in the current study, miRNA-106a was upregulated in canine DLBCL, when compared to both control groups, thus promoting uncontrolled lymphoid proliferation, cellular growth, and apoptosis inhibition (41). This axis is also regulated by miRNA-31 which was found to be downregulated in PTCL in comparison to both control groups. This finding contrasts with data being obtained during a recently conducted miRNome expression analysis in six canine DLBCL patient samples (41).

The oncogenic polycistronic cluster miRNA-17/92 was represented by five miRNAs: miRNA-18a, miRNA-19a, miRNA-19b, miRNA-20a, miRNA-92a. Except the latter one, miRNAs were upregulated in the lymphoma entities, DLBCL and PTCL when compared to lymph nodes as control group. Only miRNA-18a was also significantly expressed in comparison to PBMC, as already being described in previous studies (1, 3, 4). Upregulation of the miRNA-17/92 cluster seems to reflect the functional roles of its miRNA members as potential oncogenes in proliferation, tumor initiation and metastasis while also working as an inhibitor for apoptosis (4, 7, 41). The miRNA-181 family or to be more precise miRNA-181a (compared to PBMC), miRNA-181c (compared to both control groups), and miRNA181d (compared to physiological lymph nodes) were all downregulated in DLBCL as already been shown previously (1, 3). Those miRNAs play an important role in B- and T-cell development as they are part of the thymic differentiation, positive as well as negative selection (3). However, miRNA-181 seems to be differentially expressed in B- and T-cell lymphoma as miRNA-181a was downregulated in DLBCL while upregulated in PTCL when compared to both control groups. The overexpression of miRNA-181a has already been shown in other studies focusing on canine lymphoma (3, 7, 22). In human, miRNA-181a affects γδ T cell differentiation and depending on the cellular context it can act as a tumor suppressor (B-cell lymphoma) but can also play a role in oncogenesis (T-cell lymphoma) (22, 42, 43).

### 4.2 Comparative microRNA expression in human and canine lymphoma

MicroRNA-25 which was significantly downregulated in PTCL using PBMC as control group, reflecting same trends found in literature by playing an important role in regulating large tumor suppressor kinase 2 (LATS2) (2, 4, 44). In human, miRNA-25 also regulates other tumor-related genes, such as p53 and E-cadherin (2). Downregulation of miRNA-25 leads to proliferation, tumor initiation, metastasis, cell migration, invasion, and apoptosis by different pathways (4, 44). As found in this study, miRNA-150 was downregulated in DLBCL and PTCL (incl. Enteric TCL) compared to PBMC and physiological lymph node samples. It has been shown that miRNA-150 is expressed in murine mature resting B- and T-cells, leading to the assumption that its downregulation mirrors the reduction of lymphocytes in neoplastic lymph nodes (3, 41). In human, shorter survival time and negative therapeutic response might by evidenced by miRNA-150 downregulation (3, 7). Downregulation of miRNA-151 in DLBCL when using PBMC as control group was also found in human lymphoma in the regulation of target genes being associated with tumor cells such as the neurotrophic tyrosine receptor kinase 2 (NTRK2) gene (5). When comparing PTCL (incl. Enteric TCL) to PBMC and lymph nodes, three canine miRNAs showed differences in dysregulation between the current study and published data (4, 45). These miRNAs were miRNA-29a (both control groups), miRNA-29b (PBMC as control group), and miRNA-29c (both control groups). Here, all three miRNAs were downregulated, while they usually tend to be upregulated in human study material, pointing towards different regulatory functions of these miRNAs in dogs, needing further investigations (4, 41, 45). In this study, miRNA-34a showed upregulation in both canine lymphoma entities (DLBCL and PTCL) when using both control groups, while in human, it has been shown that miRNA-34a works as tumor suppressor and is therefore downregulated (3, 7, 11). In canine lymphoma, previous studies confirmed upregulation of miRNA-34a of which it is known to target four genes being involved in cell migration thus acting as oncogene (3, 4, 7). The results for miRNA-143 being obtained in DLBCL and PTCL showed major discrepancies to previous studies. In mice, miRNA-143 was found to be downregulated in radiation induced thymic lymphoma (46). Two genes are influenced by miRNA-143, namely programmed death-ligand 1 (B7H1) and the B-cell lymphoma apoptosis regulator (Bcl-2), hence downregulation of miRNA-143 influences tumor B-cell proliferation (4, 46). Since the role of miRNA-143 in dogs has not yet been described, upregulation of miRNA-143 in canine DLBCL and PTCL could point towards an alternative role of miRNA-143 in dogs by acting as an oncogene in T- and B-cell lymphoma.

### 4.3 Novel described dysregulated microRNAs in canine lymphoma

At time of data analyses for the presented study, no literature evidence was available for some of the differentially expressed miRNAs under investigation. As an example, miRNA-15b, miRNA-24, miRNA-27a, miRNA-221, miRNA-223, and miRNA-450b were found to be downregulated in DLBCL in comparison to both control groups. Notably, upregulation of miRNA-363 was detected in DLBCL when compared to both control groups, PBMC and healthy lymph nodes. Together with miRNA-106a, miRNA-363 builds the oncogenic cluster miRNA-106a/363 (miRNA-106a and miRNA-92a), the paralogue to the miRNA-17/92 cluster. Additionally, miRNA-222 and miRNA-423a showed downregulations in both canine lymphoma entities, whereas miRNA-197 was downregulated only in PTCL when compared to both control groups. However, future studies will require a higher number of patients and sample number in each control group to better interpret these miRNA results.

## 5 Conclusion

In summary, numerous miRNAs were amplified in this exploratory project and exhibited similar results compared to other studies. Some miRNAs could be potential biomarkers in different lymphoma entities, such as the differentially expressed miRNA-181a in the investigated canine T- and B-cell lymphoma patients. Furthermore, miRNA-34a as well as miRNA-150 were significantly dysregulated in comparison to the control groups. The following miRNAs were suggested by the miRNA PCR Data Analysis Software (Qiagen) for further validation as potential biomarkers for canine DLBCL: miR-15b, miR-18a, miR-26b, miR-34a, miR-150, miR-181c, miR-223, miR-363, miR-423a, and miR-450b. However, to validate the diagnostic utility of these potential biomarkers, further studies are needed to confirm the miRNAs identified in this study as well as those, for which no data were available in the current literature. Hence, it would be advisable to work with a larger, prospective cohort of patients in all lymphoma entities and to continue the use of multiple control groups with a higher number of physiological patient material. It is of utmost importance to conduct more studies with larger sample sizes to assign differential biomarkers to canine lymphoma entities to primarily distinguish DLBCL from other lymphoma subtypes and reactive lymph nodes.

## Data availability statement

The original contributions presented in the study are included in the article/Supplementary material, further inquiries can be directed to the corresponding author/s.

## Ethics statement

Ethical approval was not required for the studies involving animals in accordance with the local legislation and institutional requirements because remnants of biopsy material of canine lymphoma cases served as study material together with lymph node material from eight dogs, being euthanized for reasons other than hematopoietic neoplasia. Their remains were left by the owners for disposal. Written informed consent was obtained from the owners for the participation of their animals in this study.

## Author contributions

SH: Conceptualization, Investigation, Project administration, Visualization, Writing – original draft. JS: Formal analysis, Methodology, Writing – original draft. OŠ: Methodology, Resources, Writing – review & editing. SB: Methodology, Resources, Writing – review & editing. MH: Resources, Writing – review & editing, Methodology. IS: Project administration, Resources, Supervision, Writing – review & editing. BR: Resources, Writing – review & editing, Project administration, Supervision.
