# Supplementary material for: Exploratory screening for micro-RNA biomarkers in canine multicentric lymphoma
Source: Front Vet Sci. 2024 May 17;11:1379146. doi: 10.3389/fvets.2024.1379146 (PMC11141397; doi:10.3389/fvets.2024.1379146)
Supplement: Supplementary file 1 [file Data_Sheet_1.pdf]

Supplementary Material

Exploratory Screening for Micro-RNA Biomarkers in Canine Multicentric Lymphoma

Sabine E. Hammer[1,*], Julia Sprung[1,†], Ondřej Škor[2], Stefanie Burger[3], Martin Hofer[4], Ilse Schwendenwein[5], Barbara C. Rütgen[5]

[1]Immunology, Department of Biological Sciences and Pathobiology, University of Veterinary Medicine Vienna, Vienna, Austria

[2]Laboklin GMBH & CO.KG, Bad Kissingen, Germany

[3]VetBioBank, VetCore, University of Veterinary Medicine Vienna, Vienna, Austria

[4]Genomics Core Facility, VetCore, University of Veterinary Medicine Vienna, Vienna, Austria

[5]Clinical Pathology, Department of Biological Sciences and Pathobiology, University of Veterinary Medicine Vienna, Vienna, Austria

*** Correspondence:**
Sabine E. Hammer
sabine.hammer@vetmeduni.ac.at

[†] These authors contributed equally to this work.

4 Supplementary Figures and Tables

4.1 Supplementary Figures

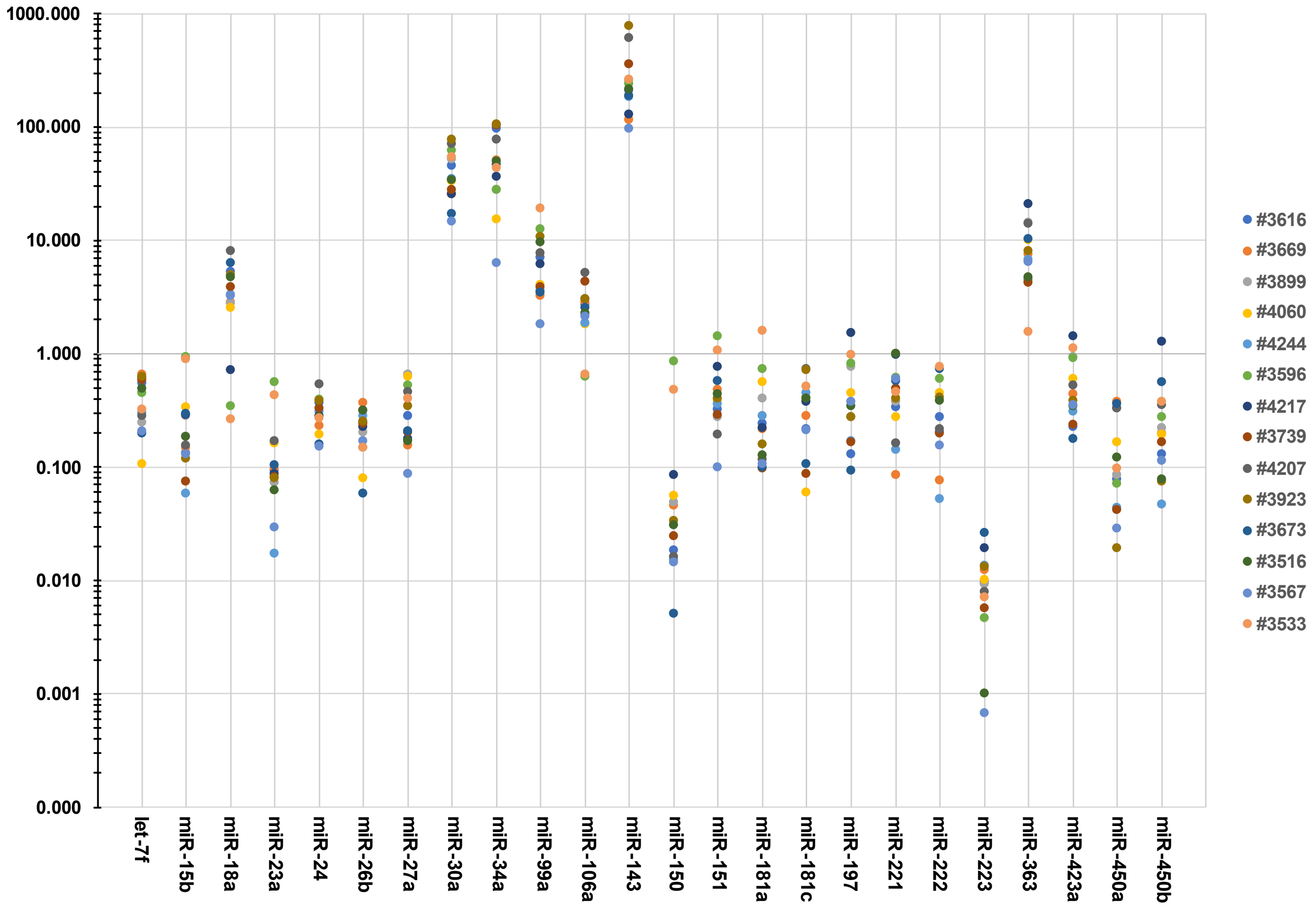

Supplementary Figure 1. Significantly dysregulated microRNAs in canine Diffuse Large B-cell lymphoma (DLBCL). Individual Fold change values [$2^{(-\Delta\Delta Ct)}$] of 14 canine DLBCL patients compared to PBMC as control group are drawn on a logarithmic scale. miR = microRNA. For details, please refer to Supplementary Table 5.

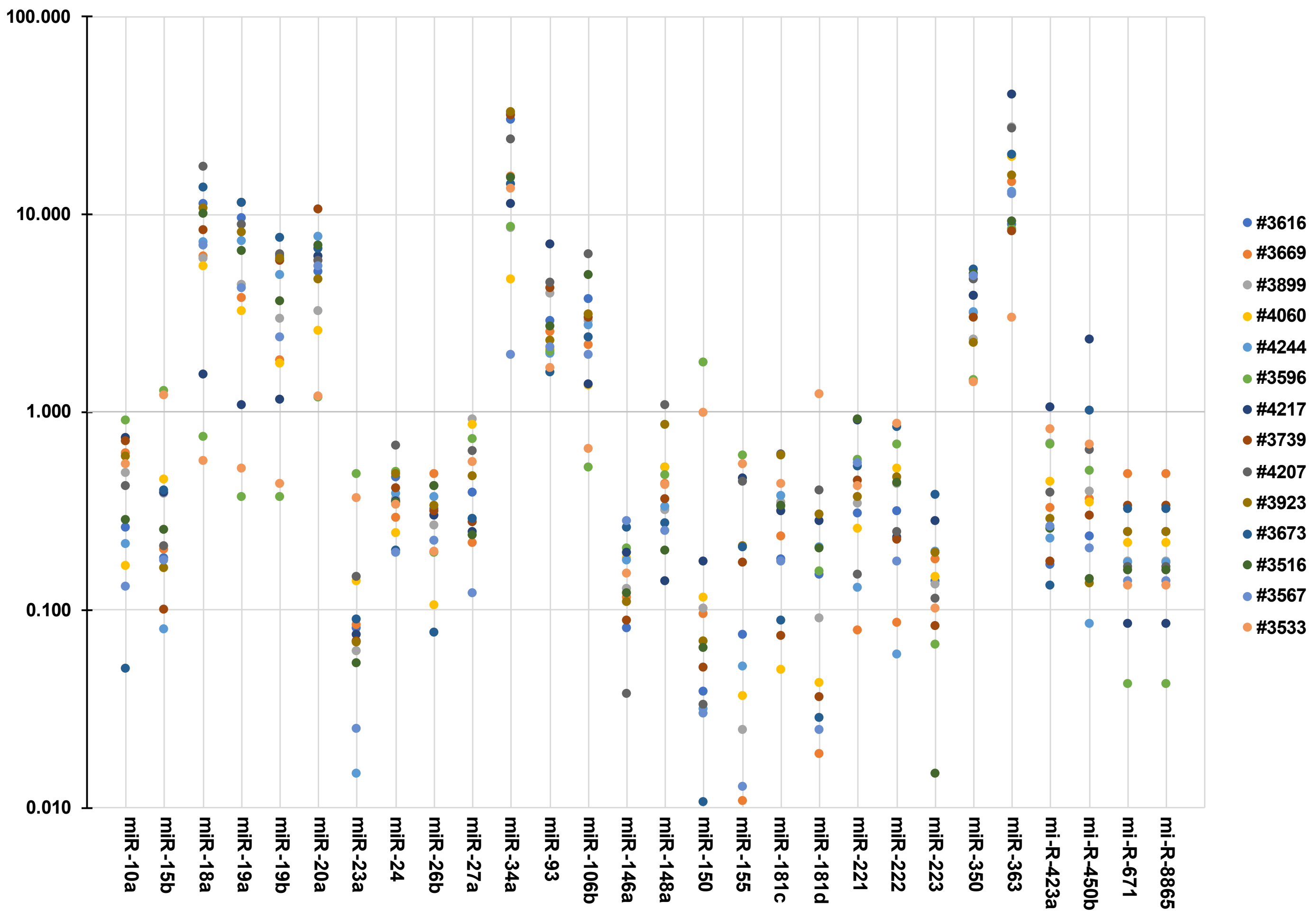

Supplementary Figure 2. Significantly dysregulated microRNAs in canine Diffuse Large B-cell lymphoma (DLBCL). Individual Fold change values [$2^{(-\Delta\Delta Ct)}$] of 14 canine DLBCL patients compared to lymph node as control group are drawn on a logarithmic scale. miR = microRNA. For details, please refer to Supplementary Table 6.

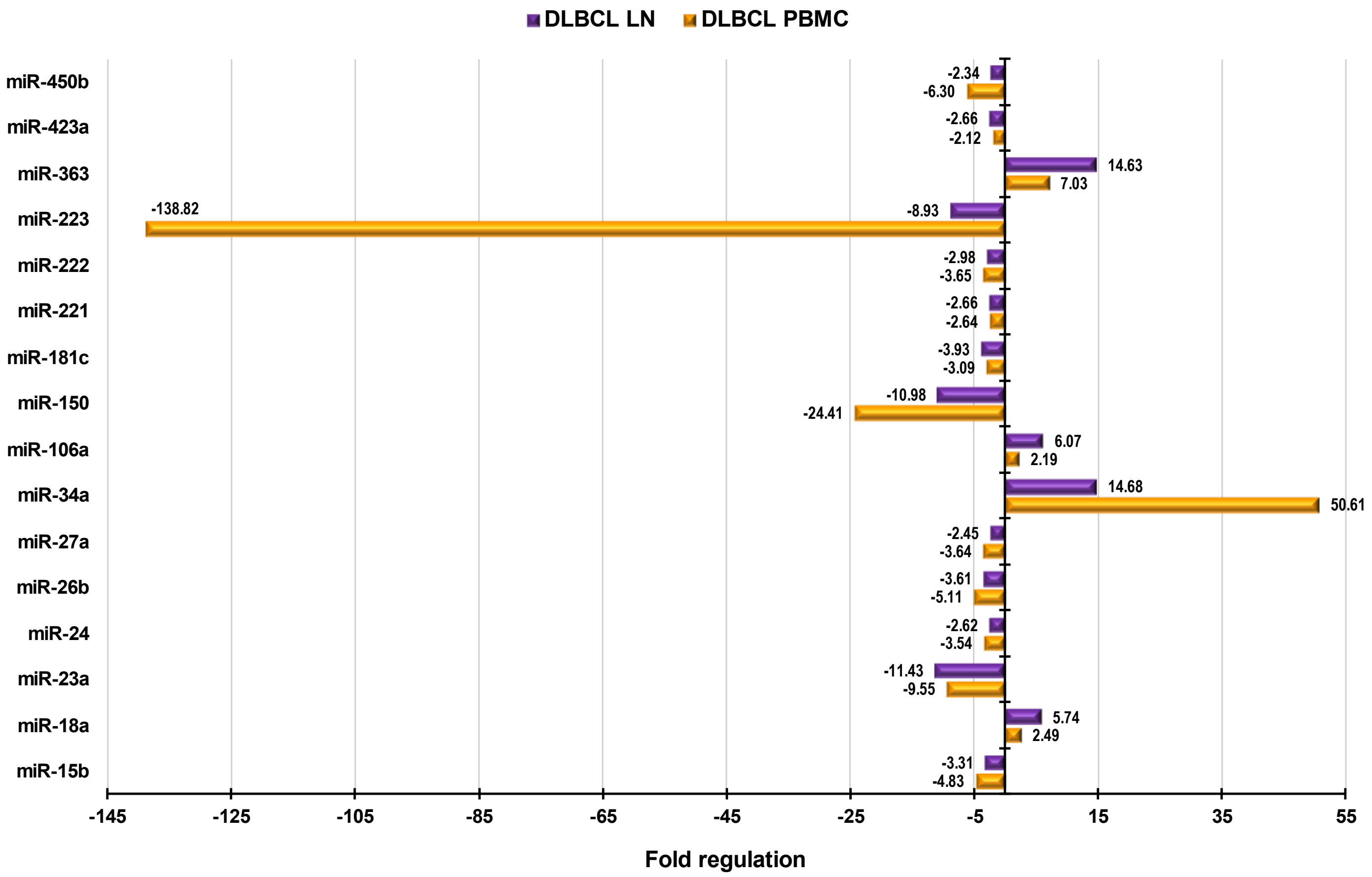

Supplementary Figure 3. Shared microRNAs in canine DLBCL being significantly dysregulated when using PBMC and LN as control groups. Fold regulation threshold > 2.0; p-value threshold < 0.05; DLBCL = Diffuse Large B-cell lymphoma; PBMC = Peripheral blood mononuclear cells; LN = lymph node; miR = microRNA. For details, please refer to Table 3.

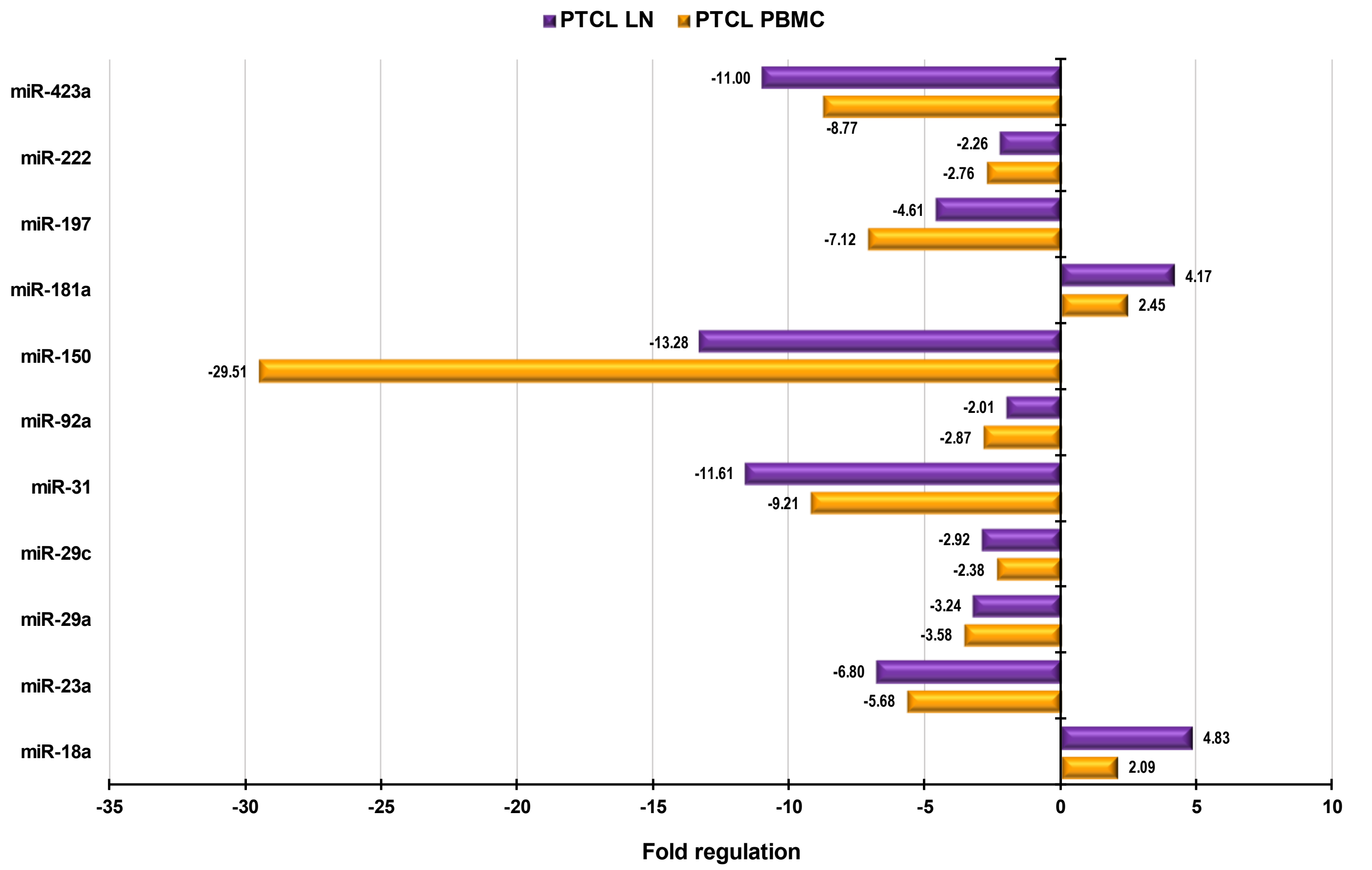

Supplementary Figure 4. Shared microRNAs in canine PTCL (incl. enteric TCL) being significantly dysregulated when using PBMC and LN as control groups. Fold regulation threshold > 2.0; p-value threshold < 0.05; PTCL (incl. enteric T-cell lymphoma) = Peripheral T-cell lymphoma; PBMC = Peripheral blood mononuclear cells; LN = lymph node; miR = microRNA. For details, please refer to Table 3.

4.2 Supplementary Tables

Supplementary Table 1. Canine lymphoma patient list.

Case ID Pathology	TIS-Animal Number	Analysis Group	Vet Biobank ID	Date	Breed	Sex	Age (years)	Final diagnosis[a]	Tissue for histological examination[b]	Sample type
5539-18	T/2010/012702	Group 5	3442	05.03.2018	Bullterrier	f	8	T-zone lymphoma	lymph node	surgical
5589-18	T/2018/002423	Group 4	3447	15.03.2018	mixed	m	8	Peripheral T-cell lymphoma	retropharyngeal area, lymph node - both PTCL	biopsy
6420-18	T/2018/007690	Group 3	3516	27.06.2018	mixed	f	11	Diffuse large B-cell lymphoma	lymph node	surgical
6573-18	T/2018/009086	Group 3	3533	24.07.2018	Australian Shepherd	f	9	Diffuse large B-cell lymphoma	lymph node	surgical
6682-18	T/2018/009910	Group 4	3558	13.08.2018	Pug	m	9	Peripheral T-cell lymphoma	lymph node	surgical
6782-18	T/2016/004369	Group 3	3567	22.08.2018	Border Collie	m	11	Diffuse large B-cell lymphoma	lymph node	surgical
6842-18	T/2018/010950	Group 3	3596	04.09.2018	mixed;	f	11	Diffuse large B-cell lymphoma	lymph node	surgical
7046-18	T/2018/012249	Group 3	3616	04.10.2018	Rottweiler	w	5	Diffuse large B-cell lymphoma	lymph node	surgical
7320-18	T/2018/013444	Group 3	3669	15.11.2018	mixed	f	5	Diffuse large B-cell lymphoma	lymph node	surgical
7367-18	T/2012/007105	Group 3	3673	14.11.2018	mixed	fs	10	Diffuse large B-cell lymphoma	lymph node	biopsy
7513-18	T/2018/014583	Group 5	3712	05.12.2018	Flat Coated Retriver	f	8	T-zone lymphoma	lymph node	surgical
7514-18	T/2018/014327	Group 6	3715	05.12.2018	Foxterrier	m	11	Marginal zone lymphoma	spleen	surgical
7598-18	T/2011/007444	Group 3	3739	18.12.2018	Rottweiler	f	8	Diffuse large B-cell lymphoma	lymph node	surgical

5078-19	T/2019/000298	Group 4	3757	11.01.2019	Cocker Spaniel	m	10	Peripheral T-cell lymphoma	lymph node	surgical
5134-19	T/2019/000572	Group 4	3775	22.02.2019	mixed	m	11	Enteric T-cell lymphoma	intestine, lymph node, liver - all enteric T-cell lymphoma	surgical
5280-19	T/2019/001380	Group 4	3840	08.02.2019	Australian Shepherd	m	5	Peripheral T-cell lymphoma	lymph node	surgical
5351-19	T/2019/001722	Group 6	3867	26.02.2019	Magyar Vizsla	m	8	Marginal zone lymphoma	lymph node	surgical
5547-19	T/2019/002782	Group 3	3923	27.03.2019	Rottweiler	f	7	Diffuse large B-cell lymphoma	lymph node	surgical
5467-19	T/2019/002426	Group 3	3899	12.03.2019	sighthound mixed	f	8	Diffuse large B-cell lymphoma	lymph node	surgical
6074-19	T/2019/005869	Group 4	3998	06.06.2019	Malinois	f	9	Peripheral T-cell lymphoma	Lymph node	surgical
A/6337/19	T/2019/006051	Group 3	4060	16.07.2019	American Staffordshire Terrier	m	12	Diffuse large B-cell lymphoma	Lymph node	surgical
K/7212/19	T/2014/001869	Group 3	4207	29.10.2019	mixed	m	12	Diffuse large B-cell lymphoma	Lymph node	surgical
T/7227/19	T/2019/011974	Group 3	4217	04.11.2019	Magyar Vizsla	f	8	Diffuse large B-cell lymphoma	Lymph node	surgical
R/7361/19	T/2019/012400	Group 3	4244	12.11.2019	Saluki	m	6	Diffuse large B-cell lymphoma	Lymph node	surgical

[a]Histology and Immunohistochemistry;

[b]e.g., lymph node, spleen, small intestine; TIS = Tierinformationssystem (Patient information system); f = female; m = male.

Supplementary Table 2. List of canine non-neoplastic samples.

Sample ID	Type of material	Date	Cell number
LN01	Lymph node	30.05.2011	2.9×10^{7}
LN02	Lymph node	26.07.2011	4×10^{7}
LN03	Lymph node	29.07.2008	4×10^{7}
LN04	Lymph node	10.04.2008	3×10^{7}
PBMC01	PBMC	24.10.2014	3×10^{7}
PBMC02	PBMC	30.04.2010	2×10^{7}
PBMC03	PBMC	09.05.2009	1×10^{7}
PBMC04	PBMC	14.07.2008	4×10^{7}

ID = identifier; LN = Lymph node; PBMC = Peripheral blood mononuclear cells.

Supplementary Table 3. List of canine microRNA targets.

Target miRNA	miRBase accession number	Sequence (5' to 3')	Qiagen GeneGlobe catalog number
cfa-let-7a	MIMAT0006594	UGAGGUAGUAGGUUGUAUAGUU	YP00205727
cfa-let-7b	MIMAT0009836	UGAGGUAGUAGGUUGUGUGGUU	YP00204750
cfa-let-7c	MIMAT0006669	UGAGGUAGUAGGUUGUAUGGUU	YP00204767
cfa-let-7e	MIMAT0006608	UGAGGUAGGAGGUUGUAUAGUU	YP00205711
cfa-let-7f	MIMAT0006610	UGAGGUAGUAGAUUGUAUAGUU	YP00204359
cfa-let-7g	MIMAT0006637	UGAGGUAGUAGUUUGUACAGUU	YP00204565
cfa-miR-10a	MIMAT0006737	UACCCUGUAGAUCCGAAUUUGU	YP02113807
cfa-miR-10b	MIMAT0009837	CCCUGUAGAACCGAAUUUGUGU	YP00205499
cfa-miR-15a	MIMAT0006647	UAGCAGCACAUAAUGGUUUGU	YP02103582
cfa-miR-15b	MIMAT0006676	UAGCAGCACAUCAUGGUUUA	YP00205964
cfa-miR-16	MIMAT0006648	UAGCAGCACGUAAAUAUUGGCG	YP00205702
cfa-miR-17	MIMAT0006649	ACUGCAGUGAAGGCACUUGUAG	YP00206008
cfa-miR-18a	MIMAT0009832	UAAGGUGCAUCUAGUGCAGAUA	YP02119027
cfa-miR-19a	MIMAT0006650	UGUGCAAAUCUAUGCAAAACUGA	YP00205862
cfa-miR-19b	MIMAT0006652	UGUGCAAAUCCAUGCAAAACUG	YP02105441
cfa-miR-20a	MIMAT0006651	UAAAGUGCUUAUAGUGCAGGUAG	YP00204292
cfa-miR-21	MIMAT0006741	UAGCUUAUCAGACUGAUGUUGA	YP00204230
cfa-miR-22	MIMAT0006733	AAGCUGCCAGUUGAAGAACUGU	YP00204606
cfa-miR-23a	MIMAT0006640	AUCACAUUGCCAGGGAUUU	YP00205956
cfa-miR-24	MIMAT0006614	UGGCUCAGUUCAGCAGGAACAGG	YP02114589
cfa-miR-25	MIMAT0006697	CAUUGCACUUGUCUCGGUCUGA	YP00204361
cfa-miR-26a	MIMAT0006595	UUCAAGUAAUCCAGGAUAGGCU	YP00206023
cfa-miR-26b	MIMAT0006678	UUCAAGUAAUUCAGGAUAGGUU	YP00205953
cfa-miR-27a	MIMAT0006641	UUCACAGUGGCUAAGUUCCG	YP00205971
cfa-miR-29a	MIMAT0006626	UAGCACCAUCUGAAAUCGGUUA	YP00204698

cfa-miR-29b	MIMAT0006625	UAGCACCAUUUGAAAUCAGUGUU	YP00204679
cfa-miR-29c	MIMAT0006705	UAGCACCAUUUGAAAUCGGUUA	YP00204729
cfa-miR-30a	MIMAT0006604	UGUAAACAUCCUCGACUGGAAGC	YP02101182
cfa-miR-30b	MIMAT0006617	UGUAAACAUCCUACACUCAGCU	YP00204765
cfa-miR-30d	MIMAT0006616	UGUAAACAUCCCCGACUGGAAGCU	YP02118689
cfa-miR-31	MIMAT0006599	AGGCAAGAUGCUGGCAUAGCUGU	YP02119121
cfa-miR-34a	MIMAT0006690	UGGCAGUGUCUUAGCUGGUUGU	YP00204486
cfa-miR-92a	MIMAT0006653	UAUUGCACUUGUCCCGGCCUGU	YP00204258
cfa-miR-93	MIMAT0006696	CAAAGUGCUGUUCGUGCAGGUAG	YP00204715
cfa-miR-99a	MIMAT0006668	AACCCGUAGAUCCGAUCUUGU	YP00205945
bta-miR-99b	MIMAT0004345	CACCCGUAGAACCGACCUUGCG	YP00205983
cfa-miR-101	MIMAT0006600	UACAGUACUGUGAUAACUGA	YP00205955
cfa-miR-103	MIMAT0006687	AGCAGCAUUGUACAGGGCUAUGA	YP00204063
cfa-miR-106a	MIMAT0006749	AAAGUGCUUACAGUGCAGGUAG	YP02107906
cfa-miR-106b	MIMAT0000680	UAAAGUGCUGACAGUGCAGAU	YP00205884
cfa-miR-107	MIMAT0006666	AGCAGCAUUGUACAGGGCUAU	YP00205950
cfa-miR-122	MIMAT0006619	UGGAGUGUGACAAUGGUGUUUG	YP00205664
cfa-miR-125a	MIMAT0006609	UCCCUGAGACCCUUUAACCUGU	YP02113289
cfa-miR-125b	MIMAT0006670	UCCCUGAGACCCUAACUUGUGA	YP00205713
cfa-miR-126	MIMAT0006730	CAUUAUUACUUUUGGUACGCG	YP00206010
cfa-miR-127	MIMAT0006713	UCGGAUCCGUCUGAGCUUGGCU	YP00204048
cfa-miR-128	MIMAT0006633	UCACAGUGAACCGGUCUCUUU	YP00205995
cfa-miR-130b	MIMAT0006659	CAGUGCAAUGAUGAAAGGGCAU	YP00204317
cfa-miR-132	MIMAT0006732	UAACAGUCUACAGCCAUGGUCGC	YP02104207
cfa-miR-135b	MIMAT0009839	UAUGGCUUUUCAUUCCUAUGUGA	YP00204130
cfa-miR-136	MIMAT0006714	ACUCCAUUUGUUUUGAUGAUGGA	YP00204779
cfa-miR-143	MIMAT0006682	UGAGAUGAAGCACUGUAGCUC	YP00205992
cfa-miR-145	MIMAT0009863	GUCCAGUUUUCCCAGGAAUCCCU	YP00204483

cfa-miR-146a	MIMAT0006684	UGAGAACUGAAUUCCAUGGGUU	YP00204688
cfa-miR-148a	MIMAT0006622	UCAGUGCACUACAGAACUUUGU	YP00205867
cfa-miR-149	MIMAT0009884	UCUGGCUCCGUGUCUUCACUCCC	YP00204321
cfa-miR-150	MIMAT0006602	UCUCCCAACCCUUGUACCAGUG	YP00204660
cfa-miR-151	MIMAT0006615	UCGAGGAGCUCACAGUCUAGU	YP00204007
cfa-miR-155	MIMAT0006671	UUAAUGCUAAUCGUGAUAGGGGU	YP02119772
cfa-miR-181a	MIMAT0006707	AACAUUCAACGCUGUCGGUGAG	YP02117991
cfa-miR-181b	MIMAT0006708	AACAUUCAUUGCUGUCGGUG	YP02110378
cfa-miR-181c	MIMAT0006635	AACAUUCAACCUGUCGGUGAGUU	YP02112709
cfa-miR-181d	MIMAT0006636	AACAUUCAUUGUUGUCGGUGGGU	YP00204789
cfa-miR-182	MIMAT0009841	UUUGGCAAUGGUAGAACUCACACU	YP00206070
cfa-miR-183	MIMAT0006621	UAUGGCACUGGUAGAAUUCACU	YP00206030
cfa-miR-186	MIMAT0006694	CAAAGAAUUCUCCUUUUGGGCU	YP00206053
cfa-miR-197	MIMAT0000227	UUCACCACCUUCUCCACCCAGC	YP00204380
cfa-miR-199	MIMAT0006642	ACAGUAGUCUGCACAUUGGUU	YP00205968
cfa-miR-200b	MIMAT0009864	CAUCUUACUGGGCAGCAUUGGA	YP00204144
cfa-miR-203	MIMAT0009866	GUGAAAUGUUUAGGACCACUAG	YP00205914
cfa-miR-204	MIMAT0006598	UUCCCUUUGUCAUCCUAUGCCU	YP00206072
cfa-miR-206	MIMAT0006606	UGGAAUGUAAGGAAGUGUGUGG	YP00206073
cfa-miR-210	MIMAT0009846	ACUGUGCGUGUGACAGCGGCUGA	YP02119434
cfa-miR-214	MIMAT0009847	ACAGCAGGCACAGACAGGCAGU	YP00204510
cfa-miR-218	MIMAT0006672	UUGUGCUUGAUCUAACCAUGU	YP00206034
cfa-miR-221	MIMAT0006757	AGCUACAUUGUCUGCUGGGUUU	YP02104713
cfa-miR-222	MIMAT0009851	AGCUACAUCUGGCUACUGGGU	YP00204551
cfa-miR-223	MIMAT0009852	UGUCAGUUUGUCAAAUACCCC	YP00205120
cfa-miR-350	MIMAT0006704	UUCACAAAGCCCAUACACUUUU	YP02111685
cfa-miR-363	MIMAT0006750	AAUUGCACGGUAUCCAUCUGUAA	YP02110319
cfa-miR-378	MIMAT0006683	ACUGGACUUGGAGUCAGAAGGC	YP00205946

cfa-miR-423a	MIMAT0006742	UGAGGGGCAGAGAGCGAGACUUU	YP00205624
cfa-miR-450a	MIMAT0001548	UUUUUGCGAUGUGUUCCUAAUA	YP02116559
cfa-miR-450b	MIMAT0006748	UUUUGCAAUAUGUUCCUGAAU	YP02106136
cfa-miR-451	MIMAT0009870	AAACCGUUACCAUUACUGAGUU	YP02119305
cfa-miR-486	MIMAT0011132	UCCUGUACUGAGCUGCCCCGA	YP02119777
cfa-miR-671	MIMAT0009926	UCCGGUUCUCAGGGCUCCACC	YP00204024
cfa-miR-8865	MIMAT0034362	CCUCCCUUUCUCGGACGUCGCCG	YP02121101
cfa-miR-8908a-3p	MIMAT0034429	UAAUUAGGACCUCCCUGAGCGGAGU	YP02121168
**Controls**		**Sequence (5' to 3')**	**Function**
UniSp2		GUACUCGGCUUACGAUCGUAA	Isolation control
UniSp4		GAUGGCAUUCGAUCAGUUCUA	
UniSp5		GAUGCUACGGUCAAUGUCUAAG	
UniSp6		CUAGUCCGAUCUAAGUCUUCGA	RT + qPCR reaction
cel-miR-39-3p	MIMAT0000010	UCACCGGGUGUAAAUCAGCUUG	
UniSp3			Internal plate control (template + assay)
Blank spot			no template control

cfa = *Canis lupus familiaris*; bta = *Bos taurus*; miR = microRNA; Sp = Spike; cel = *Caenorhabditis elegans*.

Supplementary Table 4. Normalization analysis including all patients grouped by entities, excluding miR-122, miR-127, miR-206, miR-8908-3p.

**geNorm**[a]	miRNA ID	miR-16[c]	miR-19a	miR-19b	miR-20a	**miR-21**	miR-22[d]	miR-92a	miR-106a	**miR-146a**	miR-350 [d]
	Stability Factor	0.1395	0.1412	0.1159	0.1457	0.1449	0.1450	0.1438	0.1460	0.1447	0.1457
**Norm Finder**[b]	miRNA ID	let-7g	miR-15a	miR-24	miR-29b	miR-30d	miR-92a	miR-103	miR-148a	miR-197	miR-350 [d]
	Standard Deviation	0.26	0.04	0.22	0.24	0.23	0.05	0.2	0.25	0.21	0.2

MicroRNAs marked in red were use for normalization in previous studies and selected for this study together with two miRNAs marked in bold.

[a]Vandesompele J, De Preter K, Pattyn F, Poppe B, Van Roy N, De Paepe A, Speleman F. Accurate normalization of real-time quantitative RT-PCR data by geometric averaging of multiple internal control genes. Genome Biol. 2002 Jun 18;3(7):RESEARCH0034. PubMed ID: 12184808.

[b]Andersen CL, Jensen JL, Ørntoft TF. Normalization of real-time quantitative reverse transcription-PCR data: a model-based variance estimation approach to identify genes suited for normalization, applied to bladder and colon cancer data sets. Cancer Res. 2004 Aug 1;64(15):5245-50. PubMed ID: 15289330.

[c]Craig KKL, Wood GA, Keller SM, Mutsaers AJ, Wood RD. MicroRNA profiling in canine multicentric lymphoma. PLoS One 14, e0226357 (2019). PubMed ID: 31826004.

[d]Asada H, Tomiyasu H, Uchikai T, Ishihara G, Goto-Koshino Y, Ohno K, Tsujimoto H. Comprehensive analysis of miRNA and protein profiles within exosomes derived from canine lymphoid tumour cell lines. PLoS One 14, e0208567 (2019). PubMed ID: 31034520.

Supplementary Table 5. DNA concentration and quality of studied samples.

Sample ID	Dilution	miRNA ng/µL	miRNA mean	260/280 (mean)	260/230 (mean)
LN01_01	-	7.90	7.60	3.60	0.01
LN01_02	-	7.30			
LN02_01	-	7.70	6.95	2.06	0.03
LN02_02	-	6.20			
LN03_01	-	14.50	16.60	2.00	0.04
LN03_02	-	18.70			
LN04_01	-	10.70	8.55	2.29	0.20
LN04_02	-	6.40			
PBMC01_01	1:10	8.89	8.63	1.61	0.17
PBMC01_02		8.36			
PBMC02_01	-	8.80	8.25	2.42	0.12
PBMC02_02	-	7.70			
PBMC03_01	-	22.80	23.30	1.93	0.04
PBMC03_02	-	23.80			
PBMC04_01	-	20.70	20.15	1.67	0.54
PBMC04_02	-	19.60			
4207_01	1:100	16.60	16.10	2.13	1.77
4207_02		15.60			
3923_01	1:100	17.50	17.70	2.17	1.70
3923_02		17.90			
3739_01	1:100	16.70	17.00	2.14	1.84
3739_02		17.30			
3673_01	1:10	10.50	10.55	2.44	1.30
3673_02		10.60			
3533_01	1:100	10.60	9.35	2.19	0.93
3533_02		8.10			
4217_01	1:100	21.00	21.50	1.97	2.07
4217_02		22.00			
3596_01	1:100	7.90	8.35	2.30	1.67
3596_02		8.80			

3442_01	1:100	11.10	11.00	2.20	0.76
3442_02		10.90			
3998-1_01	1:100	6.10	6.17	2.33	1.69
3998-1_02		6.30			
3998-1_03		6.10			
3998-2_01	1:100	2.7	2.7	2.21	3.06
3998-2_01		2.7			
3867_01	1:100	20.10	20.15	2.09	1.37
3867_02		20.20			
3757_01	1:100	3.30	3.15	2.71	0.41
3757_02		3.00			
3712_01	1:100	11.00	11.30	2.22	0.90
3712_02		11.60			
3616_01	1:100	12.40	12.10	2.08	1.73
3616_02		11.80			
3558_01	1:100	29.40	28.85	1.98	1.94
3558_02		28.30			
3447_01	1:100	17.00	16.30	1.97	1.91
3447_02		15.60			
4244_01	1:100	32.50	32.40	1.99	2.12
4244_02		32.30			
4060_01	1:100	22.60	22.30	2.08	1.86
4060_02		22.00			
3775_01	1:100	21.50	21.05	1.95	2.12
3775_02		20.60			
3715_01	1:100	4.10	4.00	2.46	1.10
3715_02		3.90			
3899_01	1:1000	4.40	3.93	2.24	3.24
3899_02		3.60			
3899_03		3.80			
3840_01	1:100	10.20	10.10	2.21	1.76
3840_02		10.00			

3669_01	1:100	13.10	13.05	2.21	2.00
3669_02		13.00			
3516_01	1:1000	3.10	3.00	3.45	4.28
3516_02		2.90			
3567_01	1:100	17.30	17.45	2.13	1.83
3567_02		17.60			

ID = identifier; LN = Lymph node; PBMC = Peripheral blood mononuclear cells.

Supplementary Table 6. Significantly dysregulated microRNAs in 14 canine Diffuse Large B-cell lymphoma patients compared to 'control group' PBMC.

Patient ID	let-7f	miR-15b	miR-18a	miR-23a	miR-24	miR-26b	miR-27a	miR-30a	miR-34a	miR-99a	miR-106a	miR-143	miR-150	miR-151	miR-181a	miR-181c	miR-197	miR-221	miR-222	miR-223	miR-363	miR-423a	miR-450a	miR-450b
#3616	0.553	0.134	5.242	0.094	0.370	0.245	0.283	45.966	97.511	7.037	2.694	210.474	0.019	0.323	0.243	0.216	0.131	0.336	0.278	0.010	4.555	0.227	0.078	0.130
#3669	0.653	0.149	2.848	0.098	0.231	0.369	0.157	25.857	50.475	3.215	2.868	116.768	0.046	0.485	0.216	0.283	0.374	0.085	0.076	0.012	7.555	0.441	0.378	0.201
#3899	0.246	0.286	2.770	0.072	0.286	0.202	0.663	52.437	27.809	3.593	2.235	257.334	0.049	0.279	0.400	0.417	0.775	0.375	0.383	0.009	14.296	0.940	0.086	0.220
#4060	0.108	0.336	2.549	0.164	0.194	0.080	0.623	33.649	15.216	4.070	1.815	240.101	0.056	0.386	0.562	0.060	0.451	0.278	0.455	0.010	10.109	0.603	0.165	0.194
#4244	0.302	0.059	3.340	0.017	0.307	0.282	0.207	34.595	103.788	3.543	1.879	184.503	0.015	0.363	0.285	0.450	0.171	0.141	0.052	0.014	6.716	0.308	0.044	0.047
#3596	0.452	0.943	0.346	0.567	0.394	0.148	0.528	61.927	28.198	12.510	0.624	240.101	0.866	1.441	0.736	0.400	0.819	0.622	0.605	0.005	4.339	0.927	0.071	0.278
#4217	0.618	0.290	0.717	0.088	0.282	0.226	0.179	25.857	36.695	6.212	3.010	129.562	0.085	0.767	0.223	0.378	1.518	0.983	0.204	0.019	20.930	1.424	0.329	1.286
#3739	0.593	0.074	3.864	0.081	0.329	0.240	0.201	27.906	103.071	3.904	4.347	358.915	0.025	0.287	0.098	0.088	0.166	0.488	0.200	0.006	4.250	0.237	0.042	0.165
#4207	0.286	0.156	8.112	0.171	0.534	0.319	0.456	71.630	78.114	7.648	5.134	612.048	0.016	0.194	0.117	0.731	0.274	0.163	0.217	0.008	14.099	0.525	0.329	0.357
#3923	0.626	0.119	4.959	0.080	0.388	0.254	0.343	78.385	106.706	10.891	3.053	774.703	0.033	0.403	0.159	0.726	0.280	0.405	0.410	0.013	8.098	0.390	0.019	0.075
#3673	0.198	0.296	6.364	0.105	0.158	0.058	0.208	17.298	46.447	3.494	2.567	189.690	0.005	0.577	0.099	0.106	0.093	0.580	0.735	0.026	10.321	0.179	0.358	0.564
#3516	0.488	0.186	4.691	0.063	0.278	0.319	0.172	34.356	49.780	9.680	2.266	216.391	0.031	0.444	0.128	0.405	0.349	1.003	0.386	0.001	4.782	0.349	0.122	0.079
#3567	0.205	0.131	3.249	0.029	0.153	0.169	0.087	14.647	6.309	1.821	2.114	96.169	0.014	0.099	0.106	0.210	0.380	0.597	0.154	0.001	6.532	0.356	0.029	0.113
#3533	0.326	0.898	0.262	0.433	0.271	0.149	0.405	54.663	43.941	19.360	0.655	260.927	0.480	1.063	1.588	0.520	0.988	0.462	0.766	0.007	1.556	1.110	0.097	0.380
Mean	0.404	0.290	3.522	0.147	0.298	0.219	0.322	41.370	56.719	6.927	2.519	277.692	0.124	0.508	0.354	0.356	0.484	0.466	0.351	0.010	8.438	0.572	0.153	0.292
SD	0.179	0.271	2.176	0.152	0.099	0.087	0.178	19.160	33.439	4.641	1.169	184.086	0.236	0.349	0.388	0.205	0.390	0.270	0.219	0.007	4.974	0.367	0.129	0.307
Median	0.389	0.171	3.295	0.091	0.284	0.233	0.246	34.476	48.113	5.141	2.416	228.246	0.032	0.394	0.220	0.389	0.362	0.433	0.331	0.009	7.135	0.415	0.092	0.197

Individual Fold change values [$2^{(-\Delta\Delta Ct)}$] of 14 canine Diffuse Large B-cell lymphoma patients. PBMC = Peripheral blood mononuclear cells; ID = identifier; miR = microRNA; SD = Standard deviation.

Supplementary Table 7. Significantly dysregulated microRNAs in 14 canine Diffuse Large B-cell lymphoma patients compared to ‘control group’ lymph node.

Patient ID	miR-10a	miR-15b	miR-18a	miR-19a	miR-19b	miR-20a	miR-23a	miR-24	miR-26b	miR-27a	miR-34a	miR-93	miR-106b	miR-146a
#3616	0.262	0.181	11.314	9.596	6.158	5.143	0.080	0.467	0.324	0.392	30.013	2.898	3.739	0.081
#3669	0.619	0.203	6.148	3.791	1.831	5.826	0.084	0.291	0.488	0.218	15.536	2.540	2.177	0.115
#3899	0.496	0.389	5.979	4.385	2.974	3.232	0.062	0.361	0.267	0.920	8.559	3.986	2.893	0.127
#4060	0.167	0.456	5.502	3.232	1.756	2.589	0.140	0.245	0.105	0.865	4.683	2.078	1.359	0.183
#4244	0.214	0.080	7.210	7.323	4.967	7.741	0.015	0.387	0.372	0.287	31.945	1.979	2.737	0.179
#3596	0.912	1.281	0.747	0.374	0.374	1.191	0.485	0.497	0.195	0.732	8.679	2.021	0.526	0.206
#4217	0.741	0.394	1.548	1.089	1.151	6.158	0.075	0.356	0.298	0.248	11.294	7.086	1.388	0.196
#3739	0.711	0.101	8.340	11.412	5.866	10.574	0.069	0.415	0.318	0.279	31.724	4.243	2.995	0.089
#4207	0.423	0.211	17.509	8.892	6.331	5.826	0.146	0.674	0.422	0.633	24.042	4.547	6.288	0.038
#3923	0.594	0.162	10.703	8.126	5.990	4.699	0.068	0.490	0.336	0.476	32.843	2.305	3.122	0.109
#3673	0.050	0.403	13.737	11.412	7.582	6.692	0.090	0.199	0.077	0.289	14.296	1.597	2.383	0.262
#3516	0.287	0.253	10.126	6.555	3.636	6.928	0.053	0.351	0.422	0.238	15.322	2.704	4.933	0.122
#3567	0.131	0.178	7.013	4.265	2.399	5.474	0.025	0.193	0.223	0.121	1.942	2.136	1.949	0.281
#3533	0.542	1.221	0.566	0.519	0.433	1.200	0.370	0.342	0.197	0.563	13.524	1.664	0.652	0.154
Mean	0.439	0.394	7.603	5.784	3.675	5.234	0.126	0.376	0.289	0.447	17.457	2.985	2.653	0.153
SD	0.251	0.368	4.697	3.682	2.350	2.469	0.130	0.125	0.116	0.247	10.292	1.458	1.526	0.067
Median	0.459	0.232	7.111	5.470	3.305	5.650	0.078	0.358	0.308	0.341	14.809	2.423	2.560	0.141

Individual Fold change values [$2^{(-\Delta\Delta Ct)}$] of 14 canine Diffuse Large B-cell lymphoma patients; ID = identifier; miR = microRNA; SD = Standard deviation.

Supplementary Table 7. Significantly dysregulated microRNAs in 14 canine Diffuse Large B-cell lymphoma patients compared to 'control group' lymph node (continued).

Patient ID	miR-148a	miR-150	miR-155	miR-181c	miR-181d	miR-221	miR-222	miR-223	miR-350	miR-363	miR-423a	miR-450b	miR-671	miR-8865
#3616	0.523	0.039	0.075	0.180	0.151	0.309	0.317	0.140	3.857	8.831	0.168	0.235	0.248	0.248
#3669	0.434	0.096	0.011	0.236	0.019	0.078	0.086	0.180	3.155	14.647	0.328	0.364	0.490	0.490
#3899	0.320	0.101	0.025	0.347	0.090	0.346	0.436	0.134	2.325	27.713	0.697	0.398	0.177	0.177
#4060	0.523	0.116	0.037	0.050	0.043	0.257	0.519	0.148	1.441	19.596	0.448	0.352	0.218	0.218
#4244	0.333	0.031	0.051	0.375	0.207	0.130	0.059	0.196	3.199	13.019	0.228	0.085	0.173	0.173
#3596	0.481	1.793	0.607	0.333	0.156	0.573	0.689	0.067	1.451	8.412	0.688	0.504	0.042	0.042
#4217	0.140	0.176	0.460	0.315	0.283	0.906	0.232	0.281	3.884	40.574	1.057	2.334	0.085	0.085
#3739	0.362	0.051	0.174	0.074	0.036	0.450	0.227	0.082	3.026	8.239	0.176	0.300	0.337	0.337
#4207	1.091	0.033	0.444	0.609	0.401	0.150	0.247	0.114	4.716	27.332	0.390	0.647	0.165	0.165
#3923	0.862	0.069	0.210	0.605	0.304	0.373	0.467	0.193	2.246	15.698	0.289	0.137	0.247	0.247
#3673	0.275	0.011	0.207	0.089	0.029	0.535	0.837	0.382	5.269	20.008	0.133	1.023	0.325	0.325
#3516	0.200	0.064	0.010	0.338	0.206	0.925	0.439	0.015	4.985	9.270	0.259	0.143	0.159	0.159
#3567	0.251	0.030	0.013	0.175	0.025	0.550	0.176	0.010	4.882	12.663	0.264	0.205	0.140	0.140
#3533	0.431	0.995	0.543	0.434	1.231	0.426	0.872	0.102	1.412	3.016	0.824	0.689	0.133	0.133
Mean	0.445	0.257	0.205	0.297	0.227	0.429	0.400	0.146	3.275	16.358	0.425	0.530	0.210	0.210
SD	0.248	0.490	0.209	0.171	0.301	0.249	0.250	0.096	1.322	9.643	0.272	0.558	0.111	0.111
Median	0.397	0.066	0.125	0.324	0.153	0.399	0.377	0.137	3.177	13.833	0.308	0.358	0.175	0.175

Individual Fold change values [$2^{(-\Delta\Delta Ct)}$] of 14 canine Diffuse Large B-cell lymphoma patients; ID = identifier; miR = microRNA; SD = Standard deviation.

Supplementary Table 8. Shared microRNAs in 14 canine Diffuse Large B-cell lymphoma patients compared to both control groups.

	miR-15b		miR-18a		miR-23a		miR-24		miR-26b		miR-27a		miR-34a		miR-150	
Patient ID	PBMC	LN	PBMC	LN	PBMC	LN	PBMC	LN	PBMC	LN	PBMC	LN	PBMC	LN	PBMC	LN
#3616	0.134	0.181	5.242	11.314	0.094	0.080	0.370	0.467	0.245	0.324	0.283	0.392	97.511	30.013	0.019	0.039
#3669	0.149	0.203	2.848	6.148	0.098	0.084	0.231	0.291	0.369	0.488	0.157	0.218	50.475	15.536	0.046	0.096
#3899	0.286	0.389	2.770	5.979	0.072	0.062	0.286	0.361	0.202	0.267	0.663	0.920	27.809	8.559	0.049	0.101
#4060	0.336	0.456	2.549	5.502	0.164	0.140	0.194	0.245	0.080	0.105	0.623	0.865	15.216	4.683	0.056	0.116
#4244	0.059	0.080	3.340	7.210	0.017	0.015	0.307	0.387	0.282	0.372	0.207	0.287	103.788	31.945	0.015	0.031
#3596	0.943	1.281	0.346	0.747	0.567	0.485	0.394	0.497	0.148	0.195	0.528	0.732	28.198	8.679	0.866	1.793
#4217	0.290	0.394	0.717	1.548	0.088	0.075	0.282	0.356	0.226	0.298	0.179	0.248	36.695	11.294	0.085	0.176
#3739	0.074	0.101	3.864	8.340	0.081	0.069	0.329	0.415	0.240	0.318	0.201	0.279	103.071	31.724	0.025	0.051
#4207	0.156	0.211	8.112	17.509	0.171	0.146	0.534	0.674	0.319	0.422	0.456	0.633	78.114	24.042	0.016	0.033
#3923	0.119	0.162	4.959	10.703	0.080	0.068	0.388	0.490	0.254	0.336	0.343	0.476	106.706	32.843	0.033	0.069
#3673	0.296	0.403	6.364	13.737	0.105	0.090	0.158	0.199	0.058	0.077	0.208	0.289	46.447	14.296	0.005	0.011
#3516	0.186	0.253	4.691	10.126	0.063	0.053	0.278	0.351	0.319	0.422	0.172	0.238	49.780	15.322	0.031	0.064
#3567	0.131	0.178	3.249	7.013	0.029	0.025	0.153	0.193	0.169	0.223	0.087	0.121	6.309	1.942	0.014	0.030
#3533	0.898	1.221	0.262	0.566	0.433	0.370	0.271	0.342	0.149	0.197	0.405	0.563	43.941	13.524	0.480	0.995
Mean	0.290	0.394	3.522	7.603	0.147	0.126	0.298	0.376	0.219	0.289	0.322	0.447	56.719	17.457	0.124	0.257
SD	0.271	0.368	2.176	4.697	0.152	0.130	0.099	0.125	0.087	0.116	0.178	0.247	33.439	10.292	0.236	0.490
Median	0.171	0.232	3.295	7.111	0.091	0.078	0.284	0.358	0.233	0.308	0.246	0.341	48.113	14.809	0.032	0.066

Individual Fold change values [$2^{(-\Delta\Delta Ct)}$] of 14 canine Diffuse Large B-cell lymphoma patients; ID = identifier; miR = microRNA; PBMC = Peripheral blood mononuclear cells; LN = lymph node; SD = Standard deviation.

Supplementary Table 8. Shared microRNAs in 14 canine Diffuse Large B-cell lymphoma patients in both control groups (continued).

	miR-181c		miR-221		miR-222		miR-223		miR-363		miR-423a		miR-450b	
Patient ID	PBMC	LN	PBMC	LN	PBMC	LN	PBMC	LN	PBMC	LN	PBMC	LN	PBMC	LN
#3616	0.216	0.180	0.336	0.309	0.278	0.317	0.010	0.140	4.555	8.831	0.227	0.168	0.130	0.235
#3669	0.283	0.236	0.085	0.078	0.076	0.086	0.012	0.180	7.555	14.647	0.441	0.328	0.201	0.364
#3899	0.417	0.347	0.375	0.346	0.383	0.436	0.009	0.134	14.296	27.713	0.940	0.697	0.220	0.398
#4060	0.060	0.050	0.278	0.257	0.455	0.519	0.010	0.148	10.109	19.596	0.603	0.448	0.194	0.352
#4244	0.450	0.375	0.141	0.130	0.052	0.059	0.014	0.196	6.716	13.019	0.308	0.228	0.047	0.085
#3596	0.400	0.333	0.622	0.573	0.605	0.689	0.005	0.067	4.339	8.412	0.927	0.688	0.278	0.504
#4217	0.378	0.315	0.983	0.906	0.204	0.232	0.019	0.281	20.930	40.574	1.424	1.057	1.286	2.334
#3739	0.088	0.074	0.488	0.450	0.200	0.227	0.006	0.082	4.250	8.239	0.237	0.176	0.165	0.300
#4207	0.731	0.609	0.163	0.150	0.217	0.247	0.008	0.114	14.099	27.332	0.525	0.390	0.357	0.647
#3923	0.726	0.605	0.405	0.373	0.410	0.467	0.013	0.193	8.098	15.698	0.390	0.289	0.075	0.137
#3673	0.106	0.089	0.580	0.535	0.735	0.837	0.026	0.382	10.321	20.008	0.179	0.133	0.564	1.023
#3516	0.405	0.338	1.003	0.925	0.386	0.439	0.001	0.015	4.782	9.270	0.349	0.259	0.079	0.143
#3567	0.210	0.175	0.597	0.550	0.154	0.176	0.001	0.010	6.532	12.663	0.356	0.264	0.113	0.205
#3533	0.520	0.434	0.462	0.426	0.766	0.872	0.007	0.102	1.556	3.016	1.110	0.824	0.380	0.689
Mean	0.356	0.297	0.466	0.429	0.351	0.400	0.010	0.146	8.438	16.358	0.572	0.425	0.292	0.530
SD	0.205	0.171	0.270	0.249	0.219	0.250	0.007	0.096	4.974	9.643	0.367	0.272	0.307	0.558
Median	0.389	0.324	0.433	0.399	0.331	0.377	0.009	0.137	7.135	13.833	0.415	0.308	0.197	0.358

Individual Fold change values [$2^{(-\Delta\Delta Ct)}$] of 14 canine Diffuse Large B-cell lymphoma patients; ID = identifier; miR = microRNA; PBMC = Peripheral blood mononuclear cells; LN = lymph node; SD = Standard deviation.

miRCURY LNA miRNA Expression Analysis Report 1

Sabine Hammer

03-03-2023

Table of Contents

Introduction	3
Summary and workflow	4
miRNA Table	5
Data analysis setup	6
Data quality control (QC)	8
Normalization analysis	10
Result	12
Fold regulation and p-value	12
Scatter Plot	18
Volcano Plot	30
Principle Component Analysis (PCA)	38
Clustergram	39
What's next	40
miRNA Expression	41

Introduction

Custom arrays

The Custom miRCURY LNA miRNA PCR Panels are reliable tools for studying and verifying miRNA expression biomarkers in large numbers of biological samples. Whether the biomarkers are from a proprietary collection, literature research or microarray analysis, QIAGEN's expertise in real-time RT-PCR detection provides quality, flexibility and consistency in biomarker validation experiments. Each Custom miRCURY LNA miRNA PCR Panel contains assays for a list of your chosen miRNAs of interest as well as for housekeeping (reference) miRNAs. In addition, each panel should also contain a set of proprietary controls to monitor first strand synthesis and real-time PCR efficiency. The qPCR Assays used in these panels are laboratory-verified and optimized to work under standard conditions enabling many miRNAs to be assayed simultaneously. Their specificity is guaranteed when a miRCURY LNA SYBR Green Master Mix is used as part of the complete RT-PCR system protocol.

In this study, 92 miRNAs were profiled on 32 samples.

Summary and workflow

Custom arrays

1. miRNA was isolated using an extraction kit per the manufacturer's instructions.
2. miRNA quality and quantity was determined and was reverse transcribed using the miRCURY LNA RT Kit.
3. The cDNA in combination with a miRCURY LNA Probe PCR Kit was used on a Custom miRCURY LNA miRNA Probe PCR Panel.

C_T values were exported to an Excel file to create a table of C_T values. This table was then uploaded on to the data analysis web portal at http://www.qiagen.com/geneglobe. Samples were assigned to controls and test groups. C_T values were normalized based on the geNorm (Pre-Defined Reference miRNAs Only) method.

The data analysis web portal calculates fold change/regulation using delta-delta C_T method, in which delta C_T is calculated between miRNA of interest and an average of reference miRNAs, followed by delta-delta C_T calculations (delta C_T (Test Group)-delta C_T (Control Group)). Fold Change is then calculated using 2^ (-delta-delta C_T) formula. The data analysis web portal also plots scatter plots, volcano plots, a principle component analysis and a clustergram.

The data analysis report was exported from the QIAGEN web portal at GeneGlobe.

miRNA Table

Position	miRNA ID	Position	miRNA ID	Position	miRNA ID	Position	miRNA ID
1	cfa-let-7a	2	cfa-let-7b	3	cfa-let-7c	4	cfa-let-7e
5	cfa-let-7f	6	cfa-let-7g	7	cfa-miR-10a	8	cfa-miR-10b
9	cfa-miR-15a	10	cfa-miR-15b	11	cfa-miR-16	12	cfa-miR-17
13	cfa-miR-18a	14	cfa-miR-19a	15	cfa-miR-19b	16	cfa-miR-20a
17	cfa-miR-21	18	cfa-miR-22	19	cfa-miR-23a	20	cfa-miR-24
21	cfa-miR-25	22	cfa-miR-26a	23	cfa-miR-26b	24	cfa-miR-27a
25	cfa-miR-29a	26	cfa-miR-29b	27	cfa-miR-29c	28	cfa-miR-30a
29	cfa-miR-30b	30	cfa-miR-30d	31	cfa-miR-31	32	cfa-miR-34a
33	cfa-miR-92a	34	cfa-miR-93	35	cfa-miR-99a	36	bta-miR-99b
37	cfa-miR-101	38	cfa-miR-103	39	cfa-miR-106a	40	cfa-miR-106b
41	cfa-miR-107	43	cfa-miR-125a	44	cfa-miR-125b	45	cfa-miR-126
47	cfa-miR-128	48	cfa-miR-130b	49	cfa-miR-132	50	cfa-miR-135b
51	cfa-miR-136	52	cfa-miR-143	53	cfa-miR-145	54	cfa-miR-146a
55	cfa-miR-148a	56	cfa-miR-149	57	cfa-miR-150	58	cfa-miR-151
59	cfa-miR-155	60	cfa-miR-181a	61	cfa-miR-181b	62	cfa-miR-181c
63	cfa-miR-181d	64	cfa-miR-182	65	cfa-miR-183	66	cfa-miR-186
67	cfa-miR-197	68	cfa-miR-199	69	cfa-miR-200b	70	cfa-miR-203
71	cfa-miR-204	73	cfa-miR-210	74	cfa-miR-214	75	cfa-miR-218
76	cfa-miR-221	77	cfa-miR-222	78	cfa-miR-223	79	cfa-miR-350
80	cfa-miR-363	81	cfa-miR-378	82	cfa-miR-423a	83	cfa-miR-450a
84	cfa-miR-450b	85	cfa-miR-451	86	cfa-miR-486	87	cfa-miR-671
88	cfa-miR-8865	90	UniSp2	92	UniSp4	94	UniSp5
96	UniSp6	98	cel-miR-39-3p	100	UniSP3	102	BlankSpot

Data analysis setup

Sample management

Sample ID	Sample Name	Group
1	LN 01	Exclude Sample
2	LN 02	Exclude Sample
3	LN 03	Exclude Sample
4	LN 04	Exclude Sample
5	PBMC 01	Control Group
6	PBMC 02	Control Group
7	PBMC 03	Control Group
8	PBMC 04	Control Group
9	4207 (DLBCL)	Group 3
10	3923 (DLBCL)	Group 3
11	3673 (DLBCL)	Group 3
12	3739 (DLBCL)	Group 3
13	3533 (DLBCL)	Group 3
14	4217 (DLBCL)	Group 3
15	3596 (DLBCL)	Group 3
16	3442 (TZL)	Group 5
17	3998 (PTCL)	Group 4
18	3867 (MZL)	Group 6
19	3757 (PTCL)	Group 4
20	3712 (TZL)	Group 5
21	3616 (DLBCL)	Group 3
22	3558 (PTCL)	Group 4
23	3447 (PTCL)	Group 4
24	4244 (DLBCL)	Group 3
25	4060 (DLBCL)	Group 3
26	3899 (DLBCL)	Group 3
27	3775 (PTCL)	Group 4
28	3715 (MZL)	Group 6
29	3840 (PTCL)	Group 4
30	3669 (DLBCL)	Group 3
31	3516 (DLBCL)	Group 3
32	3567 (DLBCL)	Group 3

Lower limit of detection

The C_T cut-off was set to 40

Data quality control (QC)

Internal Amplification Controls

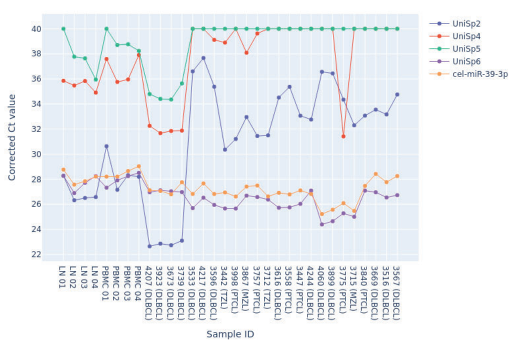

Call Rate (Gene Quality) Plot

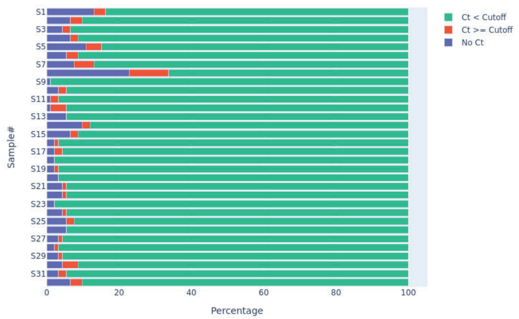

Sample#	Sample ID	Frequency of Missing Data
S1	LN 01	13.04%
S2	LN 02	6.52%
S3	LN 03	4.35%
S4	LN 04	6.52%
S5	PBMC 01	10.87%
S6	PBMC 02	5.43%
S7	PBMC 03	7.61%
S8	PBMC 04	22.83%
S9	4207 (DLBCL)	1.09%

Sample#	Sample ID	Frequency of Missing Data
S10	3923 (DLBCL)	3.26%
S11	3673 (DLBCL)	1.09%
S12	3739 (DLBCL)	1.09%
S13	3533 (DLBCL)	5.43%
S14	4217 (DLBCL)	9.78%
S15	3596 (DLBCL)	6.52%
S16	3442 (TZL)	2.17%
S17	3998 (PTCL)	2.17%
S18	3867 (MZL)	2.17%
S19	3757 (PTCL)	2.17%
S20	3712 (TZL)	3.26%
S21	3616 (DLBCL)	4.35%
S22	3558 (PTCL)	4.35%
S23	3447 (PTCL)	2.17%
S24	4244 (DLBCL)	4.35%
S25	4060 (DLBCL)	5.43%
S26	3899 (DLBCL)	5.43%
S27	3775 (PTCL)	3.26%
S28	3715 (MZL)	2.17%
S29	3840 (PTCL)	3.26%
S30	3669 (DLBCL)	4.35%
S31	3516 (DLBCL)	3.26%
S32	3567 (DLBCL)	6.52%

Normalization analysis

geNorm (Pre-Defined Reference miRNAs Only)

Groups	Samples	11	17	18	54	79	Arithmetic Mean	Average Arithmetic Mean
Stability Factor		0.2006	0.2012	0.2033	0.2032	0.2033		
Control Group	PBMC 01	27.27	28.20	34.99	37.81	35.32	32.72	32.26
Control Group	PBMC 02	24.08	26.38	32.55	33.74	32.91	29.93	
Control Group	PBMC 03	27.04	28.33	34.12	34.62	34.02	31.63	
Control Group	PBMC 04	29.91	30.51	37.12	38.18	38.06	34.76	
Group 3	3533 (DLBCL)	26.27	27.68	33.02	34.72	34.42	31.22	29.88
Group 3	3739 (DLBCL)	24.89	25.78	33.03	34.31	32.11	30.02	
Group 3	3596 (DLBCL)	25.62	27.94	32.50	33.86	33.92	30.77	
Group 3	3673 (DLBCL)	25.19	26.37	33.12	32.30	30.85	29.56	
Group 3	3923 (DLBCL)	24.14	24.35	31.70	32.89	31.41	28.90	
Group 3	4207 (DLBCL)	24.21	25.08	32.26	35.03	30.95	29.51	
Group 3	3567 (DLBCL)	25.19	26.23	32.96	32.10	30.87	29.47	
Group 3	3516 (DLBCL)	25.06	24.56	32.05	32.78	30.32	28.95	
Group 3	4217 (DLBCL)	27.32	31.00	35.77	36.13	34.71	32.98	
Group 3	4244 (DLBCL)	23.36	24.21	31.76	31.48	30.22	28.21	
Group 3	4060 (DLBCL)	26.77	25.25	32.31	33.51	33.41	30.25	
Group 3	3899 (DLBCL)	26.00	26.28	32.77	34.20	32.90	30.43	
Group 3	3669 (DLBCL)	23.85	24.68	32.71	32.99	31.09	29.06	
Group 3	3616 (DLBCL)	23.77	24.76	32.18	33.39	30.71	28.96	
Group 4	3998 (PTCL)	20.75	21.87	28.34	26.87	27.87	25.14	27.67
Group 4	3840 (PTCL)	22.58	23.85	30.85	29.53	29.25	27.21	
Group 4	3775 (PTCL)	23.75	23.66	31.47	30.26	31.24	28.08	
Group 4	3757 (PTCL)	24.58	23.85	31.24	31.20	31.88	28.55	
Group 4	3447 (PTCL)	22.79	25.48	30.77	31.68	30.41	28.23	
Group 4	3558 (PTCL)	24.10	24.29	31.75	32.74	31.07	28.79	
Group 5	3712 (TZL)	22.62	22.30	30.06	30.16	30.83	27.19	27.22
Group 5	3442 (TZL)	22.76	22.59	29.86	30.75	30.24	27.24	
Group 6	3867 (MZL)	23.89	22.57	30.90	30.56	31.12	27.81	27.67
Group 6	3715 (MZL)	22.63	23.16	30.34	31.31	30.22	27.53	

miRNA #	miRNA ID
11	cfa-miR-16
17	cfa-miR-21

miRNA #	miRNA ID
18	cfa-miR-22
54	cfa-miR-146a
79	cfa-miR-350

This method calculates a normalization factor based on multiple reference miRNA for more accurate and reliable normalization of miRNA expression data. It is based on the principle that the expression ratio of any two reference miRNAs (using standard deviation of log-transformed reference miRNA ratios) should be identical in all samples. Stability measures will be returned which are the average pairwise variation between a reference miRNA and all other reference miRNAs using stepwise exclusion of the worst scoring miRNAs. The predefined miRNAs or up to ten miRNAs from the entire panel with the best gNorm-calculated stability measures are listed if their value is less than 1.5. For more details, see:

Reference: Vandesompele J, De Preter K, Pattyn F, Poppe B, Van Roy N, De Paepe A, Speleman F. Accurate normalization of real-time quantitative RT-PCR data by geometric averaging of multiple internal control miRNAs. Genome Biol. 2002 Jun 18;3(7):RESEARCH0034. PubMed ID: 12184808.

Result

Fold regulation and p-value

Test Group	Control Group	Fold Regulation Threshold	p-Value Threshold
Group 3	Control Group	2	0.05

Position	miRNA ID	Fold Regulation	p-Value	Comments
13	cfa-miR-18a	2.49	0.048240	
28	cfa-miR-30a	47.83	0.001155	A
32	cfa-miR-34a	50.61	0.006348	A
35	cfa-miR-99a	5.66	0.029435	A
39	cfa-miR-106a	2.19	0.042318	
52	cfa-miR-143	393.10	0.012269	A
80	cfa-miR-363	7.03	0.013027	
5	cfa-let-7f	-2.80	0.003003	
10	cfa-miR-15b	-4.83	0.005393	
19	cfa-miR-23a	-9.55	0.000060	
20	cfa-miR-24	-3.54	0.000001	
23	cfa-miR-26b	-5.11	0.002134	
24	cfa-miR-27a	-3.64	0.000008	
57	cfa-miR-150	-24.41	0.000012	
58	cfa-miR-151	-2.20	0.013498	
60	cfa-miR-181a	-4.17	0.004455	
62	cfa-miR-181c	-3.09	0.003276	
67	cfa-miR-197	-2.80	0.023158	
76	cfa-miR-221	-2.64	0.004733	
77	cfa-miR-222	-3.65	0.000318	
78	cfa-miR-223	-138.82	0.000000	
82	cfa-miR-423a	-2.12	0.035101	
83	cfa-miR-450a	-10.01	0.003185	
84	cfa-miR-450b	-6.30	0.003024	

Test Group	Control Group	Fold Regulation Threshold	p-Value Threshold
Group 4	Control Group	2	0.05

Position	miRNA ID	Fold Regulation	p-Value	Comments
13	cfa-miR-18a	2.09	0.009117	
28	cfa-miR-30a	32.97	0.018914	A
32	cfa-miR-34a	4.50	0.028340	A
43	cfa-miR-125a	3.45	0.011589	
44	cfa-miR-125b	4.47	0.018125	
52	cfa-miR-143	432.19	0.004948	A
53	cfa-miR-145	398.57	0.010120	A
60	cfa-miR-181a	2.45	0.044627	
68	cfa-miR-199	7.30	0.049231	A
74	cfa-miR-214	34.58	0.008617	A
19	cfa-miR-23a	-5.68	0.019382	A
20	cfa-miR-24	-2.43	0.002720	
21	cfa-miR-25	-2.12	0.021101	
24	cfa-miR-27a	-2.15	0.017914	
25	cfa-miR-29a	-3.58	0.000170	
26	cfa-miR-29b	-2.88	0.017736	
27	cfa-miR-29c	-2.38	0.004773	
30	cfa-miR-30d	-3.59	0.000188	
31	cfa-miR-31	-9.21	0.013013	
33	cfa-miR-92a	-2.87	0.002382	
41	cfa-miR-107	-2.18	0.007864	
57	cfa-miR-150	-29.51	0.000045	
67	cfa-miR-197	-7.12	0.000070	
77	cfa-miR-222	-2.76	0.010684	
78	cfa-miR-223	-181.72	0.000133	
82	cfa-miR-423a	-8.77	0.000943	

Test Group	Control Group	Fold Regulation Threshold	p-Value Threshold
Group 5	Control Group	2	0.05

Position	miRNA ID	Fold Regulation	p-Value	Comments
3	cfa-let-7c	2.64	nan	A
7	cfa-miR-10a	2.60	nan	A
8	cfa-miR-10b	10.38	nan	A
28	cfa-miR-30a	54.55	nan	A
32	cfa-miR-34a	6.53	nan	A
35	cfa-miR-99a	6.39	nan	A
36	bta-miR-99b	14.90	nan	A
37	cfa-miR-101	2.45	nan	A
43	cfa-miR-125a	4.09	nan	
44	cfa-miR-125b	11.58	nan	
45	cfa-miR-126	6.66	nan	
52	cfa-miR-143	612.46	nan	A
53	cfa-miR-145	928.49	nan	A
68	cfa-miR-199	10.70	nan	A
74	cfa-miR-214	62.01	nan	A
85	cfa-miR-451	14.19	nan	
5	cfa-let-7f	-2.01	nan	
10	cfa-miR-15b	-3.61	nan	
16	cfa-miR-20a	-2.24	nan	
21	cfa-miR-25	-2.74	nan	
23	cfa-miR-26b	-4.32	nan	
30	cfa-miR-30d	-6.98	nan	
31	cfa-miR-31	-2.27	nan	
33	cfa-miR-92a	-7.86	nan	
34	cfa-miR-93	-3.50	nan	
38	cfa-miR-103	-2.27	nan	
39	cfa-miR-106a	-3.17	nan	
47	cfa-miR-128	-3.43	nan	A
48	cfa-miR-130b	-4.09	nan	A
57	cfa-miR-150	-2.70	nan	
59	cfa-miR-155	-6.90	nan	B
60	cfa-miR-181a	-4.53	nan	
61	cfa-miR-181b	-2.33	nan	
63	cfa-miR-181d	-2.10	nan	A
64	cfa-miR-182	-2.25	nan	A

Position	miRNA ID	Fold Regulation	p-Value	Comments
65	cfa-miR-183	-3.49	nan	B
67	cfa-miR-197	-6.01	nan	
69	cfa-miR-200b	-5.90	nan	B
73	cfa-miR-210	-3.39	nan	
78	cfa-miR-223	-72.21	nan	
81	cfa-miR-378	-3.23	nan	
82	cfa-miR-423a	-7.52	nan	
83	cfa-miR-450a	-22.87	nan	B
84	cfa-miR-450b	-16.36	nan	B
86	cfa-miR-486	-2.19	nan	
88	cfa-miR-8865	-60.75	nan	B

Test Group	Control Group	Fold Regulation Threshold	p-Value Threshold
Group 6	Control Group	2	0.05

Position	miRNA ID	Fold Regulation	p-Value	Comments
3	cfa-let-7c	2.57	nan	A
7	cfa-miR-10a	6.01	nan	A
8	cfa-miR-10b	3.31	nan	A
28	cfa-miR-30a	81.80	nan	A
32	cfa-miR-34a	12.61	nan	A
35	cfa-miR-99a	5.14	nan	A
36	bta-miR-99b	7.87	nan	A
37	cfa-miR-101	3.54	nan	A
43	cfa-miR-125a	3.32	nan	
44	cfa-miR-125b	8.43	nan	
45	cfa-miR-126	4.98	nan	
52	cfa-miR-143	643.49	nan	A
53	cfa-miR-145	940.67	nan	A
55	cfa-miR-148a	2.34	nan	
68	cfa-miR-199	5.15	nan	A
74	cfa-miR-214	50.56	nan	A
75	cfa-miR-218	2.00	nan	A
85	cfa-miR-451	62.15	nan	
5	cfa-let-7f	-2.29	nan	
10	cfa-miR-15b	-4.29	nan	
19	cfa-miR-23a	-2.94	nan	A
20	cfa-miR-24	-2.75	nan	
21	cfa-miR-25	-2.90	nan	
23	cfa-miR-26b	-4.99	nan	
30	cfa-miR-30d	-4.55	nan	
31	cfa-miR-31	-2.51	nan	
33	cfa-miR-92a	-5.06	nan	
34	cfa-miR-93	-3.18	nan	
38	cfa-miR-103	-2.36	nan	
41	cfa-miR-107	-2.47	nan	
47	cfa-miR-128	-3.17	nan	A
48	cfa-miR-130b	-2.22	nan	A
51	cfa-miR-136	-2.27	nan	B
56	cfa-miR-149	-2.47	nan	B
57	cfa-miR-150	-18.53	nan	

Position	miRNA ID	Fold Regulation	p-Value	Comments
59	cfa-miR-155	-7.04	nan	B
60	cfa-miR-181a	-3.93	nan	
61	cfa-miR-181b	-3.01	nan	
67	cfa-miR-197	-9.55	nan	
69	cfa-miR-200b	-2.78	nan	B
76	cfa-miR-221	-4.27	nan	
77	cfa-miR-222	-2.88	nan	
78	cfa-miR-223	-98.71	nan	
81	cfa-miR-378	-4.73	nan	
82	cfa-miR-423a	-7.33	nan	
83	cfa-miR-450a	-26.89	nan	B
84	cfa-miR-450b	-32.19	nan	B
88	cfa-miR-8865	-30.77	nan	B

Fold-Change (2^(-Delta-Delta C_T)) is the normalized miRNA expression (2^(- Delta C_T)) in a Test Sample divided the normalized miRNA expression (2^(- Delta C_T)) in the Control Sample. Fold-Regulation represents fold-change results in a biologically meaningful way. Fold-change values greater than one indicates a positive- or an up-regulation, and the fold-regulation is equal to the fold-change. Fold-change values less than one indicate a negative or down-regulation, and the fold-regulation is the negative inverse of the fold-change.

The p values are calculated based on a Student's t-test of the replicate 2^(- Delta C_T) values for each miRNA in the Control Group and each Test Group, and p values less than 0.05 are indicated in red. The p-value calculation used is based on parametric, unpaired, two-sample equal variance, two-tailed distribution.

Scatter Plot

Test Group	Control Group	Fold Regulation Threshold
Group 3	Control Group	2

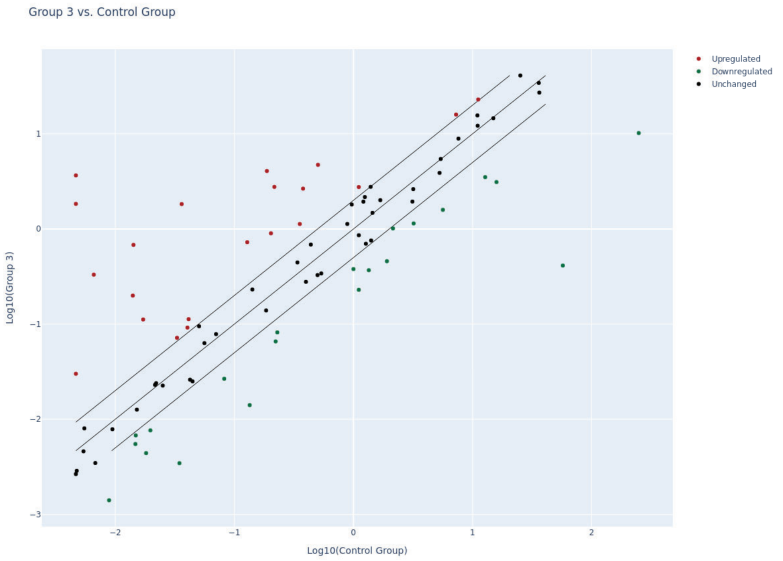

The Scatter Plot compares the normalized expression of every miRNA in the PCR Panel between the two selected Groups by plotting them against one another to quickly visualize large miRNA expression changes. The center diagonal line indicates unchanged miRNA expression, while the outer diagonal lines indicate the selected fold regulation threshold. miRNAs with data points beyond the outer lines in the upper left and lower right corners are up-regulated or down-regulated, respectively, by more than the fold regulation threshold in the y-axis Group relative to the x-axis Group.

miRNAs Over-Expressed in Group 3 vs. Control Group

Position	miRNA ID	Fold Regulation	Comments	miRCURY LNA miRNA PCR Assay Catalog #
3	cfa-let-7c	2.73	A	YP00204767
8	cfa-miR-10b	6.44	A	YP00205499
12	cfa-miR-17	2.28	A	YP00206008
13	cfa-miR-18a	2.49		YP02119027
14	cfa-miR-19a	2.06		YP00205862
28	cfa-miR-30a	47.83	A	YP02101182
31	cfa-miR-31	12.77		YP02119121
32	cfa-miR-34a	50.61	A	YP00204486
35	cfa-miR-99a	5.66	A	YP00205945
36	bta-miR-99b	14.21	A	YP00205983
39	cfa-miR-106a	2.19		YP02107906
43	cfa-miR-125a	4.44		YP02113289
44	cfa-miR-125b	9.38		YP00205713
45	cfa-miR-126	3.18		YP00206010
52	cfa-miR-143	393.10	A	YP00205992
53	cfa-miR-145	782.86	A	YP00204483
68	cfa-miR-199	6.52	A	YP00205968
74	cfa-miR-214	50.12	A	YP00204510
80	cfa-miR-363	7.03		YP02110319
85	cfa-miR-451	21.68		YP02119305
87	cfa-miR-671	2.18	A	YP00204024

miRNAs Under-Expressed in Group 3 vs. Control Group

Position	miRNA ID	Fold Regulation	Comments	miRCURY LNA miRNA PCR Assay Catalog #
5	cfa-let-7f	-2.80		YP00204359
10	cfa-miR-15b	-4.83		YP00205964
19	cfa-miR-23a	-9.55		YP00205956
20	cfa-miR-24	-3.54		YP02114589
23	cfa-miR-26b	-5.11		YP00205953
24	cfa-miR-27a	-3.64		YP00205971
57	cfa-miR-150	-24.41		YP00204660
58	cfa-miR-151	-2.20		YP00204007
59	cfa-miR-155	-2.58	B	YP02119772
60	cfa-miR-181a	-4.17		YP02117991
61	cfa-miR-181b	-3.38		YP02110378
62	cfa-miR-181c	-3.09		YP02112709
63	cfa-miR-181d	-4.12	B	YP00204789
67	cfa-miR-197	-2.80		YP00204380
70	cfa-miR-203	-2.69	B	YP00205914
76	cfa-miR-221	-2.64		YP02104713
77	cfa-miR-222	-3.65		YP00204551
78	cfa-miR-223	-138.82		YP00205120
82	cfa-miR-423a	-2.12		YP00205624
83	cfa-miR-450a	-10.01		YP02116559
84	cfa-miR-450b	-6.30		YP02106136

Test Group	Control Group	Fold Regulation Threshold
Group 4	Control Group	2

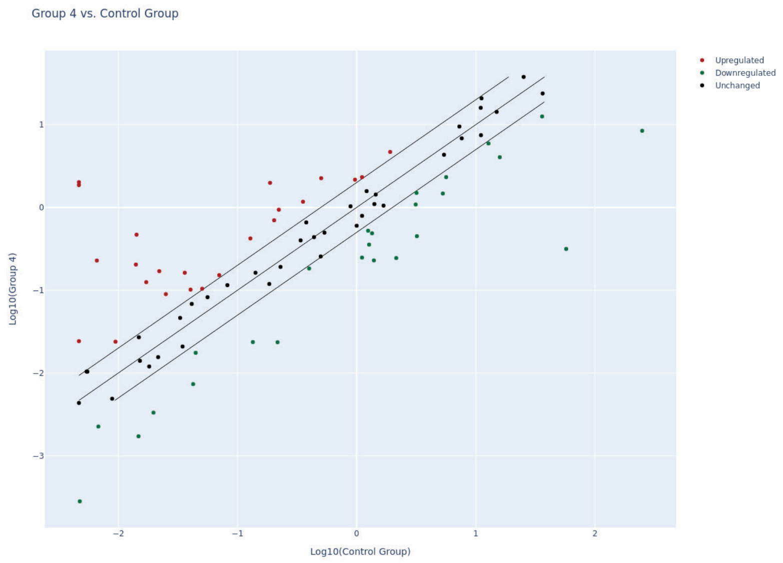

The Scatter Plot compares the normalized expression of every miRNA in the PCR Panel between the two selected Groups by plotting them against one another to quickly visualize large miRNA expression changes. The center diagonal line indicates unchanged miRNA expression, while the outer diagonal lines indicate the selected fold regulation threshold. miRNAs with data points beyond the outer lines in the upper left and lower right corners are up-regulated or down-regulated, respectively, by more than the fold regulation threshold in the y-axis Group relative to the x-axis Group.

miRNAs Over-Expressed in Group 4 vs. Control Group

Position	miRNA ID	Fold Regulation	Comments	miRCURY LNA miRNA PCR Assay Catalog #
8	cfa-miR-10b	5.19	A	YP00205499
12	cfa-miR-17	2.52	A	YP00206008
13	cfa-miR-18a	2.09		YP02119027
28	cfa-miR-30a	32.97	A	YP02101182
32	cfa-miR-34a	4.50	A	YP00204486
35	cfa-miR-99a	3.29	A	YP00205945
36	bta-miR-99b	14.55	A	YP00205983
37	cfa-miR-101	2.06	A	YP00205955
40	cfa-miR-106b	2.23		YP00205884
43	cfa-miR-125a	3.45		YP02113289
44	cfa-miR-125b	4.47		YP00205713
45	cfa-miR-126	3.31		YP00206010
49	cfa-miR-132	7.71	A	YP02104207
51	cfa-miR-136	2.52	A	YP00204779
52	cfa-miR-143	432.19	A	YP00205992
53	cfa-miR-145	398.57	A	YP00204483
54	cfa-miR-146a	2.17	A	YP00204688
60	cfa-miR-181a	2.45		YP02117991
61	cfa-miR-181b	4.22		YP02110378
64	cfa-miR-182	3.58	A	YP00206070
68	cfa-miR-199	7.30	A	YP00205968
74	cfa-miR-214	34.58	A	YP00204510
85	cfa-miR-451	10.56		YP02119305

miRNAs Under-Expressed in Group 4 vs. Control Group

Position	miRNA ID	Fold Regulation	Comments	miRCURY LNA miRNA PCR Assay Catalog #
10	cfa-miR-15b	-4.47		YP00205964
19	cfa-miR-23a	-5.68	A	YP00205956
20	cfa-miR-24	-2.43		YP02114589
21	cfa-miR-25	-2.12		YP00204361
23	cfa-miR-26b	-3.93		YP00205953
24	cfa-miR-27a	-2.15		YP00205971
25	cfa-miR-29a	-3.58		YP00204698
26	cfa-miR-29b	-2.88		YP00204679
27	cfa-miR-29c	-2.38		YP00204729
30	cfa-miR-30d	-3.59		YP02118689
31	cfa-miR-31	-9.21		YP02119121
33	cfa-miR-92a	-2.87		YP00204258
41	cfa-miR-107	-2.18		YP00205950
56	cfa-miR-149	-3.01	B	YP00204321
57	cfa-miR-150	-29.51		YP00204660
59	cfa-miR-155	-5.93	B	YP02119772
67	cfa-miR-197	-7.12		YP00204380
69	cfa-miR-200b	-16.84	B	YP00204144
70	cfa-miR-203	-8.59	B	YP00205914
71	cfa-miR-204	-2.54	A	YP00206072
77	cfa-miR-222	-2.76		YP00204551
78	cfa-miR-223	-181.72		YP00205120
82	cfa-miR-423a	-8.77		YP00205624
86	cfa-miR-486	-6.08		YP02119777
88	cfa-miR-8865	-5.78	A	YP02121101

Test Group	Control Group	Fold Regulation Threshold
Group 5	Control Group	2

Group 5 vs. Control Group

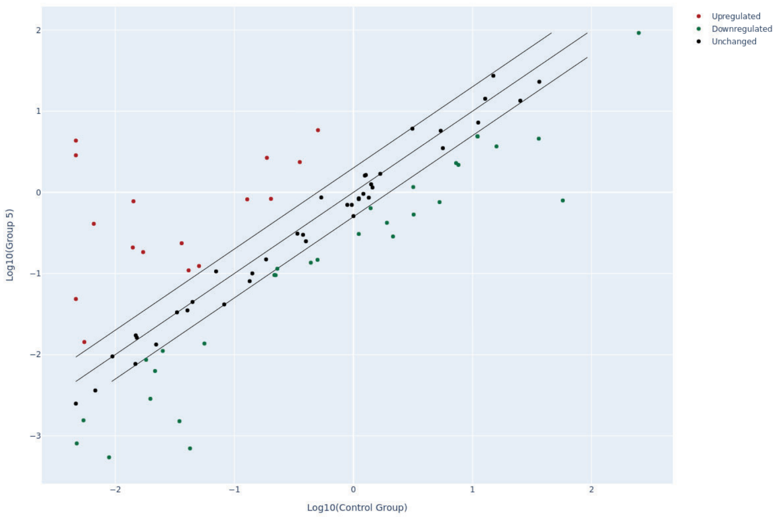

The Scatter Plot compares the normalized expression of every miRNA in the PCR Panel between the two selected Groups by plotting them against one another to quickly visualize large miRNA expression changes. The center diagonal line indicates unchanged miRNA expression, while the outer diagonal lines indicate the selected fold regulation threshold. miRNAs with data points beyond the outer lines in the upper left and lower right corners are up-regulated or down-regulated, respectively, by more than the fold regulation threshold in the y-axis Group relative to the x-axis Group.

miRNAs Over-Expressed in Group 5 vs. Control Group

Position	miRNA ID	Fold Regulation	Comments	miRCURY LNA miRNA PCR Assay Catalog #
3	cfa-let-7c	2.64	A	YP00204767
7	cfa-miR-10a	2.60	A	YP02113807
8	cfa-miR-10b	10.38	A	YP00205499
28	cfa-miR-30a	54.55	A	YP02101182
32	cfa-miR-34a	6.53	A	YP00204486
35	cfa-miR-99a	6.39	A	YP00205945
36	bta-miR-99b	14.90	A	YP00205983
37	cfa-miR-101	2.45	A	YP00205955
43	cfa-miR-125a	4.09		YP02113289
44	cfa-miR-125b	11.58		YP00205713
45	cfa-miR-126	6.66		YP00206010
52	cfa-miR-143	612.46	A	YP00205992
53	cfa-miR-145	928.49	A	YP00204483
68	cfa-miR-199	10.70	A	YP00205968
74	cfa-miR-214	62.01	A	YP00204510
85	cfa-miR-451	14.19		YP02119305

miRNAs Under-Expressed in Group 5 vs. Control Group

Position	miRNA ID	Fold Regulation	Comments	miRCURY LNA miRNA PCR Assay Catalog #
5	cfa-let-7f	-2.01		YP00204359
10	cfa-miR-15b	-3.61		YP00205964
16	cfa-miR-20a	-2.24		YP00204292
21	cfa-miR-25	-2.74		YP00204361
23	cfa-miR-26b	-4.32		YP00205953
30	cfa-miR-30d	-6.98		YP02118689
31	cfa-miR-31	-2.27		YP02119121
33	cfa-miR-92a	-7.86		YP00204258
34	cfa-miR-93	-3.50		YP00204715
38	cfa-miR-103	-2.27		YP00204063
39	cfa-miR-106a	-3.17		YP02107906
47	cfa-miR-128	-3.43	A	YP00205995
48	cfa-miR-130b	-4.09	A	YP00204317
57	cfa-miR-150	-2.70		YP00204660
59	cfa-miR-155	-6.90	B	YP02119772
60	cfa-miR-181a	-4.53		YP02117991
61	cfa-miR-181b	-2.33		YP02110378
63	cfa-miR-181d	-2.10	A	YP00204789
64	cfa-miR-182	-2.25	A	YP00206070
65	cfa-miR-183	-3.49	B	YP00206030
67	cfa-miR-197	-6.01		YP00204380
69	cfa-miR-200b	-5.90	B	YP00204144
73	cfa-miR-210	-3.39		YP02119434
78	cfa-miR-223	-72.21		YP00205120
81	cfa-miR-378	-3.23		YP00205946
82	cfa-miR-423a	-7.52		YP00205624
83	cfa-miR-450a	-22.87	B	YP02116559
84	cfa-miR-450b	-16.36	B	YP02106136
86	cfa-miR-486	-2.19		YP02119777
88	cfa-miR-8865	-60.75	B	YP02121101

Test Group	Control Group	Fold Regulation Threshold
Group 6	Control Group	2

Group 6 vs. Control Group

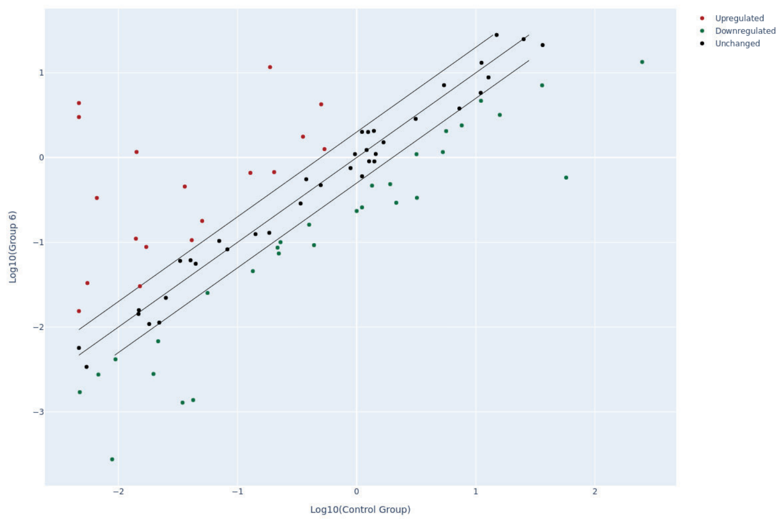

The Scatter Plot compares the normalized expression of every miRNA in the PCR Panel between the two selected Groups by plotting them against one another to quickly visualize large miRNA expression changes. The center diagonal line indicates unchanged miRNA expression, while the outer diagonal lines indicate the selected fold regulation threshold. miRNAs with data points beyond the outer lines in the upper left and lower right corners are up-regulated or down-regulated, respectively, by more than the fold regulation threshold in the y-axis Group relative to the x-axis Group.

miRNAs Over-Expressed in Group 6 vs. Control Group

Position	miRNA ID	Fold Regulation	Comments	miRCURY LNA miRNA PCR Assay Catalog #
3	cfa-let-7c	2.57	A	YP00204767
7	cfa-miR-10a	6.01	A	YP02113807
8	cfa-miR-10b	3.31	A	YP00205499
28	cfa-miR-30a	81.80	A	YP02101182
32	cfa-miR-34a	12.61	A	YP00204486
35	cfa-miR-99a	5.14	A	YP00205945
36	bta-miR-99b	7.87	A	YP00205983
37	cfa-miR-101	3.54	A	YP00205955
43	cfa-miR-125a	3.32		YP02113289
44	cfa-miR-125b	8.43		YP00205713
45	cfa-miR-126	4.98		YP00206010
52	cfa-miR-143	643.49	A	YP00205992
53	cfa-miR-145	940.67	A	YP00204483
55	cfa-miR-148a	2.34		YP00205867
68	cfa-miR-199	5.15	A	YP00205968
74	cfa-miR-214	50.56	A	YP00204510
75	cfa-miR-218	2.00	A	YP00206034
85	cfa-miR-451	62.15		YP02119305

miRNAs Under-Expressed in Group 6 vs. Control Group

Position	miRNA ID	Fold Regulation	Comments	miRCURY LNA miRNA PCR Assay Catalog #
5	cfa-let-7f	-2.29		YP00204359
10	cfa-miR-15b	-4.29		YP00205964
19	cfa-miR-23a	-2.94	A	YP00205956
20	cfa-miR-24	-2.75		YP02114589
21	cfa-miR-25	-2.90		YP00204361
23	cfa-miR-26b	-4.99		YP00205953
30	cfa-miR-30d	-4.55		YP02118689
31	cfa-miR-31	-2.51		YP02119121
33	cfa-miR-92a	-5.06		YP00204258
34	cfa-miR-93	-3.18		YP00204715
38	cfa-miR-103	-2.36		YP00204063
41	cfa-miR-107	-2.47		YP00205950
47	cfa-miR-128	-3.17	A	YP00205995
48	cfa-miR-130b	-2.22	A	YP00204317
51	cfa-miR-136	-2.27	B	YP00204779
56	cfa-miR-149	-2.47	B	YP00204321
57	cfa-miR-150	-18.53		YP00204660
59	cfa-miR-155	-7.04	B	YP02119772
60	cfa-miR-181a	-3.93		YP02117991
61	cfa-miR-181b	-3.01		YP02110378
67	cfa-miR-197	-9.55		YP00204380
69	cfa-miR-200b	-2.78	B	YP00204144
76	cfa-miR-221	-4.27		YP02104713
77	cfa-miR-222	-2.88		YP00204551
78	cfa-miR-223	-98.71		YP00205120
81	cfa-miR-378	-4.73		YP00205946
82	cfa-miR-423a	-7.33		YP00205624
83	cfa-miR-450a	-26.89	B	YP02116559
84	cfa-miR-450b	-32.19	B	YP02106136
88	cfa-miR-8865	-30.77	B	YP02121101

Volcano Plot

Test Group	Control Group	Fold Regulation Threshold	p-Value Threshold
Group 3	Control Group	2	0.05

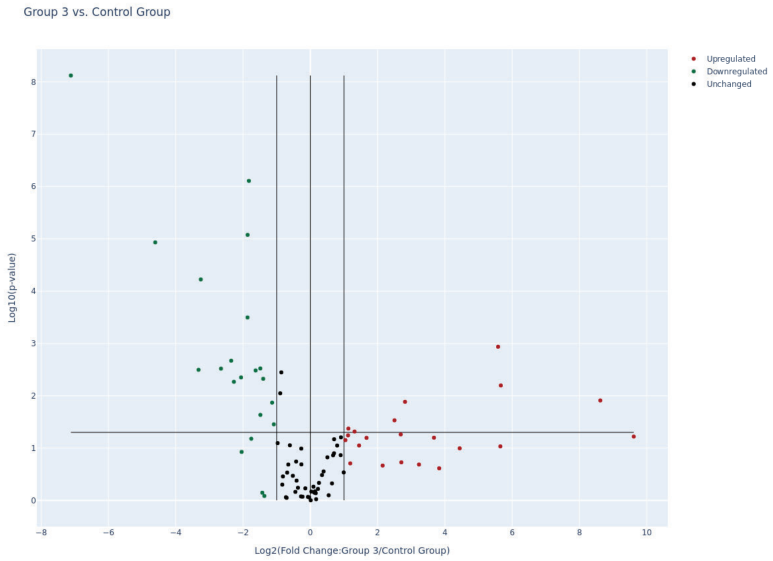

The Volcano Plot identifies significant miRNA expression changes by plotting the log2 of the fold changes in miRNA expression on the x-axis versus their statistical significance on the y-axis. The center vertical line indicates unchanged miRNA expression, while the two outer vertical lines indicate the selected fold regulation threshold. The horizontal line indicates the selected p-value threshold. miRNA with data points in the far upper left (down-regulated) and far upper right (up-regulated) sections meet the selected fold regulation and p-value thresholds. By combining the fold change results with the p-value statistical test results, miRNA with both large and small expression changes that are statistically significant are easily visualized.

miRNAs Over-Expressed in Group 3 vs. Control Group

Position	miRNA ID	Fold Regulation	p-Value	Comments	miRCURY LNA miRNA PCR Assay Catalog #
3	cfa-let-7c	2.73	0.089020	A	YP00204767
8	cfa-miR-10b	6.44	0.054755	A	YP00205499
12	cfa-miR-17	2.28	0.195735	A	YP00206008
13	cfa-miR-18a	2.49	0.048240		YP02119027
14	cfa-miR-19a	2.06	0.070382		YP00205862
28	cfa-miR-30a	47.83	0.001155	A	YP02101182
31	cfa-miR-31	12.77	0.063164		YP02119121
32	cfa-miR-34a	50.61	0.006348	A	YP00204486
35	cfa-miR-99a	5.66	0.029435	A	YP00205945
36	bta-miR-99b	14.21	0.243098	A	YP00205983
39	cfa-miR-106a	2.19	0.042318		YP02107906
43	cfa-miR-125a	4.44	0.215192		YP02113289
44	cfa-miR-125b	9.38	0.205902		YP00205713
45	cfa-miR-126	3.18	0.063612		YP00206010
52	cfa-miR-143	393.10	0.012269	A	YP00205992
53	cfa-miR-145	782.86	0.060148	A	YP00204483
68	cfa-miR-199	6.52	0.187048	A	YP00205968
74	cfa-miR-214	50.12	0.092577	A	YP00204510
80	cfa-miR-363	7.03	0.013027		YP02110319
85	cfa-miR-451	21.68	0.100959		YP02119305
87	cfa-miR-671	2.18	0.057105	A	YP00204024

miRNAs Under-Expressed in Group 3 vs. Control Group

Position	miRNA ID	Fold Regulation	p-Value	Comments	miRCURY LNA miRNA PCR Assay Catalog #
5	cfa-let-7f	-2.80	0.003003		YP00204359
10	cfa-miR-15b	-4.83	0.005393		YP00205964
19	cfa-miR-23a	-9.55	0.000060		YP00205956
20	cfa-miR-24	-3.54	0.000001		YP02114589
23	cfa-miR-26b	-5.11	0.002134		YP00205953
24	cfa-miR-27a	-3.64	0.000008		YP00205971
57	cfa-miR-150	-24.41	0.000012		YP00204660
58	cfa-miR-151	-2.20	0.013498		YP00204007
59	cfa-miR-155	-2.58	0.820157	B	YP02119772
60	cfa-miR-181a	-4.17	0.004455		YP02117991
61	cfa-miR-181b	-3.38	0.066199		YP02110378
62	cfa-miR-181c	-3.09	0.003276		YP02112709
63	cfa-miR-181d	-4.12	0.118223	B	YP00204789
67	cfa-miR-197	-2.80	0.023158		YP00204380
70	cfa-miR-203	-2.69	0.711210	B	YP00205914
76	cfa-miR-221	-2.64	0.004733		YP02104713
77	cfa-miR-222	-3.65	0.000318		YP00204551
78	cfa-miR-223	-138.82	0.000000		YP00205120
82	cfa-miR-423a	-2.12	0.035101		YP00205624
83	cfa-miR-450a	-10.01	0.003185		YP02116559
84	cfa-miR-450b	-6.30	0.003024		YP02106136

Test Group	Control Group	Fold Regulation Threshold	p-Value Threshold
Group 4	Control Group	2	0.05

Group 4 vs. Control Group

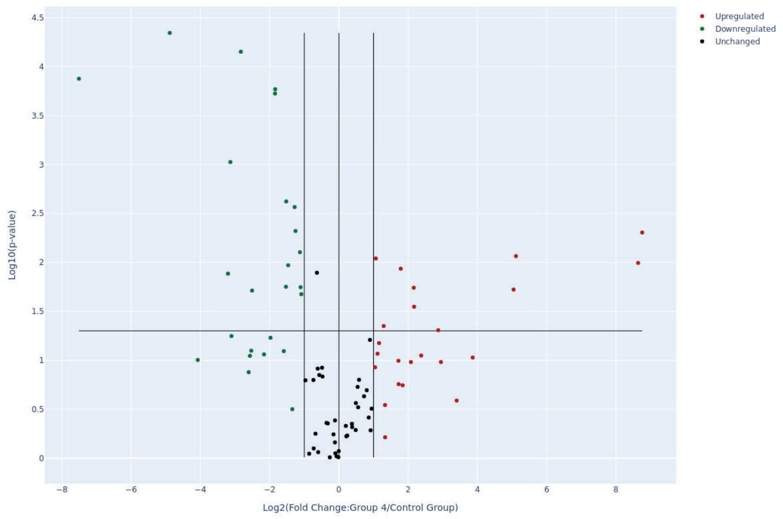

The Volcano Plot identifies significant miRNA expression changes by plotting the log2 of the fold changes in miRNA expression on the x-axis versus their statistical significance on the y-axis. The center vertical line indicates unchanged miRNA expression, while the two outer vertical lines indicate the selected fold regulation threshold. The horizontal line indicates the selected p-value threshold. miRNA with data points in the far upper left (down-regulated) and far upper right (up-regulated) sections meet the selected fold regulation and p-value thresholds. By combining the fold change results with the p-value statistical test results, miRNA with both large and small expression changes that are statistically significant are easily visualized.

miRNAs Over-Expressed in Group 4 vs. Control Group

Position	miRNA ID	Fold Regulation	p-Value	Comments	miRCURY LNA miRNA PCR Assay Catalog #
8	cfa-miR-10b	5.19	0.089344	A	YP00205499
12	cfa-miR-17	2.52	0.286360	A	YP00206008
13	cfa-miR-18a	2.09	0.009117		YP02119027
28	cfa-miR-30a	32.97	0.018914	A	YP02101182
32	cfa-miR-34a	4.50	0.028340	A	YP00204486
35	cfa-miR-99a	3.29	0.101100	A	YP00205945
36	bta-miR-99b	14.55	0.093688	A	YP00205983
37	cfa-miR-101	2.06	0.117694	A	YP00205955
40	cfa-miR-106b	2.23	0.066689		YP00205884
43	cfa-miR-125a	3.45	0.011589		YP02113289
44	cfa-miR-125b	4.47	0.018125		YP00205713
45	cfa-miR-126	3.31	0.175121		YP00206010
49	cfa-miR-132	7.71	0.104086	A	YP02104207
51	cfa-miR-136	2.52	0.610454	A	YP00204779
52	cfa-miR-143	432.19	0.004948	A	YP00205992
53	cfa-miR-145	398.57	0.010120	A	YP00204483
54	cfa-miR-146a	2.17	0.085501	A	YP00204688
60	cfa-miR-181a	2.45	0.044627		YP02117991
61	cfa-miR-181b	4.22	0.104151		YP02110378
64	cfa-miR-182	3.58	0.179820	A	YP00206070
68	cfa-miR-199	7.30	0.049231	A	YP00205968
74	cfa-miR-214	34.58	0.008617	A	YP00204510
85	cfa-miR-451	10.56	0.257530		YP02119305

miRNAs Under-Expressed in Group 4 vs. Control Group

Position	miRNA ID	Fold Regulation	p-Value	Comments	miRCURY LNA miRNA PCR Assay Catalog #
10	cfa-miR-15b	-4.47	0.086943		YP00205964
19	cfa-miR-23a	-5.68	0.019382	A	YP00205956
20	cfa-miR-24	-2.43	0.002720		YP02114589
21	cfa-miR-25	-2.12	0.021101		YP00204361
23	cfa-miR-26b	-3.93	0.058837		YP00205953
24	cfa-miR-27a	-2.15	0.017914		YP00205971
25	cfa-miR-29a	-3.58	0.000170		YP00204698
26	cfa-miR-29b	-2.88	0.017736		YP00204679
27	cfa-miR-29c	-2.38	0.004773		YP00204729
30	cfa-miR-30d	-3.59	0.000188		YP02118689
31	cfa-miR-31	-9.21	0.013013		YP02119121
33	cfa-miR-92a	-2.87	0.002382		YP00204258
41	cfa-miR-107	-2.18	0.007864		YP00205950
56	cfa-miR-149	-3.01	0.080524	B	YP00204321
57	cfa-miR-150	-29.51	0.000045		YP00204660
59	cfa-miR-155	-5.93	0.089992	B	YP02119772
67	cfa-miR-197	-7.12	0.000070		YP00204380
69	cfa-miR-200b	-16.84	0.099131	B	YP00204144
70	cfa-miR-203	-8.59	0.056525	B	YP00205914
71	cfa-miR-204	-2.54	0.315571	A	YP00206072
77	cfa-miR-222	-2.76	0.010684		YP00204551
78	cfa-miR-223	-181.72	0.000133		YP00205120
82	cfa-miR-423a	-8.77	0.000943		YP00205624
86	cfa-miR-486	-6.08	0.132204		YP02119777
88	cfa-miR-8865	-5.78	0.079792	A	YP02121101

Test Group	Control Group	Fold Regulation Threshold	p-Value Threshold
Group 5	Control Group	2	0.05

The Volcano Plot can not be generated when test or control group has less than 3 samples.

Test Group	Control Group	Fold Regulation Threshold	p-Value Threshold
Group 6	Control Group	2	0.05

The Volcano Plot can not be generated when test or control group has less than 3 samples.

Principle Component Analysis (PCA)

Number of Groups	3

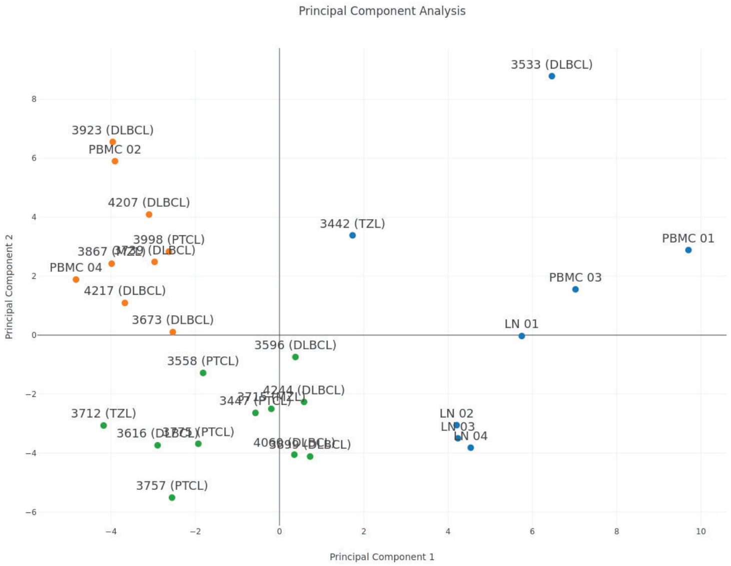

The Principal Component Analysis (PCA) statistically converts normalized miRNA expression levels into linearly uncorrelated values or principal components. The first principal component (x-axis) accounts the greatest amount of variability in the data, while the second principal component (y-axis) accounts for next greatest amount of variability. Each plot point represents a different Sample, and its color identifies the group into which the Sample has been placed by the PCA. For each Sample, the table lists its "Sample ID" (from S1 to Sn), Sample Name, its first and second principle component value ("PC1" and "PC2", respectively), and the "Group" to which it has been assigned. PCA may be used for exploratory data analysis by visualizing how Samples relate to one another based on their relative miRNA expression levels.

Clustergram

X-Axis	Dimension	Join Type	Color Coded
Sample	2-D	Average	Genes

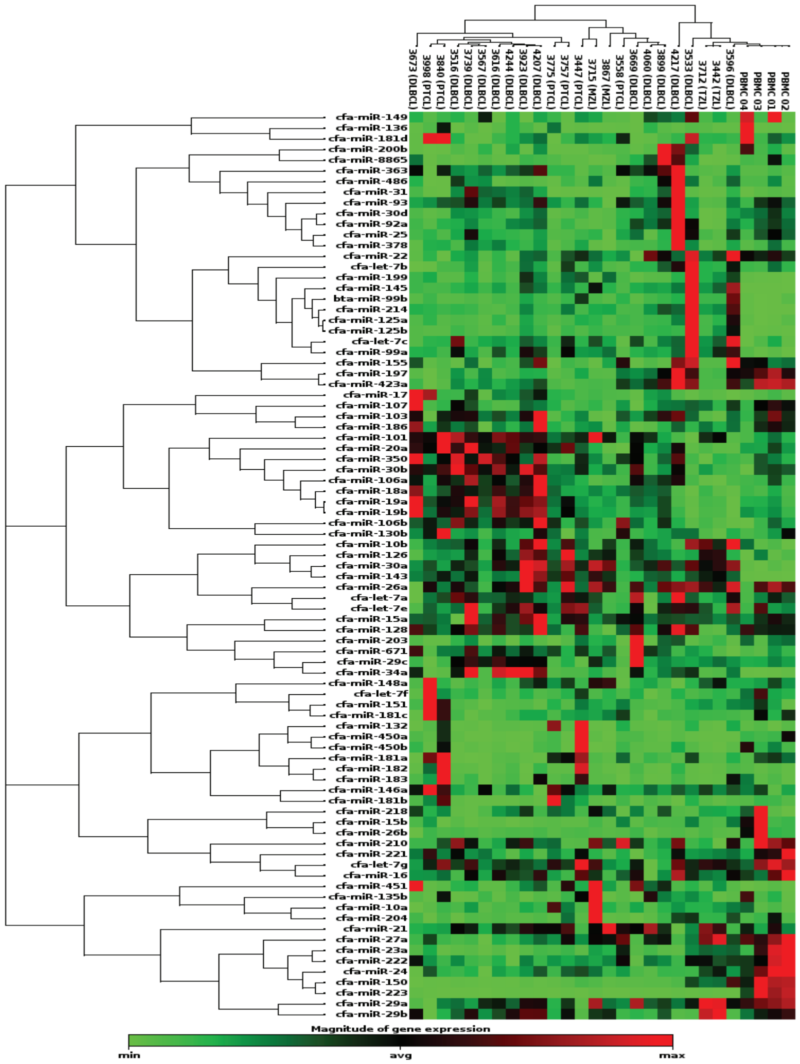

What's next

Thank you for using the miRCURY LNA miRNA PCR Data Analysis Software.

The Data Analysis software delivers a list of expression changes in the samples from the supplied data. However, this result often only starts an investigation into the underlying mechanisms at work. To assist in further analysis, the Data Analysis software utilizes the latest bioinformatics tools to analyze the data and suggest regulatory mechanisms and future experiments. Please review the results from the selected tools below.

miRNA Expression: This tool will help define a panel of miRNA based of this experiment’s results. This panel may represent a putative biomarker set, a target miRNA set or simply a collection of miRNAs. The tool is designed to deliver a list of miRNA expression assays that would allow the user to follow-up the results of the analyzed experiment.

miRNA Expression

Test Group	Control Group	Fold Regulation Threshold	p-Value Threshold
Group 3	Control Group	2	0.05

Position	miRNA ID	Fold Regulation	p-Value	miRCURY LNA miRNA PCR Assay Catalog #
13	cfa-miR-18a	2.49	0.04824	YP02119027
28	cfa-miR-30a	47.83	0.001155	YP02101182
32	cfa-miR-34a	50.61	0.006348	YP00204486
35	cfa-miR-99a	5.66	0.029435	YP00205945
39	cfa-miR-106a	2.19	0.042318	YP02107906
52	cfa-miR-143	393.1	0.012269	YP00205992
80	cfa-miR-363	7.03	0.013027	YP02110319
5	cfa-let-7f	-2.8	0.003003	YP00204359
10	cfa-miR-15b	-4.83	0.005393	YP00205964
19	cfa-miR-23a	-9.55	6e-05	YP00205956
20	cfa-miR-24	-3.54	1e-06	YP02114589
23	cfa-miR-26b	-5.11	0.002134	YP00205953
24	cfa-miR-27a	-3.64	8e-06	YP00205971
57	cfa-miR-150	-24.41	1.2e-05	YP00204660
58	cfa-miR-151	-2.2	0.013498	YP00204007
60	cfa-miR-181a	-4.17	0.004455	YP02117991
62	cfa-miR-181c	-3.09	0.003276	YP02112709
67	cfa-miR-197	-2.8	0.023158	YP00204380
76	cfa-miR-221	-2.64	0.004733	YP02104713
77	cfa-miR-222	-3.65	0.000318	YP00204551
78	cfa-miR-223	-138.82	0.0	YP00205120
82	cfa-miR-423a	-2.12	0.035101	YP00205624
83	cfa-miR-450a	-10.01	0.003185	YP02116559
84	cfa-miR-450b	-6.3	0.003024	YP02106136

Test Group	Control Group	Fold Regulation Threshold	p-Value Threshold
Group 4	Control Group	2	0.05

Position	miRNA ID	Fold Regulation	p-Value	miRCURY LNA miRNA PCR Assay Catalog #
13	cfa-miR-18a	2.09	0.009117	YP02119027
28	cfa-miR-30a	32.97	0.018914	YP02101182
32	cfa-miR-34a	4.5	0.02834	YP00204486
43	cfa-miR-125a	3.45	0.011589	YP02113289
44	cfa-miR-125b	4.47	0.018125	YP00205713
52	cfa-miR-143	432.19	0.004948	YP00205992
53	cfa-miR-145	398.57	0.01012	YP00204483
60	cfa-miR-181a	2.45	0.044627	YP02117991
68	cfa-miR-199	7.3	0.049231	YP00205968
74	cfa-miR-214	34.58	0.008617	YP00204510
19	cfa-miR-23a	-5.68	0.019382	YP00205956
20	cfa-miR-24	-2.43	0.00272	YP02114589
21	cfa-miR-25	-2.12	0.021101	YP00204361
24	cfa-miR-27a	-2.15	0.017914	YP00205971
25	cfa-miR-29a	-3.58	0.00017	YP00204698
26	cfa-miR-29b	-2.88	0.017736	YP00204679
27	cfa-miR-29c	-2.38	0.004773	YP00204729
30	cfa-miR-30d	-3.59	0.000188	YP02118689
31	cfa-miR-31	-9.21	0.013013	YP02119121
33	cfa-miR-92a	-2.87	0.002382	YP00204258
41	cfa-miR-107	-2.18	0.007864	YP00205950
57	cfa-miR-150	-29.51	4.5e-05	YP00204660
67	cfa-miR-197	-7.12	7e-05	YP00204380
77	cfa-miR-222	-2.76	0.010684	YP00204551
78	cfa-miR-223	-181.72	0.000133	YP00205120
82	cfa-miR-423a	-8.77	0.000943	YP00205624

Test Group	Control Group	Fold Regulation Threshold	p-Value Threshold
Group 5	Control Group	2	0.05

Position	miRNA ID	Fold Regulation	p-Value	miRCURY LNA miRNA PCR Assay Catalog #
3	cfa-let-7c	2.64	nan	YP00204767
7	cfa-miR-10a	2.6	nan	YP02113807
8	cfa-miR-10b	10.38	nan	YP00205499
28	cfa-miR-30a	54.55	nan	YP02101182
32	cfa-miR-34a	6.53	nan	YP00204486
35	cfa-miR-99a	6.39	nan	YP00205945
36	bta-miR-99b	14.9	nan	YP00205983
37	cfa-miR-101	2.45	nan	YP00205955
43	cfa-miR-125a	4.09	nan	YP02113289
44	cfa-miR-125b	11.58	nan	YP00205713
45	cfa-miR-126	6.66	nan	YP00206010
52	cfa-miR-143	612.46	nan	YP00205992
53	cfa-miR-145	928.49	nan	YP00204483
68	cfa-miR-199	10.7	nan	YP00205968
74	cfa-miR-214	62.01	nan	YP00204510
85	cfa-miR-451	14.19	nan	YP02119305
5	cfa-let-7f	-2.01	nan	YP00204359
10	cfa-miR-15b	-3.61	nan	YP00205964
16	cfa-miR-20a	-2.24	nan	YP00204292
21	cfa-miR-25	-2.74	nan	YP00204361
23	cfa-miR-26b	-4.32	nan	YP00205953
30	cfa-miR-30d	-6.98	nan	YP02118689
31	cfa-miR-31	-2.27	nan	YP02119121
33	cfa-miR-92a	-7.86	nan	YP00204258
34	cfa-miR-93	-3.5	nan	YP00204715
38	cfa-miR-103	-2.27	nan	YP00204063
39	cfa-miR-106a	-3.17	nan	YP02107906
47	cfa-miR-128	-3.43	nan	YP00205995
48	cfa-miR-130b	-4.09	nan	YP00204317
57	cfa-miR-150	-2.7	nan	YP00204660
59	cfa-miR-155	-6.9	nan	YP02119772
60	cfa-miR-181a	-4.53	nan	YP02117991
61	cfa-miR-181b	-2.33	nan	YP02110378
63	cfa-miR-181d	-2.1	nan	YP00204789
64	cfa-miR-182	-2.25	nan	YP00206070

Position	miRNA ID	Fold Regulation	p-Value	miRCURY LNA miRNA PCR Assay Catalog #
65	cfa-miR-183	-3.49	nan	YP00206030
67	cfa-miR-197	-6.01	nan	YP00204380
69	cfa-miR-200b	-5.9	nan	YP00204144
73	cfa-miR-210	-3.39	nan	YP02119434
78	cfa-miR-223	-72.21	nan	YP00205120
81	cfa-miR-378	-3.23	nan	YP00205946
82	cfa-miR-423a	-7.52	nan	YP00205624
83	cfa-miR-450a	-22.87	nan	YP02116559
84	cfa-miR-450b	-16.36	nan	YP02106136
86	cfa-miR-486	-2.19	nan	YP02119777
88	cfa-miR-8865	-60.75	nan	YP02121101

Test Group	Control Group	Fold Regulation Threshold	p-Value Threshold
Group 6	Control Group	2	0.05

Position	miRNA ID	Fold Regulation	p-Value	miRCURY LNA miRNA PCR Assay Catalog #
3	cfa-let-7c	2.57	nan	YP00204767
7	cfa-miR-10a	6.01	nan	YP02113807
8	cfa-miR-10b	3.31	nan	YP00205499
28	cfa-miR-30a	81.8	nan	YP02101182
32	cfa-miR-34a	12.61	nan	YP00204486
35	cfa-miR-99a	5.14	nan	YP00205945
36	bta-miR-99b	7.87	nan	YP00205983
37	cfa-miR-101	3.54	nan	YP00205955
43	cfa-miR-125a	3.32	nan	YP02113289
44	cfa-miR-125b	8.43	nan	YP00205713
45	cfa-miR-126	4.98	nan	YP00206010
52	cfa-miR-143	643.49	nan	YP00205992
53	cfa-miR-145	940.67	nan	YP00204483
55	cfa-miR-148a	2.34	nan	YP00205867
68	cfa-miR-199	5.15	nan	YP00205968
74	cfa-miR-214	50.56	nan	YP00204510
75	cfa-miR-218	2.0	nan	YP00206034
85	cfa-miR-451	62.15	nan	YP02119305
5	cfa-let-7f	-2.29	nan	YP00204359
10	cfa-miR-15b	-4.29	nan	YP00205964
19	cfa-miR-23a	-2.94	nan	YP00205956
20	cfa-miR-24	-2.75	nan	YP02114589
21	cfa-miR-25	-2.9	nan	YP00204361
23	cfa-miR-26b	-4.99	nan	YP00205953
30	cfa-miR-30d	-4.55	nan	YP02118689
31	cfa-miR-31	-2.51	nan	YP02119121
33	cfa-miR-92a	-5.06	nan	YP00204258
34	cfa-miR-93	-3.18	nan	YP00204715
38	cfa-miR-103	-2.36	nan	YP00204063
41	cfa-miR-107	-2.47	nan	YP00205950
47	cfa-miR-128	-3.17	nan	YP00205995
48	cfa-miR-130b	-2.22	nan	YP00204317
51	cfa-miR-136	-2.27	nan	YP00204779
56	cfa-miR-149	-2.47	nan	YP00204321
57	cfa-miR-150	-18.53	nan	YP00204660

Position	miRNA ID	Fold Regulation	p-Value	miRCURY LNA miRNA PCR Assay Catalog #
59	cfa-miR-155	-7.04	nan	YP02119772
60	cfa-miR-181a	-3.93	nan	YP02117991
61	cfa-miR-181b	-3.01	nan	YP02110378
67	cfa-miR-197	-9.55	nan	YP00204380
69	cfa-miR-200b	-2.78	nan	YP00204144
76	cfa-miR-221	-4.27	nan	YP02104713
77	cfa-miR-222	-2.88	nan	YP00204551
78	cfa-miR-223	-98.71	nan	YP00205120
81	cfa-miR-378	-4.73	nan	YP00205946
82	cfa-miR-423a	-7.33	nan	YP00205624
83	cfa-miR-450a	-26.89	nan	YP02116559
84	cfa-miR-450b	-32.19	nan	YP02106136
88	cfa-miR-8865	-30.77	nan	YP02121101

miRCURY LNA miRNA Expression Analysis Report 2

Sabine Hammer

03-03-2023

Table of Contents

Introduction 3

Summary and workflow 4

miRNA Table 5

Data analysis setup 6

Data quality control (QC) 8

Normalization analysis 10

Result 12

Fold regulation and p-value . . . 12

Scatter Plot . . . 18

Volcano Plot . . . 30

Principle Component Analysis (PCA) . . . 38

Clustergram . . . 39

What's next 40

miRNA Expression . . . 41

Introduction

Custom arrays

The Custom miRCURY LNA miRNA PCR Panels are reliable tools for studying and verifying miRNA expression biomarkers in large numbers of biological samples. Whether the biomarkers are from a proprietary collection, literature research or microarray analysis, QIAGEN's expertise in real-time RT-PCR detection provides quality, flexibility and consistency in biomarker validation experiments. Each Custom miRCURY LNA miRNA PCR Panel contains assays for a list of your chosen miRNAs of interest as well as for housekeeping (reference) miRNAs. In addition, each panel should also contain a set of proprietary controls to monitor first strand synthesis and real-time PCR efficiency. The qPCR Assays used in these panels are laboratory-verified and optimized to work under standard conditions enabling many miRNAs to be assayed simultaneously. Their specificity is guaranteed when a miRCURY LNA SYBR Green Master Mix is used as part of the complete RT-PCR system protocol.

In this study, 92 miRNAs were profiled on 32 samples.

Summary and workflow

Custom arrays

1. miRNA was isolated using an extraction kit per the manufacturer's instructions.
2. miRNA quality and quantity was determined and was reverse transcribed using the miRCURY LNA RT Kit.
3. The cDNA in combination with a miRCURY LNA Probe PCR Kit was used on a Custom miRCURY LNA miRNA Probe PCR Panel.

C_T values were exported to an Excel file to create a table of C_T values. This table was then uploaded on to the data analysis web portal at http://www.qiagen.com/geneglobe. Samples were assigned to controls and test groups. C_T values were normalized based on the geNorm (Pre-Defined Reference miRNAs Only) method.

The data analysis web portal calculates fold change/regulation using delta-delta C_T method, in which delta C_T is calculated between miRNA of interest and an average of reference miRNAs, followed by delta-delta C_T calculations (delta C_T (Test Group)-delta C_T (Control Group)). Fold Change is then calculated using 2^ (-delta-delta C_T) formula. The data analysis web portal also plots scatter plots, volcano plots, a principle component analysis and a clustergram.

The data analysis report was exported from the QIAGEN web portal at GeneGlobe.

miRNA Table

Position	miRNA ID	Position	miRNA ID	Position	miRNA ID	Position	miRNA ID
1	cfa-let-7a	2	cfa-let-7b	3	cfa-let-7c	4	cfa-let-7e
5	cfa-let-7f	6	cfa-let-7g	7	cfa-miR-10a	8	cfa-miR-10b
9	cfa-miR-15a	10	cfa-miR-15b	11	cfa-miR-16	12	cfa-miR-17
13	cfa-miR-18a	14	cfa-miR-19a	15	cfa-miR-19b	16	cfa-miR-20a
17	cfa-miR-21	18	cfa-miR-22	19	cfa-miR-23a	20	cfa-miR-24
21	cfa-miR-25	22	cfa-miR-26a	23	cfa-miR-26b	24	cfa-miR-27a
25	cfa-miR-29a	26	cfa-miR-29b	27	cfa-miR-29c	28	cfa-miR-30a
29	cfa-miR-30b	30	cfa-miR-30d	31	cfa-miR-31	32	cfa-miR-34a
33	cfa-miR-92a	34	cfa-miR-93	35	cfa-miR-99a	36	bta-miR-99b
37	cfa-miR-101	38	cfa-miR-103	39	cfa-miR-106a	40	cfa-miR-106b
41	cfa-miR-107	43	cfa-miR-125a	44	cfa-miR-125b	45	cfa-miR-126
47	cfa-miR-128	48	cfa-miR-130b	49	cfa-miR-132	50	cfa-miR-135b
51	cfa-miR-136	52	cfa-miR-143	53	cfa-miR-145	54	cfa-miR-146a
55	cfa-miR-148a	56	cfa-miR-149	57	cfa-miR-150	58	cfa-miR-151
59	cfa-miR-155	60	cfa-miR-181a	61	cfa-miR-181b	62	cfa-miR-181c
63	cfa-miR-181d	64	cfa-miR-182	65	cfa-miR-183	66	cfa-miR-186
67	cfa-miR-197	68	cfa-miR-199	69	cfa-miR-200b	70	cfa-miR-203
71	cfa-miR-204	73	cfa-miR-210	74	cfa-miR-214	75	cfa-miR-218
76	cfa-miR-221	77	cfa-miR-222	78	cfa-miR-223	79	cfa-miR-350
80	cfa-miR-363	81	cfa-miR-378	82	cfa-miR-423a	83	cfa-miR-450a
84	cfa-miR-450b	85	cfa-miR-451	86	cfa-miR-486	87	cfa-miR-671
88	cfa-miR-8865	90	UniSp2	92	UniSp4	94	UniSp5
96	UniSp6	98	cel-miR-39-3p	100	UniSP3	102	BlankSpot

Data analysis setup

Sample management

Sample ID	Sample Name	Group
1	LN 01	Control Group
2	LN 02	Control Group
3	LN 03	Control Group
4	LN 04	Control Group
5	PBMC 01	Exclude Sample
6	PBMC 02	Exclude Sample
7	PBMC 03	Exclude Sample
8	PBMC 04	Exclude Sample
9	4207 (DLBCL)	Group 3
10	3923 (DLBCL)	Group 3
11	3673 (DLBCL)	Group 3
12	3739 (DLBCL)	Group 3
13	3533 (DLBCL)	Group 3
14	4217 (DLBCL)	Group 3
15	3596 (DLBCL)	Group 3
16	3442 (TZL)	Group 5
17	3998 (PTCL)	Group 4
18	3867 (MZL)	Group 6
19	3757 (PTCL)	Group 4
20	3712 (TZL)	Group 5
21	3616 (DLBCL)	Group 3
22	3558 (PTCL)	Group 4
23	3447 (PTCL)	Group 4
24	4244 (DLBCL)	Group 3
25	4060 (DLBCL)	Group 3
26	3899 (DLBCL)	Group 3
27	3775 (PTCL)	Group 4
28	3715 (MZL)	Group 6
29	3840 (PTCL)	Group 4
30	3669 (DLBCL)	Group 3
31	3516 (DLBCL)	Group 3
32	3567 (DLBCL)	Group 3

Lower limit of detection

The C_T cut-off was set to 40

Data quality control (QC)

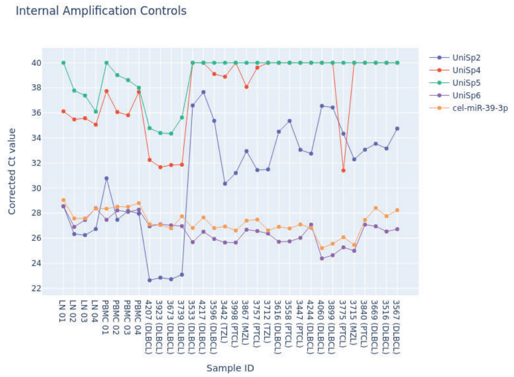

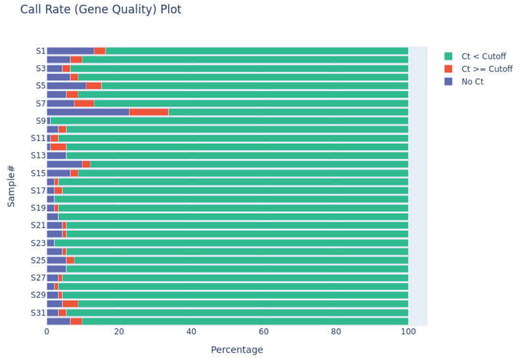

Sample#	Sample ID	Frequency of Missing Data
S1	LN 01	13.04%
S2	LN 02	6.52%
S3	LN 03	4.35%
S4	LN 04	6.52%
S5	PBMC 01	10.87%
S6	PBMC 02	5.43%
S7	PBMC 03	7.61%
S8	PBMC 04	22.83%
S9	4207 (DLBCL)	1.09%

Sample#	Sample ID	Frequency of Missing Data
S10	3923 (DLBCL)	3.26%
S11	3673 (DLBCL)	1.09%
S12	3739 (DLBCL)	1.09%
S13	3533 (DLBCL)	5.43%
S14	4217 (DLBCL)	9.78%
S15	3596 (DLBCL)	6.52%
S16	3442 (TZL)	2.17%
S17	3998 (PTCL)	2.17%
S18	3867 (MZL)	2.17%
S19	3757 (PTCL)	2.17%
S20	3712 (TZL)	3.26%
S21	3616 (DLBCL)	4.35%
S22	3558 (PTCL)	4.35%
S23	3447 (PTCL)	2.17%
S24	4244 (DLBCL)	4.35%
S25	4060 (DLBCL)	5.43%
S26	3899 (DLBCL)	5.43%
S27	3775 (PTCL)	3.26%
S28	3715 (MZL)	2.17%
S29	3840 (PTCL)	3.26%
S30	3669 (DLBCL)	4.35%
S31	3516 (DLBCL)	3.26%
S32	3567 (DLBCL)	6.52%

Normalization analysis

geNorm (Pre-Defined Reference miRNAs Only)

Groups	Samples	11	17	18	54	79	Arithmetic Mean	Average Arithmetic Mean
Stability Factor		0.2009	0.2015	0.2035	0.2034	0.2035		
Control Group	LN 01	30.66	30.22	36.55	34.95	37.16	33.91	31.75
Control Group	LN 02	28.69	29.10	36.65	33.42	38.53	33.28	
Control Group	LN 03	25.42	26.09	33.22	31.22	33.29	29.85	
Control Group	LN 04	26.32	26.47	32.90	31.00	33.17	29.97	
Group 3	3533 (DLBCL)	26.26	27.66	33.01	34.71	34.41	31.21	29.87
Group 3	3739 (DLBCL)	24.88	25.77	33.01	34.30	32.10	30.01	
Group 3	3596 (DLBCL)	25.61	27.93	32.49	33.85	33.91	30.76	
Group 3	3673 (DLBCL)	25.18	26.36	33.10	32.29	30.84	29.55	
Group 3	3923 (DLBCL)	24.13	24.34	31.69	32.88	31.40	28.89	
Group 3	4207 (DLBCL)	24.20	25.07	32.25	35.02	30.94	29.50	
Group 3	3567 (DLBCL)	25.18	26.22	32.95	32.09	30.86	29.46	
Group 3	3516 (DLBCL)	25.05	24.55	32.04	32.77	30.31	28.94	
Group 3	4217 (DLBCL)	27.30	30.99	35.76	36.12	34.70	32.97	
Group 3	4244 (DLBCL)	23.35	24.20	31.75	31.47	30.21	28.19	
Group 3	4060 (DLBCL)	26.76	25.24	32.30	33.50	33.40	30.24	
Group 3	3899 (DLBCL)	25.99	26.27	32.76	34.19	32.89	30.42	
Group 3	3669 (DLBCL)	23.84	24.67	32.70	32.98	31.08	29.05	
Group 3	3616 (DLBCL)	23.76	24.74	32.17	33.38	30.69	28.95	
Group 4	3998 (PTCL)	20.74	21.86	28.33	26.86	27.86	25.13	27.65
Group 4	3840 (PTCL)	22.57	23.84	30.84	29.52	29.24	27.20	
Group 4	3775 (PTCL)	23.74	23.64	31.46	30.25	31.22	28.06	
Group 4	3757 (PTCL)	24.57	23.84	31.23	31.19	31.87	28.54	
Group 4	3447 (PTCL)	22.78	25.47	30.76	31.67	30.40	28.22	
Group 4	3558 (PTCL)	24.09	24.28	31.74	32.73	31.06	28.78	
Group 5	3712 (TZL)	22.61	22.29	30.05	30.15	30.82	27.18	27.21
Group 5	3442 (TZL)	22.75	22.58	29.85	30.74	30.23	27.23	
Group 6	3867 (MZL)	23.88	22.56	30.89	30.55	31.11	27.80	27.66
Group 6	3715 (MZL)	22.62	23.15	30.32	31.30	30.21	27.52	

miRNA #	miRNA ID
11	cfa-miR-16
17	cfa-miR-21

miRNA #	miRNA ID
18	cfa-miR-22
54	cfa-miR-146a
79	cfa-miR-350

This method calculates a normalization factor based on multiple reference miRNA for more accurate and reliable normalization of miRNA expression data. It is based on the principle that the expression ratio of any two reference miRNAs (using standard deviation of log-transformed reference miRNA ratios) should be identical in all samples. Stability measures will be returned which are the average pairwise variation between a reference miRNA and all other reference miRNAs using stepwise exclusion of the worst scoring miRNAs. The predefined miRNAs or up to ten miRNAs from the entire panel with the best gNorm-calculated stability measures are listed if their value is less than 1.5. For more details, see:

Reference: Vandesompele J, De Preter K, Pattyn F, Poppe B, Van Roy N, De Paepe A, Speleman F. Accurate normalization of real-time quantitative RT-PCR data by geometric averaging of multiple internal control miRNAs. Genome Biol. 2002 Jun 18;3(7):RESEARCH0034. PubMed ID: 12184808.

Result

Fold regulation and p-value

Test Group	Control Group	Fold Regulation Threshold	p-Value Threshold
Group 3	Control Group	2	0.05

Position	miRNA ID	Fold Regulation	p-Value	Comments
13	cfa-miR-18a	5.74	0.017785	
14	cfa-miR-19a	4.21	0.028933	
15	cfa-miR-19b	2.83	0.044650	
16	cfa-miR-20a	4.77	0.006079	
32	cfa-miR-34a	14.68	0.008108	
34	cfa-miR-93	2.91	0.018803	
39	cfa-miR-106a	6.07	0.003385	
79	cfa-miR-350	3.19	0.006957	A
80	cfa-miR-363	14.63	0.008807	
7	cfa-miR-10a	-2.50	0.021758	
10	cfa-miR-15b	-3.31	0.011880	
19	cfa-miR-23a	-11.43	0.000000	A
20	cfa-miR-24	-2.62	0.000092	
23	cfa-miR-26b	-3.61	0.000722	
24	cfa-miR-27a	-2.45	0.002531	
54	cfa-miR-146a	-6.85	0.000001	
55	cfa-miR-148a	-2.41	0.034231	
57	cfa-miR-150	-10.98	0.018023	
59	cfa-miR-155	-10.92	0.000028	
62	cfa-miR-181c	-3.93	0.000530	
63	cfa-miR-181d	-8.66	0.004701	
76	cfa-miR-221	-2.66	0.002123	
77	cfa-miR-222	-2.98	0.001217	
78	cfa-miR-223	-8.93	0.003836	
82	cfa-miR-423a	-2.66	0.004074	
84	cfa-miR-450b	-2.34	0.043738	
87	cfa-miR-671	-5.17	0.025613	
88	cfa-miR-8865	-11.94	0.003054	A

Test Group	Control Group	Fold Regulation Threshold	p-Value Threshold
Group 4	Control Group	2	0.05

Position	miRNA ID	Fold Regulation	p-Value	Comments
13	cfa-miR-18a	4.83	0.000505	
14	cfa-miR-19a	3.81	0.013242	
15	cfa-miR-19b	2.60	0.028687	
16	cfa-miR-20a	4.89	0.024579	
34	cfa-miR-93	2.23	0.015880	
39	cfa-miR-106a	3.61	0.027982	
40	cfa-miR-106b	2.80	0.023745	
60	cfa-miR-181a	4.17	0.007311	
79	cfa-miR-350	2.25	0.031386	A
19	cfa-miR-23a	-6.80	0.001856	
23	cfa-miR-26b	-2.78	0.037517	
25	cfa-miR-29a	-3.24	0.001741	
27	cfa-miR-29c	-2.92	0.001318	
31	cfa-miR-31	-11.61	0.002270	
33	cfa-miR-92a	-2.01	0.020644	
54	cfa-miR-146a	-3.53	0.004609	
57	cfa-miR-150	-13.28	0.000105	
59	cfa-miR-155	-25.11	0.000169	
66	cfa-miR-186	-2.13	0.006575	
67	cfa-miR-197	-4.61	0.001497	
69	cfa-miR-200b	-16.27	0.031405	
77	cfa-miR-222	-2.26	0.008404	
82	cfa-miR-423a	-11.00	0.000563	
88	cfa-miR-8865	-42.31	0.048379	

Test Group	Control Group	Fold Regulation Threshold	p-Value Threshold
Group 5	Control Group	2	0.05

Position	miRNA ID	Fold Regulation	p-Value	Comments
8	cfa-miR-10b	3.93	nan	A
26	cfa-miR-29b	4.25	nan	
35	cfa-miR-99a	3.25	nan	
36	bta-miR-99b	2.80	nan	A
37	cfa-miR-101	2.00	nan	A
45	cfa-miR-126	4.73	nan	
52	cfa-miR-143	3.21	nan	
53	cfa-miR-145	3.93	nan	
68	cfa-miR-199	2.84	nan	A
75	cfa-miR-218	3.87	nan	A
85	cfa-miR-451	19.20	nan	
10	cfa-miR-15b	-2.47	nan	
19	cfa-miR-23a	-2.00	nan	
23	cfa-miR-26b	-3.05	nan	
30	cfa-miR-30d	-3.26	nan	
31	cfa-miR-31	-2.86	nan	
33	cfa-miR-92a	-5.51	nan	
47	cfa-miR-128	-2.66	nan	A
48	cfa-miR-130b	-2.68	nan	A
49	cfa-miR-132	-2.58	nan	A
54	cfa-miR-146a	-5.07	nan	
59	cfa-miR-155	-29.22	nan	B
60	cfa-miR-181a	-2.66	nan	
62	cfa-miR-181c	-2.52	nan	A
63	cfa-miR-181d	-4.41	nan	A
64	cfa-miR-182	-3.47	nan	A
65	cfa-miR-183	-7.46	nan	B
67	cfa-miR-197	-3.89	nan	
69	cfa-miR-200b	-5.70	nan	B
73	cfa-miR-210	-3.47	nan	
78	cfa-miR-223	-4.64	nan	
82	cfa-miR-423a	-9.42	nan	
83	cfa-miR-450a	-2.17	nan	B
84	cfa-miR-450b	-6.05	nan	B
87	cfa-miR-671	-11.18	nan	

Position	miRNA ID	Fold Regulation	p-Value	Comments
88	cfa-miR-8865	-444.91	nan	A

Test Group	Control Group	Fold Regulation Threshold	p-Value Threshold
Group 6	Control Group	2	0.05

Position	miRNA ID	Fold Regulation	p-Value	Comments
12	cfa-miR-17	3.35	nan	A
13	cfa-miR-18a	4.18	nan	
14	cfa-miR-19a	2.41	nan	
17	cfa-miR-21	2.03	nan	
28	cfa-miR-30a	2.33	nan	
32	cfa-miR-34a	3.66	nan	
35	cfa-miR-99a	2.62	nan	
37	cfa-miR-101	2.90	nan	A
45	cfa-miR-126	3.53	nan	
52	cfa-miR-143	3.37	nan	
53	cfa-miR-145	3.99	nan	
75	cfa-miR-218	7.28	nan	A
80	cfa-miR-363	3.06	nan	
85	cfa-miR-451	84.07	nan	
86	cfa-miR-486	5.27	nan	
10	cfa-miR-15b	-2.94	nan	
19	cfa-miR-23a	-3.52	nan	
20	cfa-miR-24	-2.03	nan	
23	cfa-miR-26b	-3.52	nan	
30	cfa-miR-30d	-2.12	nan	
31	cfa-miR-31	-3.16	nan	
33	cfa-miR-92a	-3.55	nan	
47	cfa-miR-128	-2.46	nan	A
49	cfa-miR-132	-3.04	nan	A
54	cfa-miR-146a	-5.18	nan	
57	cfa-miR-150	-8.34	nan	
59	cfa-miR-155	-29.79	nan	B
60	cfa-miR-181a	-2.31	nan	
63	cfa-miR-181d	-3.50	nan	A
65	cfa-miR-183	-3.41	nan	B
66	cfa-miR-186	-2.80	nan	
67	cfa-miR-197	-6.19	nan	
69	cfa-miR-200b	-2.67	nan	B
76	cfa-miR-221	-4.31	nan	
77	cfa-miR-222	-2.36	nan	

Position	miRNA ID	Fold Regulation	p-Value	Comments
78	cfa-miR-223	-6.35	nan	
81	cfa-miR-378	-2.09	nan	
82	cfa-miR-423a	-9.18	nan	
83	cfa-miR-450a	-2.55	nan	B
84	cfa-miR-450b	-11.91	nan	B
87	cfa-miR-671	-6.14	nan	
88	cfa-miR-8865	-225.39	nan	A

Fold-Change (2^(-Delta-Delta C_T)) is the normalized miRNA expression (2^(- Delta C_T)) in a Test Sample divided the normalized miRNA expression (2^(- Delta C_T)) in the Control Sample. Fold-Regulation represents fold-change results in a biologically meaningful way. Fold-change values greater than one indicates a positive- or an up-regulation, and the fold-regulation is equal to the fold-change. Fold-change values less than one indicate a negative or down-regulation, and the fold-regulation is the negative inverse of the fold-change.

The p values are calculated based on a Student's t-test of the replicate 2^(- Delta C_T) values for each miRNA in the Control Group and each Test Group, and p values less than 0.05 are indicated in red. The p-value calculation used is based on parametric, unpaired, two-sample equal variance, two-tailed distribution.

Scatter Plot

Test Group	Control Group	Fold Regulation Threshold
Group 3	Control Group	2

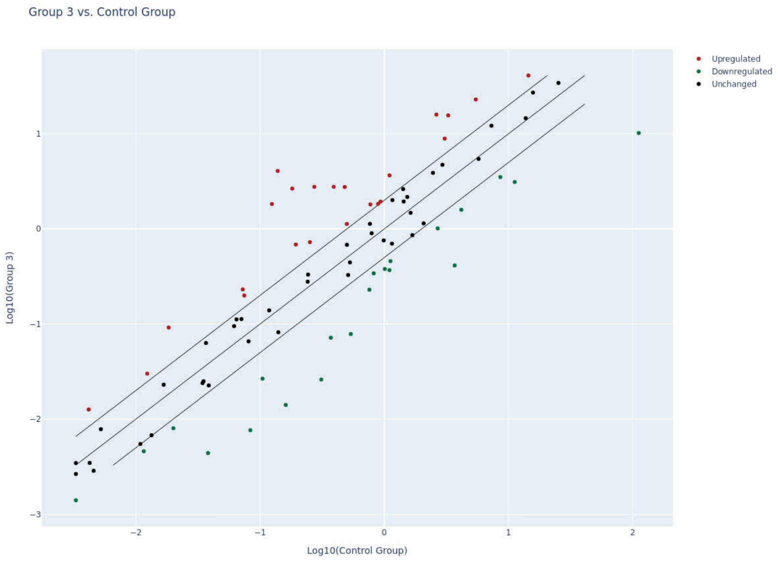

The Scatter Plot compares the normalized expression of every miRNA in the PCR Panel between the two selected Groups by plotting them against one another to quickly visualize large miRNA expression changes. The center diagonal line indicates unchanged miRNA expression, while the outer diagonal lines indicate the selected fold regulation threshold. miRNAs with data points beyond the outer lines in the upper left and lower right corners are up-regulated or down-regulated, respectively, by more than the fold regulation threshold in the y-axis Group relative to the x-axis Group.

miRNAs Over-Expressed in Group 3 vs. Control Group

Position	miRNA ID	Fold Regulation	Comments	miRCURY LNA miRNA PCR Assay Catalog #
8	cfa-miR-10b	2.44	A	YP00205499
12	cfa-miR-17	5.00	A	YP00206008
13	cfa-miR-18a	5.74		YP02119027
14	cfa-miR-19a	4.21		YP00205862
15	cfa-miR-19b	2.83		YP02105441
16	cfa-miR-20a	4.77		YP00204292
29	cfa-miR-30b	2.08		YP00204765
31	cfa-miR-31	10.13		YP02119121
32	cfa-miR-34a	14.68		YP00204486
34	cfa-miR-93	2.91		YP00204715
35	cfa-miR-99a	2.88		YP00205945
36	bta-miR-99b	2.68	A	YP00205983
39	cfa-miR-106a	6.07		YP02107906
40	cfa-miR-106b	2.34		YP00205884
45	cfa-miR-126	2.26		YP00206010
52	cfa-miR-143	2.06		YP00205992
53	cfa-miR-145	3.32		YP00204483
75	cfa-miR-218	3.03	B	YP00206034
79	cfa-miR-350	3.19	A	YP02111685
80	cfa-miR-363	14.63		YP02110319
81	cfa-miR-378	3.54		YP00205946
85	cfa-miR-451	29.33		YP02119305
86	cfa-miR-486	7.08		YP02119777

miRNAs Under-Expressed in Group 3 vs. Control Group

Position	miRNA ID	Fold Regulation	Comments	miRCURY LNA miRNA PCR Assay Catalog #
7	cfa-miR-10a	-2.50		YP02113807
10	cfa-miR-15b	-3.31		YP00205964
19	cfa-miR-23a	-11.43	A	YP00205956
20	cfa-miR-24	-2.62		YP02114589
23	cfa-miR-26b	-3.61		YP00205953
24	cfa-miR-27a	-2.45		YP00205971
54	cfa-miR-146a	-6.85		YP00204688
55	cfa-miR-148a	-2.41		YP00205867
57	cfa-miR-150	-10.98		YP00204660
59	cfa-miR-155	-10.92		YP02119772
60	cfa-miR-181a	-2.45		YP02117991
62	cfa-miR-181c	-3.93		YP02112709
63	cfa-miR-181d	-8.66		YP00204789
65	cfa-miR-183	-2.52	B	YP00206030
76	cfa-miR-221	-2.66		YP02104713
77	cfa-miR-222	-2.98		YP00204551
78	cfa-miR-223	-8.93		YP00205120
82	cfa-miR-423a	-2.66		YP00205624
84	cfa-miR-450b	-2.34		YP02106136
87	cfa-miR-671	-5.17		YP00204024
88	cfa-miR-8865	-11.94	A	YP02121101

Test Group	Control Group	Fold Regulation Threshold
Group 4	Control Group	2

Group 4 vs. Control Group

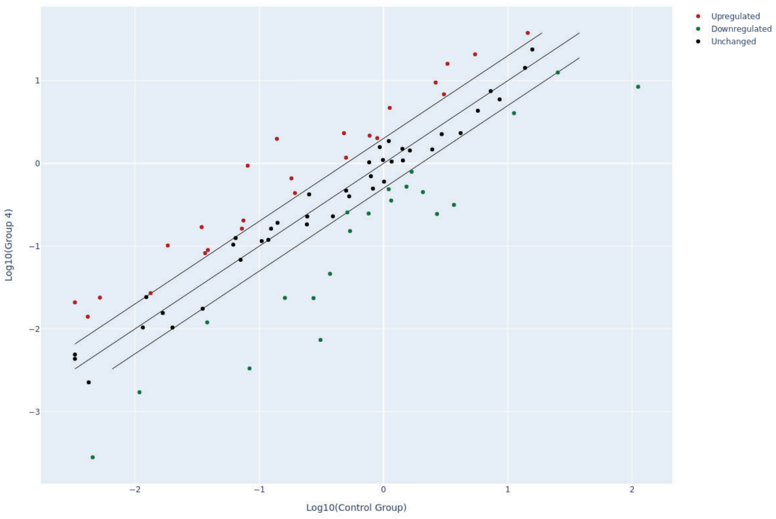

The Scatter Plot compares the normalized expression of every miRNA in the PCR Panel between the two selected Groups by plotting them against one another to quickly visualize large miRNA expression changes. The center diagonal line indicates unchanged miRNA expression, while the outer diagonal lines indicate the selected fold regulation threshold. miRNAs with data points beyond the outer lines in the upper left and lower right corners are up-regulated or down-regulated, respectively, by more than the fold regulation threshold in the y-axis Group relative to the x-axis Group.

miRNAs Over-Expressed in Group 4 vs. Control Group

Position	miRNA ID	Fold Regulation	Comments	miRCURY LNA miRNA PCR Assay Catalog #
12	cfa-miR-17	5.54	A	YP00206008
13	cfa-miR-18a	4.83		YP02119027
14	cfa-miR-19a	3.81		YP00205862
15	cfa-miR-19b	2.60		YP02105441
16	cfa-miR-20a	4.89		YP00204292
34	cfa-miR-93	2.23		YP00204715
36	bta-miR-99b	2.74	A	YP00205983
39	cfa-miR-106a	3.61		YP02107906
40	cfa-miR-106b	2.80		YP00205884
45	cfa-miR-126	2.35		YP00206010
48	cfa-miR-130b	2.25	A	YP00204317
49	cfa-miR-132	4.94	A	YP02104207
51	cfa-miR-136	4.57	A	YP00204779
52	cfa-miR-143	2.27		YP00205992
58	cfa-miR-151	2.02	A	YP00204007
60	cfa-miR-181a	4.17		YP02117991
61	cfa-miR-181b	11.62	A	YP02110378
64	cfa-miR-182	2.32	A	YP00206070
75	cfa-miR-218	3.37	A	YP00206034
79	cfa-miR-350	2.25	A	YP02111685
80	cfa-miR-363	3.63		YP02110319
81	cfa-miR-378	2.26		YP00205946
83	cfa-miR-450a	6.36	A	YP02116559
85	cfa-miR-451	14.28		YP02119305

miRNAs Under-Expressed in Group 4 vs. Control Group

Position	miRNA ID	Fold Regulation	Comments	miRCURY LNA miRNA PCR Assay Catalog #
10	cfa-miR-15b	-3.06		YP00205964
19	cfa-miR-23a	-6.80		YP00205956
23	cfa-miR-26b	-2.78		YP00205953
25	cfa-miR-29a	-3.24		YP00204698
27	cfa-miR-29c	-2.92		YP00204729
31	cfa-miR-31	-11.61		YP02119121
33	cfa-miR-92a	-2.01		YP00204258
54	cfa-miR-146a	-3.53		YP00204688
57	cfa-miR-150	-13.28		YP00204660
59	cfa-miR-155	-25.11		YP02119772
63	cfa-miR-181d	-3.17	A	YP00204789
66	cfa-miR-186	-2.13		YP00206053
67	cfa-miR-197	-4.61		YP00204380
69	cfa-miR-200b	-16.27		YP00204144
70	cfa-miR-203	-6.33	B	YP00205914
73	cfa-miR-210	-2.00		YP02119434
77	cfa-miR-222	-2.26		YP00204551
78	cfa-miR-223	-11.69		YP00205120
82	cfa-miR-423a	-11.00		YP00205624
87	cfa-miR-671	-8.01		YP00204024
88	cfa-miR-8865	-42.31		YP02121101

Test Group	Control Group	Fold Regulation Threshold
Group 5	Control Group	2

Group 5 vs. Control Group

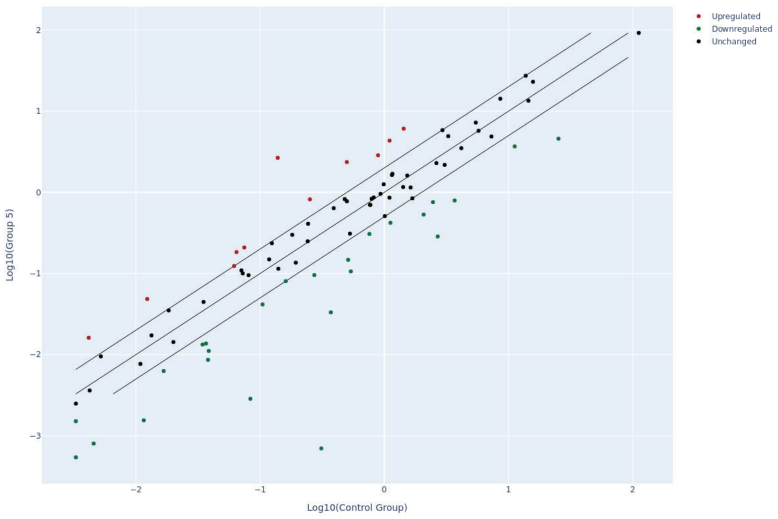

The Scatter Plot compares the normalized expression of every miRNA in the PCR Panel between the two selected Groups by plotting them against one another to quickly visualize large miRNA expression changes. The center diagonal line indicates unchanged miRNA expression, while the outer diagonal lines indicate the selected fold regulation threshold. miRNAs with data points beyond the outer lines in the upper left and lower right corners are up-regulated or down-regulated, respectively, by more than the fold regulation threshold in the y-axis Group relative to the x-axis Group.

miRNAs Over-Expressed in Group 5 vs. Control Group

Position	miRNA ID	Fold Regulation	Comments	miRCURY LNA miRNA PCR Assay Catalog #
8	cfa-miR-10b	3.93	A	YP00205499
26	cfa-miR-29b	4.25		YP00204679
35	cfa-miR-99a	3.25		YP00205945
36	bta-miR-99b	2.80	A	YP00205983
37	cfa-miR-101	2.00	A	YP00205955
45	cfa-miR-126	4.73		YP00206010
52	cfa-miR-143	3.21		YP00205992
53	cfa-miR-145	3.93		YP00204483
68	cfa-miR-199	2.84	A	YP00205968
75	cfa-miR-218	3.87	A	YP00206034
85	cfa-miR-451	19.20		YP02119305

miRNAs Under-Expressed in Group 5 vs. Control Group

Position	miRNA ID	Fold Regulation	Comments	miRCURY LNA miRNA PCR Assay Catalog #
10	cfa-miR-15b	-2.47		YP00205964
19	cfa-miR-23a	-2.00		YP00205956
23	cfa-miR-26b	-3.05		YP00205953
30	cfa-miR-30d	-3.26		YP02118689
31	cfa-miR-31	-2.86		YP02119121
33	cfa-miR-92a	-5.51		YP00204258
47	cfa-miR-128	-2.66	A	YP00205995
48	cfa-miR-130b	-2.68	A	YP00204317
49	cfa-miR-132	-2.58	A	YP02104207
54	cfa-miR-146a	-5.07		YP00204688
59	cfa-miR-155	-29.22	B	YP02119772
60	cfa-miR-181a	-2.66		YP02117991
62	cfa-miR-181c	-2.52	A	YP02112709
63	cfa-miR-181d	-4.41	A	YP00204789
64	cfa-miR-182	-3.47	A	YP00206070
65	cfa-miR-183	-7.46	B	YP00206030
67	cfa-miR-197	-3.89		YP00204380
69	cfa-miR-200b	-5.70	B	YP00204144
73	cfa-miR-210	-3.47		YP02119434
78	cfa-miR-223	-4.64		YP00205120
82	cfa-miR-423a	-9.42		YP00205624
83	cfa-miR-450a	-2.17	B	YP02116559
84	cfa-miR-450b	-6.05	B	YP02106136
87	cfa-miR-671	-11.18		YP00204024
88	cfa-miR-8865	-444.91	A	YP02121101

Test Group	Control Group	Fold Regulation Threshold
Group 6	Control Group	2

Group 6 vs. Control Group

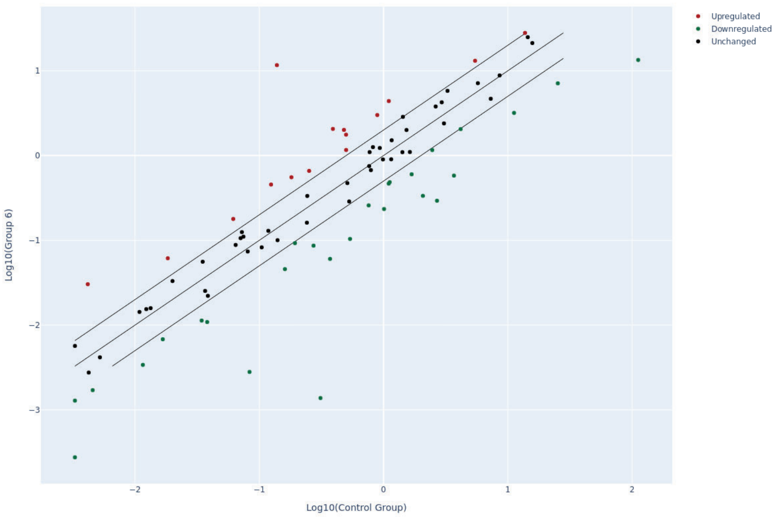

The Scatter Plot compares the normalized expression of every miRNA in the PCR Panel between the two selected Groups by plotting them against one another to quickly visualize large miRNA expression changes. The center diagonal line indicates unchanged miRNA expression, while the outer diagonal lines indicate the selected fold regulation threshold. miRNAs with data points beyond the outer lines in the upper left and lower right corners are up-regulated or down-regulated, respectively, by more than the fold regulation threshold in the y-axis Group relative to the x-axis Group.

miRNAs Over-Expressed in Group 6 vs. Control Group

Position	miRNA ID	Fold Regulation	Comments	miRCURY LNA miRNA PCR Assay Catalog #
12	cfa-miR-17	3.35	A	YP00206008
13	cfa-miR-18a	4.18		YP02119027
14	cfa-miR-19a	2.41		YP00205862
17	cfa-miR-21	2.03		YP00204230
28	cfa-miR-30a	2.33		YP02101182
32	cfa-miR-34a	3.66		YP00204486
35	cfa-miR-99a	2.62		YP00205945
37	cfa-miR-101	2.90	A	YP00205955
45	cfa-miR-126	3.53		YP00206010
52	cfa-miR-143	3.37		YP00205992
53	cfa-miR-145	3.99		YP00204483
75	cfa-miR-218	7.28	A	YP00206034
80	cfa-miR-363	3.06		YP02110319
85	cfa-miR-451	84.07		YP02119305
86	cfa-miR-486	5.27		YP02119777

miRNAs Under-Expressed in Group 6 vs. Control Group

Position	miRNA ID	Fold Regulation	Comments	miRCURY LNA miRNA PCR Assay Catalog #
10	cfa-miR-15b	-2.94		YP00205964
19	cfa-miR-23a	-3.52		YP00205956
20	cfa-miR-24	-2.03		YP02114589
23	cfa-miR-26b	-3.52		YP00205953
30	cfa-miR-30d	-2.12		YP02118689
31	cfa-miR-31	-3.16		YP02119121
33	cfa-miR-92a	-3.55		YP00204258
47	cfa-miR-128	-2.46	A	YP00205995
49	cfa-miR-132	-3.04	A	YP02104207
54	cfa-miR-146a	-5.18		YP00204688
57	cfa-miR-150	-8.34		YP00204660
59	cfa-miR-155	-29.79	B	YP02119772
60	cfa-miR-181a	-2.31		YP02117991
63	cfa-miR-181d	-3.50	A	YP00204789
65	cfa-miR-183	-3.41	B	YP00206030
66	cfa-miR-186	-2.80		YP00206053
67	cfa-miR-197	-6.19		YP00204380
69	cfa-miR-200b	-2.67	B	YP00204144
76	cfa-miR-221	-4.31		YP02104713
77	cfa-miR-222	-2.36		YP00204551
78	cfa-miR-223	-6.35		YP00205120
81	cfa-miR-378	-2.09		YP00205946
82	cfa-miR-423a	-9.18		YP00205624
83	cfa-miR-450a	-2.55	B	YP02116559
84	cfa-miR-450b	-11.91	B	YP02106136
87	cfa-miR-671	-6.14		YP00204024
88	cfa-miR-8865	-225.39	A	YP02121101

Volcano Plot

Test Group	Control Group	Fold Regulation Threshold	p-Value Threshold
Group 3	Control Group	2	0.05

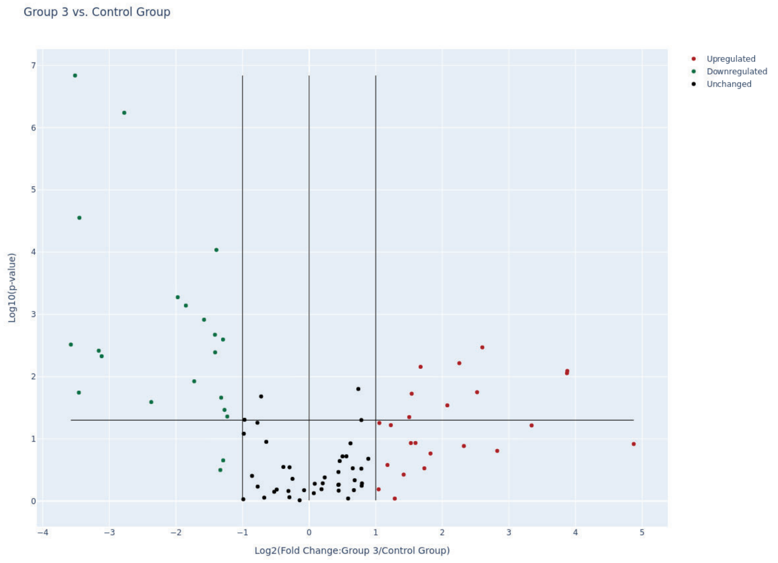

The Volcano Plot identifies significant miRNA expression changes by plotting the log2 of the fold changes in miRNA expression on the x-axis versus their statistical significance on the y-axis. The center vertical line indicates unchanged miRNA expression, while the two outer vertical lines indicate the selected fold regulation threshold. The horizontal line indicates the selected p-value threshold. miRNA with data points in the far upper left (down-regulated) and far upper right (up-regulated) sections meet the selected fold regulation and p-value thresholds. By combining the fold change results with the p-value statistical test results, miRNA with both large and small expression changes that are statistically significant are easily visualized.

miRNAs Over-Expressed in Group 3 vs. Control Group

Position	miRNA ID	Fold Regulation	p-Value	Comments	miRCURY LNA miRNA PCR Assay Catalog #
8	cfa-miR-10b	2.44	0.909560	A	YP00205499
12	cfa-miR-17	5.00	0.130191	A	YP00206008
13	cfa-miR-18a	5.74	0.017785		YP02119027
14	cfa-miR-19a	4.21	0.028933		YP00205862
15	cfa-miR-19b	2.83	0.044650		YP02105441
16	cfa-miR-20a	4.77	0.006079		YP00204292
29	cfa-miR-30b	2.08	0.055544		YP00204765
31	cfa-miR-31	10.13	0.060782		YP02119121
32	cfa-miR-34a	14.68	0.008108		YP00204486
34	cfa-miR-93	2.91	0.018803		YP00204715
35	cfa-miR-99a	2.88	0.116657		YP00205945
36	bta-miR-99b	2.68	0.374662	A	YP00205983
39	cfa-miR-106a	6.07	0.003385		YP02107906
40	cfa-miR-106b	2.34	0.060121		YP00205884
45	cfa-miR-126	2.26	0.262489		YP00206010
52	cfa-miR-143	2.06	0.645946		YP00205992
53	cfa-miR-145	3.32	0.296807		YP00204483
75	cfa-miR-218	3.03	0.116266	B	YP00206034
79	cfa-miR-350	3.19	0.006957	A	YP02111685
80	cfa-miR-363	14.63	0.008807		YP02110319
81	cfa-miR-378	3.54	0.171778		YP00205946
85	cfa-miR-451	29.33	0.120825		YP02119305
86	cfa-miR-486	7.08	0.155646		YP02119777

miRNAs Under-Expressed in Group 3 vs. Control Group

Position	miRNA ID	Fold Regulation	p-Value	Comments	miRCURY LNA miRNA PCR Assay Catalog #
7	cfa-miR-10a	-2.50	0.021758		YP02113807
10	cfa-miR-15b	-3.31	0.011880		YP00205964
19	cfa-miR-23a	-11.43	0.000000	A	YP00205956
20	cfa-miR-24	-2.62	0.000092		YP02114589
23	cfa-miR-26b	-3.61	0.000722		YP00205953
24	cfa-miR-27a	-2.45	0.002531		YP00205971
54	cfa-miR-146a	-6.85	0.000001		YP00204688
55	cfa-miR-148a	-2.41	0.034231		YP00205867
57	cfa-miR-150	-10.98	0.018023		YP00204660
59	cfa-miR-155	-10.92	0.000028		YP02119772
60	cfa-miR-181a	-2.45	0.221649		YP02117991
62	cfa-miR-181c	-3.93	0.000530		YP02112709
63	cfa-miR-181d	-8.66	0.004701		YP00204789
65	cfa-miR-183	-2.52	0.316120	B	YP00206030
76	cfa-miR-221	-2.66	0.002123		YP02104713
77	cfa-miR-222	-2.98	0.001217		YP00204551
78	cfa-miR-223	-8.93	0.003836		YP00205120
82	cfa-miR-423a	-2.66	0.004074		YP00205624
84	cfa-miR-450b	-2.34	0.043738		YP02106136
87	cfa-miR-671	-5.17	0.025613		YP00204024
88	cfa-miR-8865	-11.94	0.003054	A	YP02121101

Test Group	Control Group	Fold Regulation Threshold	p-Value Threshold
Group 4	Control Group	2	0.05

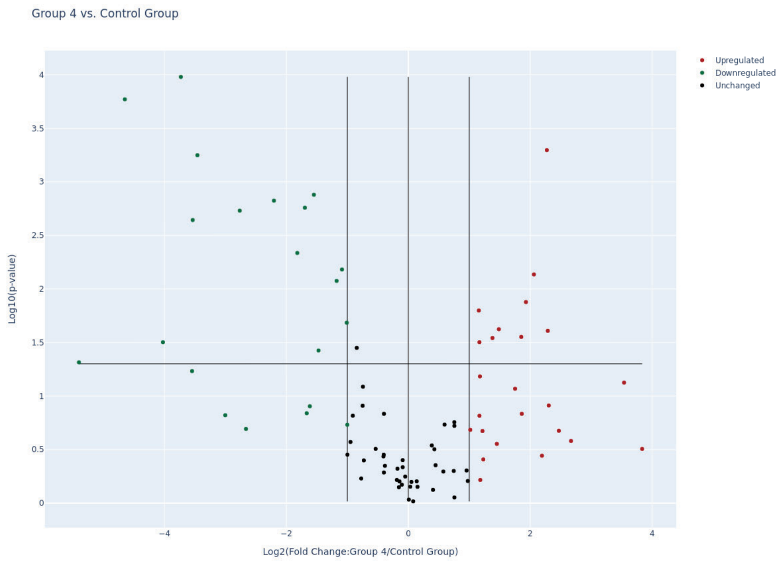

The Volcano Plot identifies significant miRNA expression changes by plotting the log2 of the fold changes in miRNA expression on the x-axis versus their statistical significance on the y-axis. The center vertical line indicates unchanged miRNA expression, while the two outer vertical lines indicate the selected fold regulation threshold. The horizontal line indicates the selected p-value threshold. miRNA with data points in the far upper left (down-regulated) and far upper right (up-regulated) sections meet the selected fold regulation and p-value thresholds. By combining the fold change results with the p-value statistical test results, miRNA with both large and small expression changes that are statistically significant are easily visualized.

miRNAs Over-Expressed in Group 4 vs. Control Group

Position	miRNA ID	Fold Regulation	p-Value	Comments	miRCURY LNA miRNA PCR Assay Catalog #
12	cfa-miR-17	5.54	0.210711	A	YP00206008
13	cfa-miR-18a	4.83	0.000505		YP02119027
14	cfa-miR-19a	3.81	0.013242		YP00205862
15	cfa-miR-19b	2.60	0.028687		YP02105441
16	cfa-miR-20a	4.89	0.024579		YP00204292
34	cfa-miR-93	2.23	0.015880		YP00204715
36	bta-miR-99b	2.74	0.279358	A	YP00205983
39	cfa-miR-106a	3.61	0.027982		YP02107906
40	cfa-miR-106b	2.80	0.023745		YP00205884
45	cfa-miR-126	2.35	0.390306		YP00206010
48	cfa-miR-130b	2.25	0.152544	A	YP00204317
49	cfa-miR-132	4.94	0.122374	A	YP02104207
51	cfa-miR-136	4.57	0.360465	A	YP00204779
52	cfa-miR-143	2.27	0.606833		YP00205992
58	cfa-miR-151	2.02	0.206487	A	YP00204007
60	cfa-miR-181a	4.17	0.007311		YP02117991
61	cfa-miR-181b	11.62	0.074820	A	YP02110378
64	cfa-miR-182	2.32	0.211825	A	YP00206070
75	cfa-miR-218	3.37	0.085407	A	YP00206034
79	cfa-miR-350	2.25	0.031386	A	YP02111685
80	cfa-miR-363	3.63	0.146332		YP02110319
81	cfa-miR-378	2.26	0.065484		YP00205946
83	cfa-miR-450a	6.36	0.262287	A	YP02116559
85	cfa-miR-451	14.28	0.311353		YP02119305

miRNAs Under-Expressed in Group 4 vs. Control Group

Position	miRNA ID	Fold Regulation	p-Value	Comments	miRCURY LNA miRNA PCR Assay Catalog #
10	cfa-miR-15b	-3.06	0.124417		YP00205964
19	cfa-miR-23a	-6.80	0.001856		YP00205956
23	cfa-miR-26b	-2.78	0.037517		YP00205953
25	cfa-miR-29a	-3.24	0.001741		YP00204698
27	cfa-miR-29c	-2.92	0.001318		YP00204729
31	cfa-miR-31	-11.61	0.002270		YP02119121
33	cfa-miR-92a	-2.01	0.020644		YP00204258
54	cfa-miR-146a	-3.53	0.004609		YP00204688
57	cfa-miR-150	-13.28	0.000105		YP00204660
59	cfa-miR-155	-25.11	0.000169		YP02119772
63	cfa-miR-181d	-3.17	0.144540	A	YP00204789
66	cfa-miR-186	-2.13	0.006575		YP00206053
67	cfa-miR-197	-4.61	0.001497		YP00204380
69	cfa-miR-200b	-16.27	0.031405		YP00204144
70	cfa-miR-203	-6.33	0.202500	B	YP00205914
73	cfa-miR-210	-2.00	0.185231		YP02119434
77	cfa-miR-222	-2.26	0.008404		YP00204551
78	cfa-miR-223	-11.69	0.058423		YP00205120
82	cfa-miR-423a	-11.00	0.000563		YP00205624
87	cfa-miR-671	-8.01	0.150947		YP00204024
88	cfa-miR-8865	-42.31	0.048379		YP02121101

Test Group	Control Group	Fold Regulation Threshold	p-Value Threshold
Group 5	Control Group	2	0.05

The Volcano Plot can not be generated when test or control group has less than 3 samples.

Test Group	Control Group	Fold Regulation Threshold	p-Value Threshold
Group 6	Control Group	2	0.05

The Volcano Plot can not be generated when test or control group has less than 3 samples.

Principle Component Analysis (PCA)

Number of Groups	3

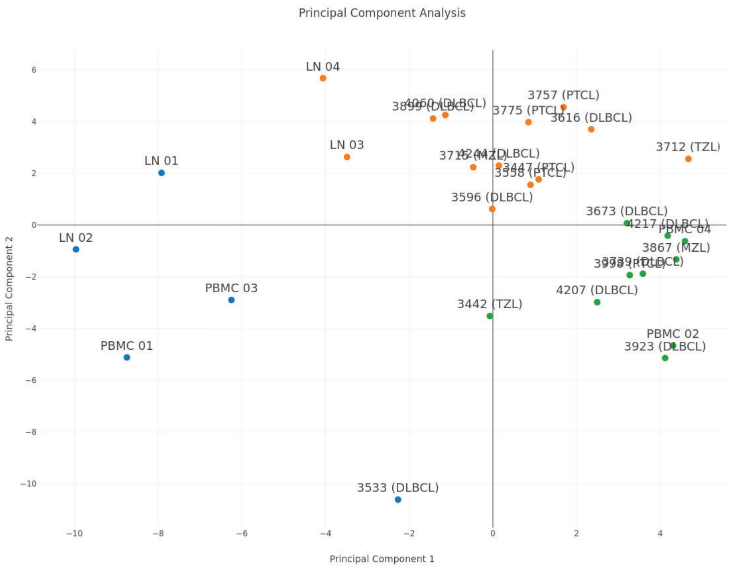

The Principal Component Analysis (PCA) statistically converts normalized miRNA expression levels into linearly uncorrelated values or principal components. The first principal component (x-axis) accounts the greatest amount of variability in the data, while the second principal component (y-axis) accounts for next greatest amount of variability. Each plot point represents a different Sample, and its color identifies the group into which the Sample has been placed by the PCA. For each Sample, the table lists its "Sample ID" (from S1 to Sn), Sample Name, its first and second principle component value ("PC1" and "PC2", respectively), and the "Group" to which it has been assigned. PCA may be used for exploratory data analysis by visualizing how Samples relate to one another based on their relative miRNA expression levels.

Clustergram

X-Axis	Dimension	Join Type	Color Coded
Sample	2-D	Average	Genes

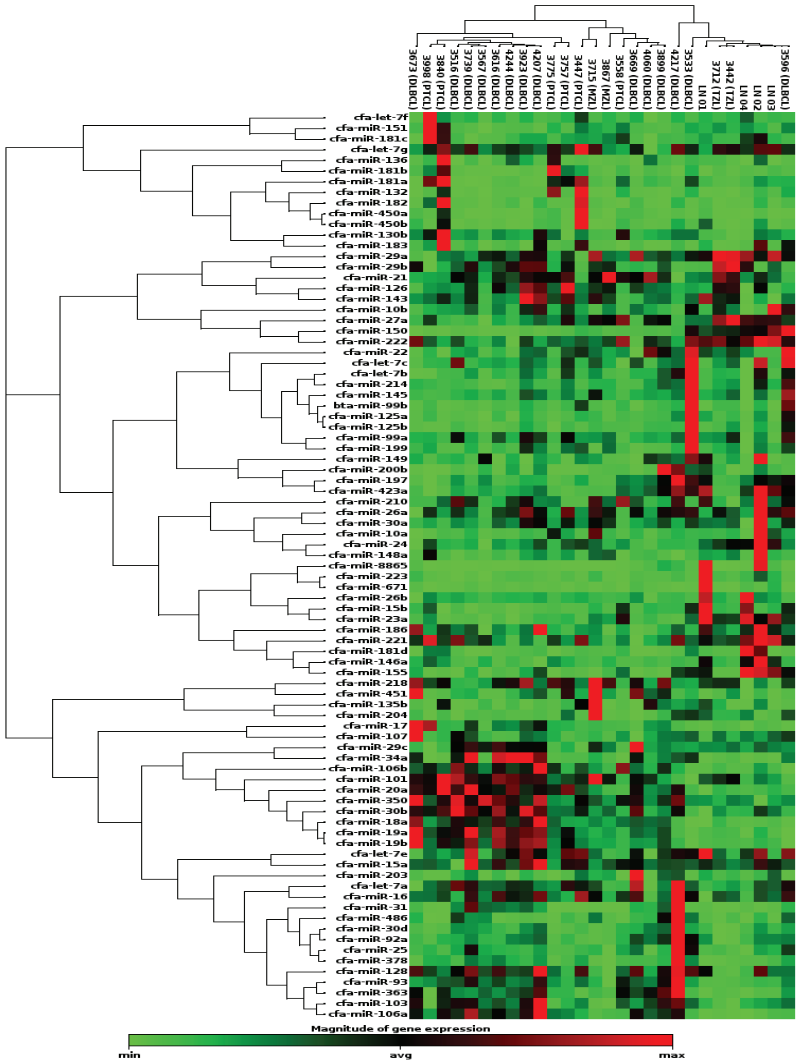

What's next

Thank you for using the miRCURY LNA miRNA PCR Data Analysis Software.

The Data Analysis software delivers a list of expression changes in the samples from the supplied data. However, this result often only starts an investigation into the underlying mechanisms at work. To assist in further analysis, the Data Analysis software utilizes the latest bioinformatics tools to analyze the data and suggest regulatory mechanisms and future experiments. Please review the results from the selected tools below.

miRNA Expression: This tool will help define a panel of miRNA based of this experiment's results. This panel may represent a putative biomarker set, a target miRNA set or simply a collection of miRNAs. The tool is designed to deliver a list of miRNA expression assays that would allow the user to follow-up the results of the analyzed experiment.

miRNA Expression

Test Group	Control Group	Fold Regulation Threshold	p-Value Threshold
Group 3	Control Group	2	0.05

Position	miRNA ID	Fold Regulation	p-Value	miRCURY LNA miRNA PCR Assay Catalog #
13	cfa-miR-18a	5.74	0.017785	YP02119027
14	cfa-miR-19a	4.21	0.028933	YP00205862
15	cfa-miR-19b	2.83	0.04465	YP02105441
16	cfa-miR-20a	4.77	0.006079	YP00204292
32	cfa-miR-34a	14.68	0.008108	YP00204486
34	cfa-miR-93	2.91	0.018803	YP00204715
39	cfa-miR-106a	6.07	0.003385	YP02107906
79	cfa-miR-350	3.19	0.006957	YP02111685
80	cfa-miR-363	14.63	0.008807	YP02110319
7	cfa-miR-10a	-2.5	0.021758	YP02113807
10	cfa-miR-15b	-3.31	0.01188	YP00205964
19	cfa-miR-23a	-11.43	0.0	YP00205956
20	cfa-miR-24	-2.62	9.2e-05	YP02114589
23	cfa-miR-26b	-3.61	0.000722	YP00205953
24	cfa-miR-27a	-2.45	0.002531	YP00205971
54	cfa-miR-146a	-6.85	1e-06	YP00204688
55	cfa-miR-148a	-2.41	0.034231	YP00205867
57	cfa-miR-150	-10.98	0.018023	YP00204660
59	cfa-miR-155	-10.92	2.8e-05	YP02119772
62	cfa-miR-181c	-3.93	0.00053	YP02112709
63	cfa-miR-181d	-8.66	0.004701	YP00204789
76	cfa-miR-221	-2.66	0.002123	YP02104713
77	cfa-miR-222	-2.98	0.001217	YP00204551
78	cfa-miR-223	-8.93	0.003836	YP00205120
82	cfa-miR-423a	-2.66	0.004074	YP00205624
84	cfa-miR-450b	-2.34	0.043738	YP02106136
87	cfa-miR-671	-5.17	0.025613	YP00204024
88	cfa-miR-8865	-11.94	0.003054	YP02121101

Test Group	Control Group	Fold Regulation Threshold	p-Value Threshold
Group 4	Control Group	2	0.05

Position	miRNA ID	Fold Regulation	p-Value	miRCURY LNA miRNA PCR Assay Catalog #
13	cfa-miR-18a	4.83	0.000505	YP02119027
14	cfa-miR-19a	3.81	0.013242	YP00205862
15	cfa-miR-19b	2.6	0.028687	YP02105441
16	cfa-miR-20a	4.89	0.024579	YP00204292
34	cfa-miR-93	2.23	0.01588	YP00204715
39	cfa-miR-106a	3.61	0.027982	YP02107906
40	cfa-miR-106b	2.8	0.023745	YP00205884
60	cfa-miR-181a	4.17	0.007311	YP02117991
79	cfa-miR-350	2.25	0.031386	YP02111685
19	cfa-miR-23a	-6.8	0.001856	YP00205956
23	cfa-miR-26b	-2.78	0.037517	YP00205953
25	cfa-miR-29a	-3.24	0.001741	YP00204698
27	cfa-miR-29c	-2.92	0.001318	YP00204729
31	cfa-miR-31	-11.61	0.00227	YP02119121
33	cfa-miR-92a	-2.01	0.020644	YP00204258
54	cfa-miR-146a	-3.53	0.004609	YP00204688
57	cfa-miR-150	-13.28	0.000105	YP00204660
59	cfa-miR-155	-25.11	0.000169	YP02119772
66	cfa-miR-186	-2.13	0.006575	YP00206053
67	cfa-miR-197	-4.61	0.001497	YP00204380
69	cfa-miR-200b	-16.27	0.031405	YP00204144
77	cfa-miR-222	-2.26	0.008404	YP00204551
82	cfa-miR-423a	-11.0	0.000563	YP00205624
88	cfa-miR-8865	-42.31	0.048379	YP02121101

Test Group	Control Group	Fold Regulation Threshold	p-Value Threshold
Group 5	Control Group	2	0.05

Position	miRNA ID	Fold Regulation	p-Value	miRCURY LNA miRNA PCR Assay Catalog #
8	cfa-miR-10b	3.93	nan	YP00205499
26	cfa-miR-29b	4.25	nan	YP00204679
35	cfa-miR-99a	3.25	nan	YP00205945
36	bta-miR-99b	2.8	nan	YP00205983
37	cfa-miR-101	2.0	nan	YP00205955
45	cfa-miR-126	4.73	nan	YP00206010
52	cfa-miR-143	3.21	nan	YP00205992
53	cfa-miR-145	3.93	nan	YP00204483
68	cfa-miR-199	2.84	nan	YP00205968
75	cfa-miR-218	3.87	nan	YP00206034
85	cfa-miR-451	19.2	nan	YP02119305
10	cfa-miR-15b	-2.47	nan	YP00205964
19	cfa-miR-23a	-2.0	nan	YP00205956
23	cfa-miR-26b	-3.05	nan	YP00205953
30	cfa-miR-30d	-3.26	nan	YP02118689
31	cfa-miR-31	-2.86	nan	YP02119121
33	cfa-miR-92a	-5.51	nan	YP00204258
47	cfa-miR-128	-2.66	nan	YP00205995
48	cfa-miR-130b	-2.68	nan	YP00204317
49	cfa-miR-132	-2.58	nan	YP02104207
54	cfa-miR-146a	-5.07	nan	YP00204688
59	cfa-miR-155	-29.22	nan	YP02119772
60	cfa-miR-181a	-2.66	nan	YP02117991
62	cfa-miR-181c	-2.52	nan	YP02112709
63	cfa-miR-181d	-4.41	nan	YP00204789
64	cfa-miR-182	-3.47	nan	YP00206070
65	cfa-miR-183	-7.46	nan	YP00206030
67	cfa-miR-197	-3.89	nan	YP00204380
69	cfa-miR-200b	-5.7	nan	YP00204144
73	cfa-miR-210	-3.47	nan	YP02119434
78	cfa-miR-223	-4.64	nan	YP00205120
82	cfa-miR-423a	-9.42	nan	YP00205624
83	cfa-miR-450a	-2.17	nan	YP02116559
84	cfa-miR-450b	-6.05	nan	YP02106136
87	cfa-miR-671	-11.18	nan	YP00204024

Position	miRNA ID	Fold Regulation	p-Value	miRCURY LNA miRNA PCR Assay Catalog #
88	cfa-miR-8865	-444.91	nan	YP02121101

Test Group	Control Group	Fold Regulation Threshold	p-Value Threshold
Group 6	Control Group	2	0.05

Position	miRNA ID	Fold Regulation	p-Value	miRCURY LNA miRNA PCR Assay Catalog #
12	cfa-miR-17	3.35	nan	YP00206008
13	cfa-miR-18a	4.18	nan	YP02119027
14	cfa-miR-19a	2.41	nan	YP00205862
17	cfa-miR-21	2.03	nan	YP00204230
28	cfa-miR-30a	2.33	nan	YP02101182
32	cfa-miR-34a	3.66	nan	YP00204486
35	cfa-miR-99a	2.62	nan	YP00205945
37	cfa-miR-101	2.9	nan	YP00205955
45	cfa-miR-126	3.53	nan	YP00206010
52	cfa-miR-143	3.37	nan	YP00205992
53	cfa-miR-145	3.99	nan	YP00204483
75	cfa-miR-218	7.28	nan	YP00206034
80	cfa-miR-363	3.06	nan	YP02110319
85	cfa-miR-451	84.07	nan	YP02119305
86	cfa-miR-486	5.27	nan	YP02119777
10	cfa-miR-15b	-2.94	nan	YP00205964
19	cfa-miR-23a	-3.52	nan	YP00205956
20	cfa-miR-24	-2.03	nan	YP02114589
23	cfa-miR-26b	-3.52	nan	YP00205953
30	cfa-miR-30d	-2.12	nan	YP02118689
31	cfa-miR-31	-3.16	nan	YP02119121
33	cfa-miR-92a	-3.55	nan	YP00204258
47	cfa-miR-128	-2.46	nan	YP00205995
49	cfa-miR-132	-3.04	nan	YP02104207
54	cfa-miR-146a	-5.18	nan	YP00204688
57	cfa-miR-150	-8.34	nan	YP00204660
59	cfa-miR-155	-29.79	nan	YP02119772
60	cfa-miR-181a	-2.31	nan	YP02117991
63	cfa-miR-181d	-3.5	nan	YP00204789
65	cfa-miR-183	-3.41	nan	YP00206030
66	cfa-miR-186	-2.8	nan	YP00206053
67	cfa-miR-197	-6.19	nan	YP00204380
69	cfa-miR-200b	-2.67	nan	YP00204144
76	cfa-miR-221	-4.31	nan	YP02104713
77	cfa-miR-222	-2.36	nan	YP00204551

Position	miRNA ID	Fold Regulation	p-Value	miRCURY LNA miRNA PCR Assay Catalog #
78	cfa-miR-223	-6.35	nan	YP00205120
81	cfa-miR-378	-2.09	nan	YP00205946
82	cfa-miR-423a	-9.18	nan	YP00205624
83	cfa-miR-450a	-2.55	nan	YP02116559
84	cfa-miR-450b	-11.91	nan	YP02106136
87	cfa-miR-671	-6.14	nan	YP00204024
88	cfa-miR-8865	-225.39	nan	YP02121101
